# Survival Analysis with High-Dimensional Omics Data Using a Threshold Gradient Descent Regularization-Based Neural Network Approach

**DOI:** 10.3390/genes13091674

**Published:** 2022-09-19

**Authors:** Yu Fan, Sanguo Zhang, Shuangge Ma

**Affiliations:** 1 School of Mathematics Sciences, University of Chinese Academy of Sciences, Beijing 100049, China; 2 Key Laboratory of Big Data Mining and Knowledge Management, Chinese Academy of Sciences, Beijing 100190, China; 3 Department of Biostatistics, Yale School of Public Health, New Haven, CT 06511, USA

**Keywords:** survival analysis, high-dimensional omics data, neural network, TGDR

## Abstract

Analysis of data with a censored survival response and high-dimensional omics measurements is now common. Most of the existing analyses are based on specific (semi)parametric models, in particular the Cox model. Such analyses may be limited by not having sufficient flexibility, for example, in accommodating nonlinearity. For categorical and continuous responses, neural networks (NNs) have provided a highly competitive alternative. Comparatively, NNs for censored survival data remain limited. Omics measurements are usually high-dimensional, and only a small subset is expected to be survival-associated. As such, regularized estimation and selection are needed. In the existing NN studies, this is usually achieved via penalization. In this article, we propose adopting the threshold gradient descent regularization (TGDR) technique, which has competitive performance (for example, when compared to penalization) and unique advantages in regression analysis, but has not been adopted with NNs. The TGDR-based NN has a highly sensible formulation and an architecture different from the unregularized and penalization-based ones. Simulations show its satisfactory performance. Its practical effectiveness is further established via the analysis of two cancer omics datasets. Overall, this study can provide a practical and useful new way in the NN paradigm for survival analysis with high-dimensional omics measurements.

## 1. Introduction

In the study of complex diseases, data with a censored survival response (overall survival, progression-free survival, time to metastasis, etc.) and high-dimensional omics measurements (gene expression, SNPs, methylation loci, etc.) are now routine. When the dimensionality of omics measurements is high (compared to sample size), regularized estimation is needed. For many practical scenarios (for example, as in our data analysis), it is expected that only a subset of the omics measurements is associated with survival. As such, variable/model selection is needed. In the past two decades, many regularized estimation and selection methods have been developed, and techniques extensively adopted include penalization [1,2], thresholding [3], sparse boosting [4], Bayesian [5], and others [6,7]. Most of the existing studies are based on specific (semi)parametric models, especially the Cox model [8]. Here, we note that some “model-free” methods, for example, the one based on U-statistic [9], are still based on albeit very weak model assumptions.

In recent years, it has been increasingly recognized that model-based approaches may not be sufficiently flexible, especially in accommodating unknown nonlinear effects. The neural network (NN) technique [10], with its superior flexibility and prediction performance, has drawn increasing attention and provided a highly competitive alternative to regression. Most of the existing NNs are for categorical and continuous responses [11,12,13,14,15]. Comparatively, NNs for censored survival data remain limited. Motivated by the increasing popularity of data with high-dimensional omics (and other types of) measurements, some of the recent NN studies have incorporated regularization in building networks (estimating network weights) [16,17,18]. In these studies, the most popular choice is penalization. For example, ridge penalization has been adopted to regularize weight estimation and increase stability [19]. lasso penalization has been applied to the input layer to achieve sparsity, with which only a subset of input variables contributes to response [20].

There are also a handful of NN studies for censored survival data. Examples include Cox-nnet, which “combines” the Cox model with NN [21]. The output of this NN is log hazard ratio, and log partial likelihood is adopted as the loss function. A similar approach was proposed in Sun et al. [22]. Considering a sequence pattern in the feature space, Ren et al. [23] proposed a deep recurrent survival analysis (DRSA) model that combines a recurrent neural network with the Cox model. This approach can predict conditional probability at a fine-grained level of data. Lee et al. [24] proposed an alternative survival analysis method, DeepHit, which can learn the distribution of survival time. DeepHit is a multitask network and consists of a shared subnetwork and multiple cause-specific subnetworks. Furthermore, it has a unique loss function that combines the log likelihood of the joint distribution of observed times and events and a combination of cause-specific ranking loss functions. For pathological imaging data, an effective whole slide histopathological images survival analysis framework (WSISA) was proposed by Zhu et al. [25]. The framework includes segmentation, clustering, and convolution network training of candidate patches. It can learn survival-related patterns from histopathological images. Here, it is noted that this approach may be applicable to omics data with minor modifications. With high-dimensional omics data, extensive unimportant measurements are expected, which need to be accommodated by sparse NN architectures. Yang et al. [26] constructed an NN with sample-wise sparsity using a gating network. Hao et al. [27] introduced gene pathways as prior information to build a locally connected NN. Yin et al. [28] combined a convolutional NN with prognosis-related cascaded Wx feature selection to achieve survival prediction.

In regression analysis, it has been well established that different regularization techniques have different advantages and disadvantages, with none dominating all others. In the NN paradigm, our literature review suggests that most regularized estimations adopt penalization. The goal of this study was to introduce an alternative regularization technique, threshold gradient descent regularization (TGDR) [3], to NN analysis of data with a censored survival response and high-dimensional omics measurements. In regression analysis of multiple types of responses under various models, it has been shown that TGDR has an intuitive formulation, simpler computation, and satisfactory estimation and selection performance. Beyond “standard” settings, it has also been extended to complex data/model settings, for example, longitudinal data [29], interaction analysis [30], multidataset analysis [31], and others [32]. It has been shown that, for many data settings, TGDR outperforms penalization, Bayesian, boosting, and other techniques. TGDR has a rationale/formulation fundamentally different from the other regularization techniques. Accordingly, its results, for example, NN architecture, are expected to be significantly different. With its competitive performance in regression, it is warranted to develop the TGDR-based NN technique.

## 2. Materials and Methods

### 2.1. Data and Architecture

Assume *N* independent and identically distributed samples. For the *i*th sample, denote $z_{i}$ as the *p*-dimensional vector of omics measurements. The observed response is $\left\{Y_{i},\delta_{i}\right\}$, where $Y_{i}=\min(T_{i},C_{i})$, $\delta_{i}=I\left(T_{i}\leq C_{i}\right)$, and $T_{i}$,$C_{i}$ are the event and censoring times, respectively. Many of the existing NN losses have been motivated by the log-likelihood functions of regression models [33,34]. With a censored survival response, we consider the Cox model, which is perhaps the most popular survival model and has conditional hazard function:

(1) $$\lambda (t|z_{i})=\lambda_{0}\left(t\right)\exp(\beta^{T}z_{i}),$$

where $\lambda_{0}\left(t\right)$ is the unknown baseline hazard function, and β is the vector of unknown regression coefficients. The log partial likelihood function is:

(2) $$l\left(\beta \right)=\sum_{\delta_{i}=1}\left(\beta^{T}z_{i}-\log\sum_{t_{j}\geq t_{i}}\exp(\beta^{T}z_{j})\right).$$

Following the strategy of quite a few published studies, we build an NN with a loss function motivated by the above log partial likelihood function. The proposed NN consists of one input layer, one or multiple hidden layers, and one Cox output layer. For each sample, we feed the *p*-dimensional vector of measurements $z_{i}$ into the input layer and obtain the log hazard radio $\theta_{i}$ from the output layer. To simplify notation, we first describe using one hidden layer. For the hidden layer with *h* nodes, the tanh activation function is used, which leads to satisfactory performance in our numerical study—here, we note that other activation functions may be needed for other data settings. The forward propagation of the entire NN is:

(3) $$\theta_{i}=\beta^{T}\sigma \left(Wz_{i}+b\right),$$

where *W* is the weight matrix between the input layer and hidden layer, with size *h* × *p*, *b* the bias term for the hidden layer and *σ* the tanh activation function:

(4) $$\sigma \left(a\right)=\frac{e^{a}-e^{-a}}{e^{a}+e^{-a}}.$$

**Figure A1:**
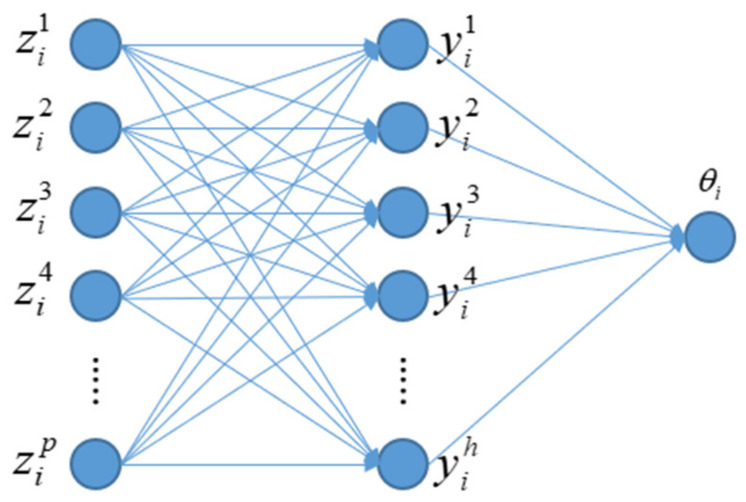
Structure diagram of the proposed NN architecture (prior to regularized estimation).

Finally, for the cost part of the network, we adopt:

(5) $$l\left(W,\beta \right)=\sum_{\delta_{i}=1}\left(\theta_{i}-\log\sum_{t_{j}\geq t_{i}}\exp(\theta_{j})\right),$$

which has been motivated by the log partial likelihood. This loss has also been adopted in some recent literature [21,22]. The overall structure of the proposed network is shown in Figure A1 (Appendix A).

### 2.2. TGDR-Based Optimization

**Figure 1:**
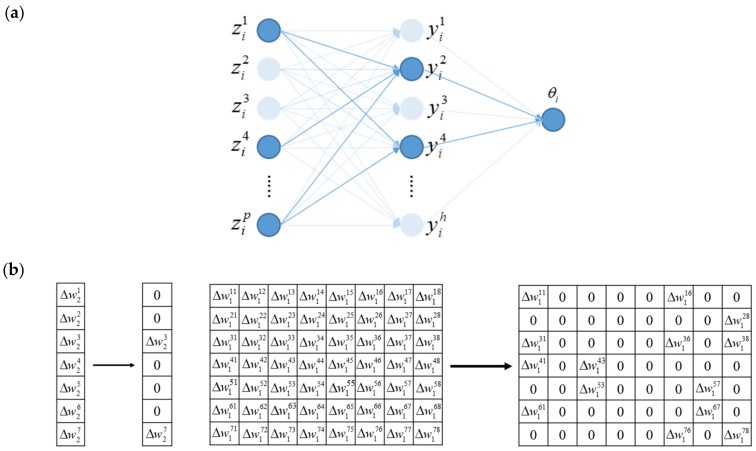
(**a**) Structure diagram of the proposed NN with TGDR estimation. (**b**) Gradient matrix under thresholding.

First developed for linear regression [3] and later extended to many other regression models [30,31,35], TGDR is a generically applicable regularized optimization technique. The key strategy is that, in gradient-based optimization, in each iteration, gradients are compared and important parameters with large gradients identified. Then, estimates are updated only for those important parameters. Thresholding and variable selection are conducted in each iteration, and hence the final estimates can have sparsity and regularization. Building on the NN structure shown in Figure A1 (Appendix A), in the upper panel of Figure 1, we schematically present the TGDR-based estimation, where the light dotted lines indicate that the connection parameters are zeros, and the light-colored nodes have no effect on the output.

Denote $z_{i}=\left(z_{i}^{1},z_{i}^{2},\ldots ,z_{i}^{p}\right)$. Further, let 0≤τ≤1 denote the threshold, which is a tuning parameter. The TGDR-based algorithm proceeds as follows:

(1) Denote *h* as the number of hidden layer nodes. Initialize *k* = 0, $W_{1}^{h\times p}=0$,$\;W_{2}^{1\times h}=0$,$\;b\sim N\;\left(0,0.01I\right)$, learning rate *α* = 0.01, and learning rate decay *η* = 0.99.
(2) Compute $\theta_{i}=W_{2}\sigma \left(W_{1}z_{i}\prime +b\right)$, $l\left(W_{1},W_{2},b\right)=\sum_{\delta_{i}=1}\left(\theta_{i}-\log\sum_{t_{j}>t_{i}}\exp(\theta_{j})\right)$.
(3) Update *k* = *k* + 1. Compute the negative gradients $\Delta W_{2}=-\frac{\partial l\left(W_{2}\right)}{\partial W_{2}}$,$\;\Delta W_{1}=-\frac{\partial l\left(W_{1}\right)}{\partial W_{1}}$ and $\Delta b=-\frac{\partial l\left(b\right)}{\partial b}$. Denote the *j*th component of vector $\Delta W_{2}$ as $\Delta W_{2}^{j}$, and the *j*th row of matrix $\Delta W_{1}$ as $\Delta W_{1}^{j\cdot }$.
(4) Compute the thresholding indicators $L_{2}=\left(L_{2}^{1},L_{2}^{2},\ldots ,L_{2}^{h}\right)$ and $L_{1}=\left(L_{1}^{1\cdot },L_{1}^{2\cdot },\ldots ,L_{1}^{h\cdot }\right)\prime$: $L_{2}^{j}=I(|\Delta W_{2}^{j}\left|\geq \tau \max|\Delta W_{2}\right|)$, $L_{1}^{jl}=I(\left|\Delta W_{1}^{jl}\right|\geq \tau \max|\Delta W_{1}^{j\cdot }|)$, where l=1, 2,…,p.
(5) Update $W_{2}\leftarrow W_{2}+\alpha L_{2}⊗\Delta W_{2}$, $W_{1}\leftarrow W_{1}+\alpha L_{1}⊗\Delta W_{1}$, and b←b+αΔb. The product of L and ΔW is component-wise.
(6) Repeat Steps (2)–(5) K times, where K is another tuning parameter.

In Step (1), the initialization of the NN parameters is different from other networks. Here, parameters $W_{1}$ and $W_{2}$ need to be initialized as zero, which ensures sparsity in the subsequent optimization. Simultaneously, *b* is initialized as a random vector with components close to zero to facilitate activating all hidden layer nodes. In Step (2), we carry out forward propagation of the NN. The activation function in the hidden layer can increase the ability of the network to solve nonlinear problems and prevent gradient explosion. In Step (3), the gradients of the connection parameter matrix are calculated. In Step (4), we set the threshold according to the maximum value of the gradients between the hidden layer and the output layer—this is similar to the operation in regression. In addition, the threshold of gradients between the input layer and the hidden layer is determined by the maximum value of the gradients corresponding to each hidden layer. In Step (5), the parameters are updated based on the threshold values generated in Step (4)—only the important ones are updated. When a parameter’s gradient remains below the threshold in all iterations, its final estimate will be exactly zero, thus achieving sparsity and regularization. In the lower panel of Figure 1, we show a small example with p=8, h=7. On the left are the gradients between the hidden layer and the output layer, and on the right are the gradients between the input layer and the hidden layer. With the threshold, only a small number of parameters are updated. We note that sparsity here is more “transparent” than penalization and some other regularization approaches.

The final network structure is jointly determined by tuning parameters τ and K. A smaller value of τ and a larger value of K lead to denser estimates. In regression, it has been recognized that τ≈0 leads to ridge-type estimation and τ≈1 leads to lasso-type estimation. In numerical studies, we select τ and K via cross-validation. When it is desirable to further reduce computational cost for very high-dimensional data, τ can be fixed at a large value close to 1. Here, it is noted that parameters $W_{1}$ and $W_{2}$ in the model cannot be used for variable selection directly. We obtain a new vector $W_{12}$ by matrix multiplication of $W_{1}$ and $W_{2}$, and then select variables correspondingly.

As in each iteration, the gradient matrix is sparse and the proposed approach can be computationally less demanding than some of the existing ones. To further fix ideas, we consider a simulated dataset with sample size 2000, dimension of input 1000, number of hidden layer nodes 10, and number of iterations 200. With a “standard” configuration (CPU: Intel core i9-8950HK, RAM 32.0 GB, GPU NVIDIA GeForce GTX 1080), the optimization of the proposed NN takes 6.50 s, about 14% more efficient than its key competitor. Additional comparisons are presented in Table A1 in Appendix A. Computational cost is expected to depend on sample size, number of nodes, and other parameters in a similar way as for other NNs.

To facilitate data analysis, the computer code implementing the proposed method is publicly available at: https://github.com/shuanggema/CNTsurv, accessed on 17 August 2022.

## 3. Simulation

We conduct simulation to better comprehend the effectiveness of the proposed method. Here, it is noted that many NN studies only analyze real data without simulation study. Simulated data, although they may be somewhat simplified, can be advantageous with known generating mechanisms, which facilitates an objective evaluation and comparison. To better gauge its performance, we compare the proposed method against: (a) Cox-nnet with lasso (CNL), which is an NN approach based on the same Cox model-based loss function. Lasso, as a representative of penalization, is applied for regularization and sparsity. Similar methods have been adopted in the literature [21,22]: (b) Cox regression with TGDR (CT), which conducts Cox regression analysis and applies TGDR for regularization and sparsity; (c) Cox regression with lasso (CPH), which conducts Cox regression analysis and applies lasso for regularization and sparsity; and (d) random survival forest (RSF), which applies the random forest technique to survival analysis and has competitive performance. For abbreviation, we refer to the proposed method as CNT, where T stresses TGDR. We recognize that many approaches can be applied to analyze the simulated data. The above four can be the most relevant. Specifically, comparing with CNL can directly establish the advantage of TGDR, and comparing with CT can directly establish the advantage of NN. CPH and RSF represent popular and competitive existing approaches.

We compare the proposed and alternative methods in terms of prediction and variable-selection performance. In prediction evaluation, we first generate the training data and build models using the proposed and alternative methods. Then, a testing dataset is generated under the same settings. We make predictions for the testing-set samples using the training-data models and evaluate prediction performance using C-index. This measure ranges between 0 and 1, with a larger value indicating better prediction. When evaluating variable-selection performance, we adopt the popular strategy with which a variable is identified as important if it has a nonzero effect—we again refer to Figure 1 for a schematic presentation. Consider the true positive (TP), false positive (FP), true negative (TN), and false negative (FN) of variable selection under the selected tuning parameter values. We first calculate $P=\frac{\mathrm{TP}}{\mathrm{TP}+\mathrm{FP}},\;R=\frac{\mathrm{TP}}{\mathrm{TP}+\mathrm{FN}}$. The first variable-selection accuracy measure is F1, defined as $F=\frac{\left(\alpha^{2}+1\right)P\cdot R}{\alpha^{2}\left(P+R\right)}$, where α=1. Here, a larger value indicates more accurate selection. Additionally, to get a “global picture” of variable selection, we follow the literature [36], consider a sequence of tuning parameter values, evaluate variable-selection performance (TP and FP) at each tuning parameter value, and generate the receiver-operating characteristic (ROC) curve and calculate the area under the curve (AUC) for evaluation. AUC ranges between 0.5 and 1, with a larger value indicating higher accuracy.

When implementing CNL and CNT, for the hyperparameters, we consider: one hidden layer (noting that both approaches can be directly extended to multiple hidden layers), 10 nodes in the hidden layer, tanh activation function, initial learning rate *α* = 0.01, and learning rate decay *η* = 0.99. Cross-validation is applied to select the threshold and number of iterations for CNT and CT, the penalization parameter for CNL and CPH, and the number of forests for RSF.

For NN methods in general, the number of hidden layers may have an impact on model performance. An increase in the number of hidden layers means increased network complexity and computational cost. Usually, it is recommended that, when sample size is sufficiently large and signals are dense, multiple hidden layers are needed to achieve sufficient model complexity. For the proposed settings with a limited sample size and sparse signals, one hidden layer may be preferred. In Table A2 (Appendix A), we consider Simulation 1 and Scenario 4 (details described below) and 1–3 hidden layers. It is observed that one hidden layer may be sufficient—here, we do note that more hidden layers may be needed for other data settings.

We comprehensively consider the following simulation settings.

**Simulation setting 1:** Here, we further consider four scenarios with different conditional hazard functions.

Scenario 1: $\lambda (t|z_{i})=\lambda_{0}\left(t\right)\exp\left(\sum_{j=1}^{up}z_{i}^{j}\right)$,

Scenario 2: $\lambda (t|z_{i})=\lambda_{0}\left(t\right)\exp\left(\sum_{j=1}^{up}z_{i}^{j}+{\left(z_{i}^{up+1}\right)}^{2}+{\left(z_{i}^{up+2}\right)}^{2}\right)$,

Scenario 3: $\lambda (t|z_{i})=\lambda_{0}\left(t\right)\exp\left(\sum_{j=1}^{up}z_{i}^{j}+5*\left(z_{i}^{up}z_{i}^{up-1}+z_{i}^{up-1}z_{i}^{up-2}\right)\right)$,

Scenario 4: $\lambda (t|z_{i})=\lambda_{0}\left(t\right)\exp\left(\sum_{j=1}^{up}z_{i}^{j}+{\left(z_{i}^{up+1}\right)}^{2}+{\left(z_{i}^{up+2}\right)}^{2}+5*\left(z_{i}^{up}z_{i}^{up-1}+z_{i}^{up-1}z_{i}^{up-2}\right)\right)$, where *up* is the number of variables that have linear effects on survival. For $z_{i}=\left(z_{i}^{1},\;z_{i}^{2},\ldots ,z_{i}^{p}\right)$, we generate from two multivariate normal distributions, namely, $MVN\;\left(0,\Sigma_{1}\right)$ and $MVN\;\left(0,\Sigma_{2}\right)$, where $\Sigma_{1}=\left\{\Sigma_{lk}=e^{-\left|l-k\right|},1\leq l,k\leq p\right\}$ and $\Sigma_{2}=I_{p}$. For the baseline hazard function $\lambda_{0}\left(t\right)$, we set $\lambda_{0}\left(t\right)=sr^{s}t^{s-1}$ with r=0.1, s=2. The censoring times are independently generated from an exponential distribution, and the distribution parameter is adjusted to achieve a target censoring rate of 30%. The number of variables is *p* = 50, 100, 200, 500, 1000, and for each *p*, the number of samples *N* = 500, 1000, 2000, 5000.

**Simulation setting 2:** We also consider four scenarios with different conditional hazard functions.

Scenario 1: $\lambda (t|z_{i})=\lambda_{0}\left(t\right)\exp\left(\sum_{j=1}^{\left[\frac{up}{2}\right]}\beta_{1}z_{i}^{j}+\sum_{j=\left[\frac{up}{2}\right]+1}^{up}\beta_{2}z_{i}^{j}\right)$,

Scenario 2: $\lambda (t|z_{i})=\lambda_{0}\left(t\right)\exp\left(\sum_{j=1}^{\left[\frac{up}{2}\right]}\beta_{1}z_{i}^{j}+\sum_{j=\left[\frac{up}{2}\right]+1}^{up}\beta_{2}z_{i}^{j}+{\left(z_{i}^{up+1}\right)}^{2}+{\left(z_{i}^{up+2}\right)}^{2}\right)$,

Scenario 3: $\lambda (t|z_{i})=\lambda_{0}\left(t\right)\exp\left(\sum_{j=1}^{\left[\frac{up}{2}\right]}\beta_{1}z_{i}^{j}+\sum_{j=\left[\frac{up}{2}\right]+1}^{up}\beta_{2}z_{i}^{j}+5*\left(z_{i}^{up}z_{i}^{up-1}+z_{i}^{up-1}z_{i}^{up-2}\right)\right)$,

Scenario 4: $\lambda (t|z_{i})=\lambda_{0}\left(t\right)\exp\left(\sum_{j=1}^{\left[\frac{up}{2}\right]}\beta_{1}z_{i}^{j}+\sum_{j=\left[\frac{up}{2}\right]+1}^{up}\beta_{2}z_{i}^{j}+{\left(z_{i}^{up+1}\right)}^{2}+{\left(z_{i}^{up+2}\right)}^{2}+5*\left(z_{i}^{up}z_{i}^{up-1}+z_{i}^{up-1}z_{i}^{up-2}\right)\right)$, where $\beta_{1}=1$ and $\beta_{2}=0.3$. $z_{i}$s are generated in the same manner as above. The baseline hazard function and censoring time distribution are specified in the same way as above. The target censoring rate is the same as above. We also consider *p* = 50, 100, 200, 500, 1000 and *N* = 500, 1000, 2000, 5000.

**Simulation setting 3:** Here, we consider the conditional hazard function:

$\lambda (t|z_{i})=\lambda_{0}\left(t\right)\exp\left(\sum_{j=1}^{up}z_{i}^{j}+{\left(z_{i}^{up+1}\right)}^{2}+{\left(z_{i}^{up+2}\right)}^{2}+5*\left(z_{i}^{up-2}z_{i}^{up-1}+z_{i}^{up-1}z_{i}^{up}\right)\right)$, and the other simulation settings are similar to above. We consider *p* = 500, *up* = 5, 10, 15, 20, 30, and *N* = 500, 1000, 2000, 5000.

Under Simulation 1, all linear effects have “equal effects” with regression coefficients 1. Under Simulation 2, half of the linear effects have relatively strong signals with regression coefficients 1, and the other half have relatively weak signals with regression coefficients 0.3. Under Simulations 1 and 2, Scenario 1 contains only linear effects, which favors the CT and CPH approaches. Scenario 2 contains linear and quadratic effects. Scenario 3 contains linear effects and interactions. Scenario 4 contains linear and quadratic effects, as well as interactions. Scenarios 2–4 have been designed to demonstrate the NN’s flexibility in accommodating unspecified nonlinear effects. For these simulation settings, we have considered multiple values of data dimensionality and sample size. The relatively low-dimensional settings (for example, with *p* = 50) can shed insights into performance of the proposed approach with “regular” survival data. We note that the dimensionality is not ultrahigh. In practical data analysis, to improve stability, screening is often conducted with ultrahigh-dimensional omics measurements to reduce dimensionality to a more manageable level. This may be especially needed for NNs, which have significantly more parameters and hence lower stability. In Simulations 1 and 2, the number of linear effects is fixed. In addition, the number of quadratic terms and that of interactions are fixed at 2. Further, in Simulation 3, we fix the total data dimensionality, as well as the number of quadratic terms and that of interactions, and vary the number of linear effects.

**Figure A2:**
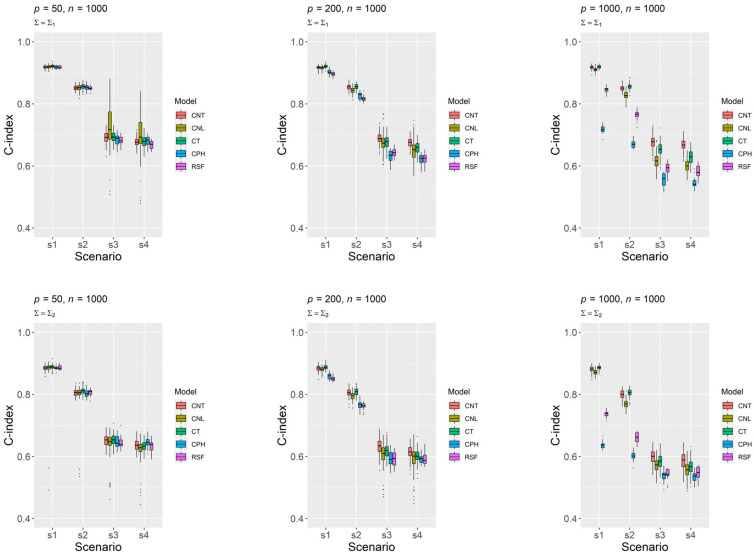
Simulation 1: box plot of C-index.

**Figure A3:**
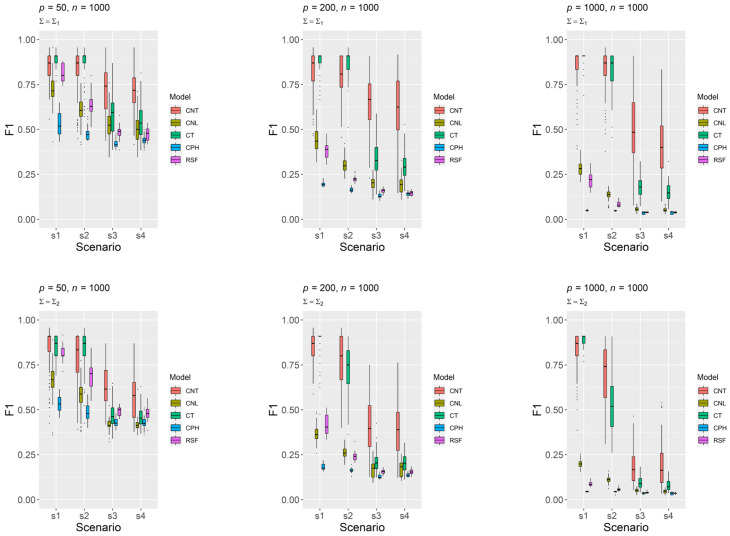
Simulation 1: box plot of F1.

**Figure A4:**
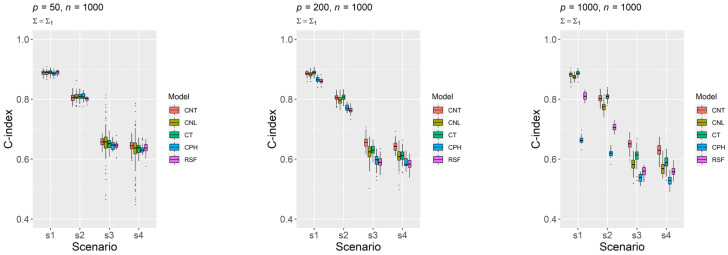

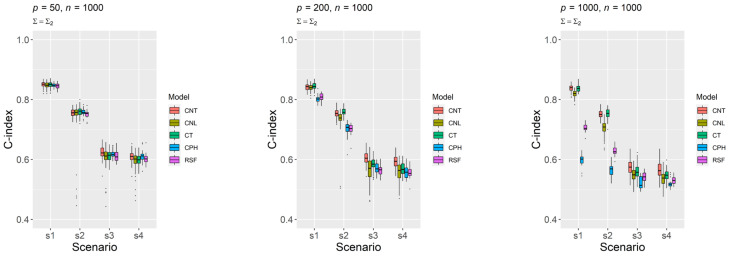
Simulation 2: box plot of C-index.

**Figure A5:**
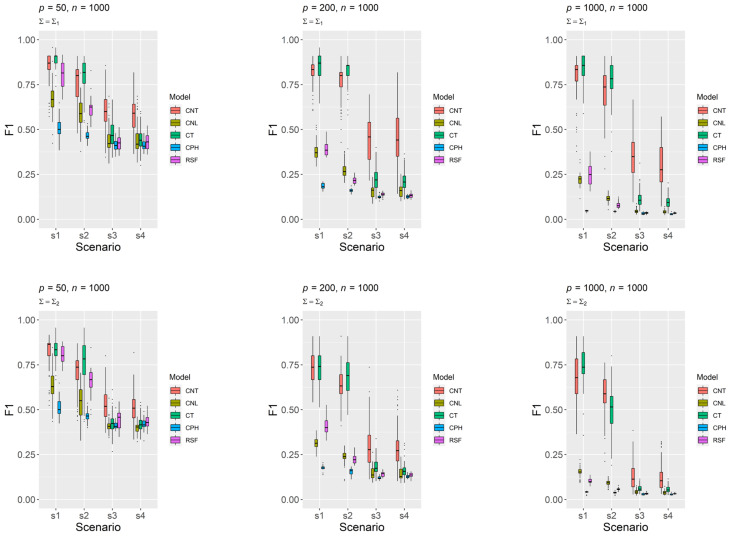
Simulation 2: box plot of F1.

**Figure A6:**
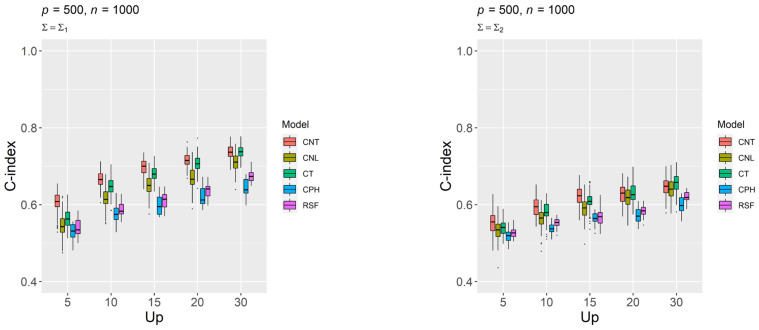
Simulation 3: box plot of C-index.

**Figure A7:**
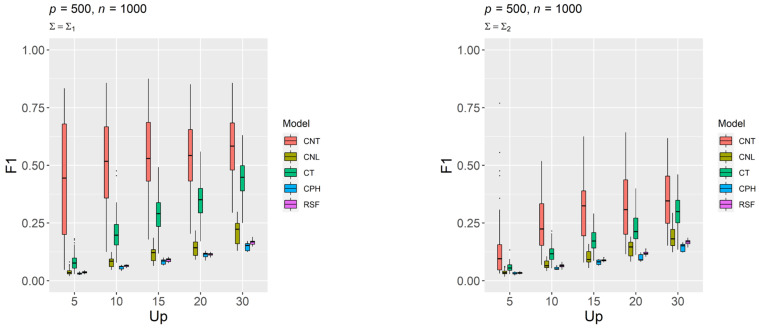
Simulation 3: box plot of F1.

The results based on 200 replicates are numerically summarized in Table A3, Table A4, Table A5, Table A6, Table A7, Table A8, Table A9, Table A10, Table A11, Table A12, Table A13, Table A14, Table A15, Table A16, Table A17, Table A18, Table A19 and Table A20 (Appendix A) and graphically summarized in Figure A2, Figure A3, Figure A4, Figure A5, Figure A6 and Figure A7 (Appendix A). It is observed that the proposed method has competitive performance—it outperforms the four alternatives under most of the settings. When sample size increases, in general, the performance of all methods improves (sometimes significantly). When dimensionality increases, performance may deteriorate; however, in general not significantly. Under simpler settings, CT may excel—similar observations have been made in the literature [22]. However, the proposed method still has competitive performance. Consider, for example, Simulation 1, Scenario 1, *p* = 1000, *N* = 1000, and the first type of correlation structure. The five methods have average C-index values 0.917 (CNT), 0.91 (CNL), 0.919 (CT), 0.718 (CPH), and 0.845 (RSF), respectively, and the average F1 values are 0.895 (CNT), 0.284 (CNL), 0.995 (CT), 0.044 (CPH), and 0.033 (RSF), respectively. As data become more complicated, advantages of the proposed method get more obvious. Consider, for example, Scenario 4 and otherwise the same settings as above. The average C-index values are 0.667 (CNT), 0.601 (CNL), 0.629 (CT), 0.546 (CPH), and 0.581 (RSF), respectively, and the average F1 values are 0.402 (CNT), 0.053 (CNL), 0.15 (CT), 0.036 (CPH), and 0.027 (RSF), respectively.

## 4. Data Analysis

We further test the proposed method using a gene expression dataset obtained from GEO and a DNA methylation dataset obtained from TCGA.

### 4.1. High-Grade Serous Ovarian Cancer Data

The first dataset is on high grade serous ovarian cancer (HGSOC) and obtained from GEO (GSE132342; https://www.ncbi.nlm.nih.gov/geo/query/acc.cgi?acc=GSE132342, accessed on 17 May 2022). This dataset contains records on 3769 HGSOC patients, and 513 gene expression measurements are available for each patient. Among the 3769 patients, 2475 died during follow-up, and the survival times ranged from 0.03 to 117.67 months, with a median of 33.35 months. The rest were right-censored, and the follow-up times ranged from 0.03 to 117.74 months, with a median of 69.61 months. Additional sample characteristics are described in Table A21 (Appendix A).

When implementing the proposed and alternative NN methods, we consider the following hyperparameter specifications: one hidden layer, 20 nodes in the hidden layer, tanh activation function, initial learning rate *α* = 0.01, and learning rate decay *η* = 0.99. For all the methods, the other tuning parameters are selected using cross-validation. The numbers of identified variables are 99 (CNT), 132 (CNL), 26 (CT), 54 (CPH), and 73 (RSF). It is observed that different methods lead to different identification results. More details are available from the authors.

**Figure 2:**
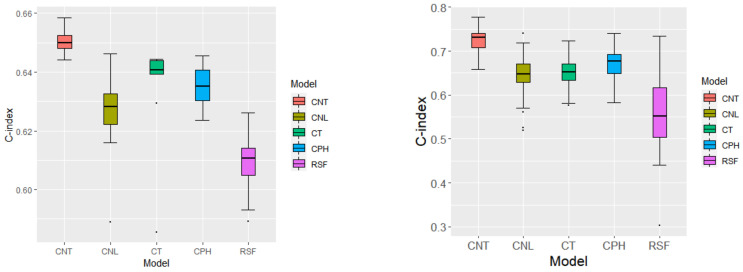

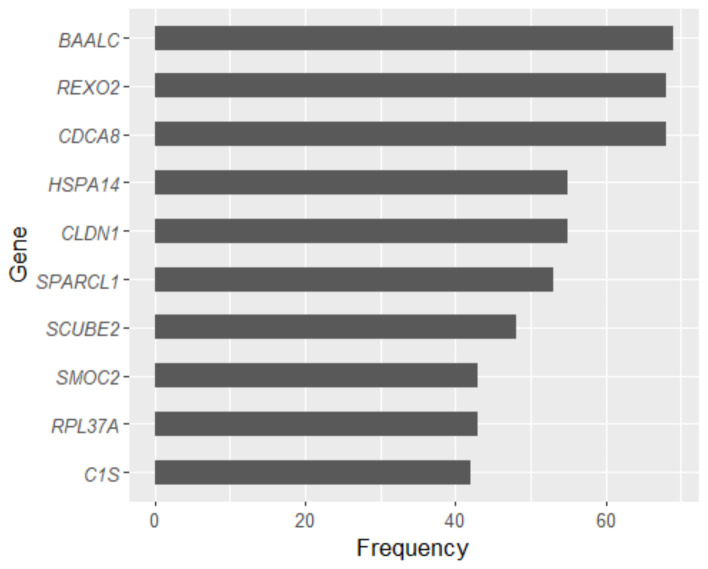
Data analysis. Upper: box plot of C-index from 100 random splittings; lower: top ten methylation loci with the highest identification frequency. Left: HGSOC data; right: BRCA data.

With practical data, there is a lack of an objective way of assessing variable identification performance. We resort to a random splitting approach, which has been popularly adopted in the literature [37], to evaluate prediction performance and stability of selection (which can provide “indirect” support to the validity of modeling). Specifically, we randomly split data into a training and a testing set with equal sizes. Here, as the effective sample size (number of events) is limited, we choose a relatively larger testing data size to ensure the stability of evaluation. Models are constructed using the training data, and prediction is made for samples in the testing data. With each random splitting, we can obtain the C-index value from the testing data and a set of identified genes. With 100 random splittings, the proposed approach has a mean C-index of 0.650 with SD = 0.003 compared to 0.627 (SD 0.011) for CNL, 0.638 (SD 0.012) for CT, 0.634 (SD 0.007) for CPH, and 0.610 (SD 0.007) for RSF. In Figure 2, we show the box plots of the C-index values. In the stability evaluation, the same 99 genes (Table A22, Appendix A) are identified in all the random splittings, which suggests a high level of stability. Stability information on the alternatives, which is not as satisfactory as the proposed approach, is available from the authors.

Among these 99 genes, many have been previously suggested as relevant for ovarian cancer, suggesting the “biological validity” of our analysis. For example, studies have suggested that TAP1 is involved in the antigen-presenting pathway and positively associated with ovarian cancer survival [38]. Additionally, hypomethylation of TAP1 is associated with the prolongation of disease-recurrence time. ZFHX4 may play a role in neural and muscle differentiation and be involved in transcriptional regulation. CXCL9 is associated with higher lymphocytic infiltration, which is a characteristic of the immunoreactive HGSOC molecular subtype. FBN1 is an extracellular matrix protein. As a biomarker of early recurrence in patients with ovarian cancer, it is closely related to connective tissue proliferation in HGSOC. TGER3 has been suggested as associated with relapse-free survival of HGSOC. DH1B belongs to a pathway that promotes ovarian cancer cell infiltration [39]. C10orf82 has been previously identified as associated with ovarian cancer overall survival [40]. The overexpression of COL11A1 has been found to be associated with the progression of ovarian cancer [41,42]. In terms of mechanism, it confers chemoresistance on ovarian cancer cells through the activation of the Akt/c/EBPβ pathway [43]. CXCL10 has a tumor-suppressive function by TIL recruitment in ovarian cancer [44]. FABP4 is a key factor of ovarian cancer and has strong metastatic potential [45]. LGALS4 has been suggested as an early diagnostic marker of mucinous ovarian cancer [46]. LBP has been validated as a diagnostic biomarker of ovarian cancer [47]. Many other genes also have strong implications, and we omit their discussions here.

### 4.2. Breast Invasive Carcinoma Data

The second dataset is on breast cancer with DNA methylation measurements and obtained from TCGA (https://portal.gdc.cancer.gov/projects/TCGA-BRCA, accessed on 6 September 2022). The pipeline for processing TCGA DNA methylation data has been described in the literature [48]. The analyzed data contain records on 1093 breast cancer patients. For methylation loci, we take a “candidate gene” approach and focus on the 63 genes that are included in three gene-testing platforms (Oncotype DX [49], MammaPrint [50], and EndoPredict [51]). Among the 1093 patients, 152 died during follow-up, and the survival times ranged from 0 to 248.5 months, with a median of 38.23 months. For the 941 censored patients, the follow-up times ranged from 0 to 286.83 months, with a median of 25.33 months. Additional sample characteristics are described in Table A23 (Appendix A).

Data are analyzed using the proposed and four alternative methods in the same way as above. The only difference is that, with a lower dimensionality, the number of nodes in the hidden layer is set as 10. Detailed estimation results are available from the authors. In the evaluation of prediction, the C-index values based on 100 random splittings are 0.725 (0.024) for the proposed method, 0.646 (0.039) for CNL, 0.652 (0.03) for CT, 0.671 (0.036) for CPH, and 0.562 (0.074) for RSF. The box plots are shown in Figure 2. Also in Figure 2, we show the top ten genes with the highest stability. Among them, genes BAALC, REXO2, CDCA8 are selected in over 60 splittings, BAALC, REXO2, CDCA8, HSPA14, CLDN1, SPARCL1, SMOC2, and C1S are from MammaPrint, SCUBE2 is from Oncotype DX, and RPL37A is from EndoPredict. The alternative approaches have less satisfactory stability (details omitted here).

Among the identified genes, BAALC is a protein-coding gene and associated with acute leukemia and other cancers. REXO2 may play a role in DNA repair, replication, and recombination, as well as RNA processing and degradation. It may also participate in the resistance of human cells to UVC-induced cell death through its role in the process of DNA repair. CDCA8 encodes a component of the chromosome passenger complex. This complex is an important regulator of mitosis and cell division. Its encoded protein is regulated by cell cycle and required for chromatin-induced microtubule stabilization and spindle formation. HSPA14 has multiple functions, including ATP, misfolded protein, and unfolded protein bindings. SCUBE2 enables calcium ion binding activities and is involved in signal transduction. It may also act upstream of or within several processes, including positive regulation of chondrocyte proliferation. RPL37A encodes a ribosomal protein and is a component of the 60s subunit. This protein belongs to the l37ae family of ribosomal proteins.

## 5. Discussion

In this study, we have developed a new NN-based modeling strategy for data with a censored survival response and high-dimensional omics measurements. A new regularized estimation and selection method has been developed based on the TGDR technique. Considering the superiority of the NN over regression, relatively limited developments in the NN for censored survival data and high-dimensional covariates and satisfactory performance of TGDR in regression, the proposed methodological development is warranted. The proposed method has an intuitive formulation, computational efficiency, and satisfactory performance in simulation. Here, it is noted that the proposed method is not dominatingly better in simulation, which is as expected. Its superiority becomes more prominent with the increasing complexity of data. Its effectiveness has been further demonstrated using two cancer studies.

This study can be potentially extended in multiple ways. In our numerical studies, a single hidden layer has been adopted, and led to satisfactory results. Our limited exploration has suggested that, for our data settings, additional hidden layers may not be needed—however, this may not be true for other data. When determining the number of hidden layer nodes, we have taken sample size and number of input variables into consideration. Our literature search has not suggested an objective criterion for determining node number. Searching over a few candidate values and selecting the optimal (for example, with cross-validation) may be needed. Additionally, following the extension of TGDR to other regression settings, we can also develop TGDR-based NNs for other data/model settings. Last, but not least, more data analysis using the proposed method may be warranted in future research.

## Data Availability

Publicly available datasets were analyzed in this study. The HGSOC dataset can be found here: https://www.ncbi.nlm.nih.gov/geo/query/acc.cgi?acc=GSE132342, accessed on 17 May 2022, and the BRCA dataset can be found here: https://portal.gdc.cancer.gov/repository, accessed on 6 September 2022.

## Appendix A

**Table A1:** Average computer time for the analysis of one simulation replicate under Simulation 1, Scenario 4.

		Computer Time (s)	
		CNT	CNL
*p* = 200	*N* = 500	2.56	2.74
	*N* = 1000	3.46	4.22
	*N* = 2000	6.32	7.22
	*N* = 5000	14.34	16.18
*p* = 500	*N* = 500	2.62	2.78
	*N* = 1000	3.42	4.22
	*N* = 2000	6.36	7.08
	*N* = 5000	14.16	16.16
*p* = 1000	*N* = 500	2.5	2.82
	*N* = 1000	3.64	4.24
	*N* = 2000	6.5	7.44
	*N* = 5000	14.28	16.30

**Table A2:** Mean (SD) of C-index for the CNT method with different numbers of hidden layers under Simulation 1, Scenario 4. The bold numbers represent the optimal results.

			Number of Hidden Layers		
			1	2	3
**Σ_1_**	*p* = 50	*N* = 500	**0.664 (0.028)**	0.656 (0.027)	0.629 (0.039)
		*N* = 1000	**0.677 (0.014)**	0.675 (0.017)	0.649 (0.025)
		*N* = 2000	**0.686 (0.012)**	0.681 (0.013)	0.668 (0.038)
		*N* = 5000	0.69 (0.008)	0.689 (0.01)	**0.82 (0.091)**
	*p* = 100	*N* = 500	**0.657 (0.021)**	0.654 (0.028)	0.6 (0.031)
		*N* = 1000	**0.676 (0.018)**	0.665 (0.019)	0.625 (0.047)
		*N* = 2000	**0.684 (0.013)**	0.677 (0.014)	0.66 (0.015)
		*N* = 5000	0.691 (0.008)	0.686 (0.006)	**0.787 (0.092)**
	*p* = 200	*N* = 500	0.647 (0.027)	**0.656 (0.03)**	0.575 (0.043)
		*N* = 1000	**0.675 (0.016)**	0.662 (0.017)	0.58 (0.037)
		*N* = 2000	**0.684 (0.013)**	0.677 (0.012)	0.618 (0.063)
		*N* = 5000	**0.691 (0.008)**	0.684 (0.008)	0.649 (0.066)
	*p* = 500	*N* = 500	0.635 (0.03)	**0.636 (0.029)**	0.539 (0.029)
		*N* = 1000	**0.666 (0.021)**	0.665 (0.02)	0.582 (0.036)
		*N* = 2000	**0.681 (0.011)**	0.681 (0.012)	0.596 (0.038)
		*N* = 5000	**0.69 (0.007)**	0.686 (0.008)	0.61 (0.055)
	*p* = 1000	*N* = 500	**0.625 (0.033)**	0.624 (0.036)	0.544 (0.031)
		*N* = 1000	**0.667 (0.017)**	0.653 (0.033)	0.54 (0.032)
		*N* = 2000	**0.68 (0.011)**	0.673 (0.01)	0.567 (0.047)
		*N* = 5000	**0.689 (0.008)**	0.686 (0.009)	0.615 (0.013)

**Table A3:** Simulation 1, Scenario 1: mean (SD) of C-index.

			CNT	CNL	CT	CPH	RSF
**Σ_1_**	*p* = 50	*N* = 500	0.914 (0.009)	0.916 (0.008)	**0.92 (0.008)**	0.911 (0.008)	0.909 (0.008)
		*N* = 1000	0.918 (0.005)	0.919 (0.005)	**0.921 (0.005)**	0.918 (0.005)	0.917 (0.005)
		*N* = 2000	0.92 (0.003)	0.921 (0.003)	**0.921 (0.004)**	0.92 (0.003)	0.919 (0.003)
		*N* = 5000	0.921 (0.002)	**0.922 (0.002)**	0.921 (0.002)	0.922 (0.002)	0.921 (0.002)
	*p* = 100	*N* = 500	0.909 (0.009)	0.909 (0.009)	**0.917 (0.008)**	0.9 (0.009)	0.896 (0.011)
		*N* = 1000	0.917 (0.006)	0.917 (0.006)	**0.92 (0.005)**	0.914 (0.007)	0.912 (0.006)
		*N* = 2000	0.92 (0.004)	0.921 (0.004)	**0.921 (0.004)**	0.92 (0.004)	0.916 (0.003)
		*N* = 5000	0.921 (0.002)	0.921 (0.002)	**0.921 (0.002)**	0.92 (0.002)	0.919 (0.003)
	*p* = 200	*N* = 500	0.91 (0.01)	0.907 (0.01)	**0.917 (0.008)**	0.827 (0.032)	0.871 (0.011)
		*N* = 1000	0.916 (0.006)	0.916 (0.006)	**0.921 (0.005)**	0.902 (0.008)	0.896 (0.007)
		*N* = 2000	0.919 (0.004)	0.92 (0.004)	**0.921 (0.004)**	0.915 (0.004)	0.911 (0.005)
		*N* = 5000	0.921 (0.002)	0.921 (0.002)	**0.921 (0.002)**	0.92 (0.002)	0.917 (0.002)
	*p* = 500	*N* = 500	0.907 (0.013)	0.902 (0.01)	**0.917 (0.008)**	0.68 (0.034)	0.838 (0.012)
		*N* = 1000	0.917 (0.007)	0.911 (0.007)	**0.92 (0.005)**	0.829 (0.011)	0.861 (0.011)
		*N* = 2000	0.919 (0.004)	0.917 (0.003)	**0.921 (0.004)**	0.903 (0.004)	0.89 (0.005)
		*N* = 5000	0.921 (0.003)	0.921 (0.002)	**0.921 (0.002)**	0.917 (0.003)	0.911 (0.003)
	*p* = 1000	*N* = 500	0.907 (0.014)	0.905 (0.009)	**0.915 (0.009)**	0.663 (0.016)	0.823 (0.012)
		*N* = 1000	0.917 (0.006)	0.91 (0.006)	**0.919 (0.005)**	0.718 (0.013)	0.845 (0.009)
		*N* = 2000	0.92 (0.004)	0.917 (0.004)	**0.921 (0.004)**	0.873 (0.008)	0.862 (0.008)
		*N* = 5000	0.921 (0.002)	0.92 (0.002)	**0.921 (0.002)**	0.913 (0.003)	0.897 (0.003)
**Σ_2_**	*p* = 50	*N* = 500	0.872 (0.013)	0.87 (0.055)	**0.883 (0.011)**	0.875 (0.009)	0.875 (0.01)
		*N* = 1000	0.884 (0.009)	0.879 (0.051)	**0.888 (0.007)**	0.885 (0.006)	0.886 (0.008)
		*N* = 2000	0.888 (0.005)	0.885 (0.034)	**0.889 (0.005)**	0.888 (0.005)	0.888 (0.005)
		*N* = 5000	0.89 (0.003)	0.886 (0.037)	**0.89 (0.003)**	0.89 (0.003)	0.89 (0.003)
	*p* = 100	*N* = 500	0.865 (0.018)	0.872 (0.013)	**0.881 (0.012)**	0.853 (0.018)	0.85 (0.013)
		*N* = 1000	0.883 (0.009)	0.884 (0.008)	**0.887 (0.007)**	0.876 (0.008)	0.873 (0.006)
		*N* = 2000	0.888 (0.005)	0.885 (0.032)	**0.89 (0.005)**	0.887 (0.006)	0.884 (0.005)
		*N* = 5000	0.89 (0.003)	0.886 (0.038)	**0.89 (0.003)**	0.889 (0.002)	0.887 (0.004)
	*p* = 200	*N* = 500	0.859 (0.022)	0.856 (0.016)	**0.88 (0.013)**	0.722 (0.047)	0.8 (0.019)
		*N* = 1000	0.883 (0.009)	0.88 (0.008)	**0.887 (0.007)**	0.859 (0.011)	0.85 (0.007)
		*N* = 2000	0.888 (0.005)	0.883 (0.038)	**0.889 (0.005)**	0.879 (0.005)	0.874 (0.004)
		*N* = 5000	0.89 (0.003)	0.882 (0.054)	**0.89 (0.003)**	0.888 (0.003)	0.884 (0.004)
	*p* = 500	*N* = 500	0.853 (0.029)	0.846 (0.014)	**0.879 (0.013)**	0.603 (0.041)	0.733 (0.02)
		*N* = 1000	0.883 (0.008)	0.873 (0.009)	**0.888 (0.008)**	0.736 (0.016)	0.774 (0.013)
		*N* = 2000	0.888 (0.005)	0.885 (0.005)	**0.89 (0.005)**	0.856 (0.009)	0.838 (0.009)
		*N* = 5000	0.889 (0.003)	0.886 (0.038)	**0.89 (0.003)**	0.884 (0.003)	0.875 (0.004)
	*p* = 1000	*N* = 500	0.842 (0.032)	0.851 (0.018)	**0.876 (0.013)**	0.601 (0.038)	0.714 (0.021)
		*N* = 1000	0.881 (0.009)	0.871 (0.008)	**0.886 (0.007)**	0.637 (0.011)	0.737 (0.01)
		*N* = 2000	0.887 (0.005)	0.881 (0.006)	**0.889 (0.005)**	0.798 (0.01)	0.784 (0.009)
		*N* = 5000	0.889 (0.003)	0.888 (0.003)	**0.89 (0.003)**	0.876 (0.003)	0.851 (0.004)

**Table A4:** Simulation 1, Scenario 2: mean (SD) of C-index.

			CNT	CNL	CT	CPH	RSF
**Σ_1_**	*p* = 50	*N* = 500	0.843 (0.017)	0.846 (0.015)	**0.852 (0.014)**	0.834 (0.014)	0.841 (0.011)
		*N* = 1000	0.851 (0.008)	0.852 (0.01)	**0.856 (0.009)**	0.853 (0.01)	0.85 (0.007)
		*N* = 2000	0.857 (0.007)	0.858 (0.006)	**0.857 (0.006)**	0.856 (0.006)	0.854 (0.006)
		*N* = 5000	0.857 (0.004)	**0.866 (0.014)**	0.858 (0.004)	0.859 (0.004)	0.858 (0.004)
	*p* = 100	*N* = 500	0.843 (0.015)	0.838 (0.016)	**0.853 (0.014)**	0.812 (0.018)	0.817 (0.015)
		*N* = 1000	0.853 (0.01)	0.852 (0.009)	**0.855 (0.009)**	0.845 (0.008)	0.841 (0.009)
		*N* = 2000	0.855 (0.005)	0.855 (0.006)	**0.858 (0.007)**	0.854 (0.007)	0.851 (0.007)
		*N* = 5000	0.858 (0.004)	0.857 (0.004)	**0.858 (0.004)**	0.858 (0.004)	0.855 (0.004)
	*p* = 200	*N* = 500	0.841 (0.016)	0.823 (0.019)	**0.854 (0.014)**	0.734 (0.039)	0.784 (0.018)
		*N* = 1000	0.854 (0.009)	0.843 (0.01)	**0.855 (0.009)**	0.827 (0.012)	0.817 (0.01)
		*N* = 2000	0.856 (0.007)	0.854 (0.006)	**0.857 (0.007)**	0.85 (0.008)	0.839 (0.006)
		*N* = 5000	0.858 (0.004)	0.858 (0.004)	**0.858 (0.004)**	0.856 (0.004)	0.853 (0.004)
	*p* = 500	*N* = 500	0.837 (0.016)	0.812 (0.021)	**0.852 (0.014)**	0.647 (0.023)	0.759 (0.017)
		*N* = 1000	0.85 (0.01)	0.837 (0.011)	**0.856 (0.009)**	0.739 (0.014)	0.775 (0.015)
		*N* = 2000	0.855 (0.006)	0.85 (0.007)	**0.858 (0.006)**	0.83 (0.008)	0.808 (0.009)
		*N* = 5000	0.858 (0.004)	0.856 (0.004)	**0.858 (0.004)**	0.853 (0.005)	0.84 (0.004)
	*p* = 1000	*N* = 500	0.832 (0.022)	0.81 (0.019)	**0.846 (0.014)**	0.629 (0.021)	0.755 (0.026)
		*N* = 1000	0.85 (0.009)	0.828 (0.013)	**0.856 (0.009)**	0.67 (0.012)	0.764 (0.015)
		*N* = 2000	0.856 (0.006)	0.846 (0.007)	**0.858 (0.006)**	0.785 (0.01)	0.776 (0.008)
		*N* = 5000	0.857 (0.004)	0.855 (0.005)	**0.857 (0.004)**	0.844 (0.005)	0.819 (0.005)
**Σ_2_**	*p* = 50	*N* = 500	0.789 (0.018)	0.789 (0.02)	**0.8 (0.015)**	0.789 (0.017)	0.78 (0.016)
		*N* = 1000	0.805 (0.012)	0.799 (0.046)	**0.811 (0.01)**	0.804 (0.014)	0.806 (0.01)
		*N* = 2000	0.812 (0.007)	0.799 (0.065)	**0.813 (0.007)**	0.806 (0.008)	0.812 (0.006)
		*N* = 5000	0.814 (0.005)	0.812 (0.064)	**0.814 (0.004)**	0.813 (0.004)	0.811 (0.006)
	*p* = 100	*N* = 500	0.783 (0.024)	0.776 (0.024)	**0.798 (0.02)**	0.766 (0.021)	0.759 (0.017)
		*N* = 1000	0.807 (0.012)	0.797 (0.043)	**0.81 (0.01)**	0.794 (0.012)	0.785 (0.014)
		*N* = 2000	0.812 (0.008)	0.806 (0.025)	**0.813 (0.007)**	0.807 (0.007)	0.8 (0.007)
		*N* = 5000	0.814 (0.005)	0.802 (0.056)	**0.814 (0.004)**	0.813 (0.006)	0.812 (0.004)
	*p* = 200	*N* = 500	0.777 (0.023)	0.757 (0.024)	**0.794 (0.018)**	0.652 (0.032)	0.696 (0.028)
		*N* = 1000	0.806 (0.013)	0.794 (0.012)	**0.809 (0.013)**	0.766 (0.014)	0.762 (0.012)
		*N* = 2000	0.811 (0.008)	0.802 (0.04)	**0.813 (0.007)**	0.798 (0.01)	0.788 (0.009)
		*N* = 5000	0.813 (0.004)	0.806 (0.043)	**0.815 (0.004)**	0.811 (0.004)	0.804 (0.005)
	*p* = 500	*N* = 500	0.763 (0.032)	0.732 (0.028)	**0.786 (0.021)**	0.58 (0.03)	0.649 (0.028)
		*N* = 1000	0.801 (0.013)	0.775 (0.014)	**0.807 (0.012)**	0.647 (0.017)	0.689 (0.018)
		*N* = 2000	0.809 (0.007)	0.8 (0.009)	**0.813 (0.008)**	0.765 (0.011)	0.74 (0.008)
		*N* = 5000	**0.814 (0.005)**	0.81 (0.032)	0.813 (0.004)	0.805 (0.008)	0.788 (0.006)
	*p* = 1000	*N* = 500	0.756 (0.029)	0.732 (0.032)	**0.766 (0.032)**	0.576 (0.033)	0.652 (0.019)
		*N* = 1000	0.799 (0.015)	0.768 (0.014)	**0.805 (0.012)**	0.601 (0.015)	0.661 (0.017)
		*N* = 2000	0.81 (0.008)	0.793 (0.01)	**0.813 (0.007)**	0.705 (0.01)	0.689 (0.012)
		*N* = 5000	0.814 (0.004)	0.81 (0.005)	**0.815 (0.004)**	0.79 (0.005)	0.756 (0.006)

**Table A5:** Simulation 1, Scenario 3: mean (SD) of C-index.

			CNT	CNL	CT	CPH	RSF
**Σ_1_**	*p* = 50	*N* = 500	**0.679 (0.024)**	0.674 (0.033)	0.677 (0.026)	0.665 (0.023)	0.659 (0.027)
		*N* = 1000	0.691 (0.019)	**0.726 (0.063)**	0.693 (0.019)	0.686 (0.018)	0.682 (0.014)
		*N* = 2000	0.701 (0.011)	**0.759 (0.077)**	0.702 (0.013)	0.702 (0.009)	0.699 (0.011)
		*N* = 5000	0.705 (0.008)	**0.85 (0.03)**	0.707 (0.006)	0.707 (0.008)	0.705 (0.007)
	*p* = 100	*N* = 500	0.667 (0.027)	0.649 (0.031)	**0.669 (0.027)**	0.623 (0.027)	0.649 (0.024)
		*N* = 1000	0.691 (0.017)	**0.717 (0.053)**	0.69 (0.017)	0.679 (0.017)	0.667 (0.018)
		*N* = 2000	0.7 (0.011)	**0.729 (0.088)**	0.699 (0.014)	0.693 (0.015)	0.688 (0.007)
		*N* = 5000	0.707 (0.007)	**0.757 (0.083)**	0.705 (0.008)	0.704 (0.008)	0.701 (0.007)
	*p* = 200	*N* = 500	**0.668 (0.026)**	0.621 (0.033)	0.656 (0.026)	0.586 (0.018)	0.602 (0.021)
		*N* = 1000	**0.69 (0.017)**	0.674 (0.03)	0.677 (0.022)	0.631 (0.021)	0.643 (0.014)
		*N* = 2000	0.698 (0.011)	**0.742 (0.062)**	0.694 (0.014)	0.68 (0.012)	0.673 (0.011)
		*N* = 5000	0.705 (0.007)	**0.745 (0.07)**	0.7 (0.008)	0.701 (0.007)	0.692 (0.007)
	*p* = 500	*N* = 500	**0.654 (0.033)**	0.598 (0.028)	0.638 (0.03)	0.549 (0.028)	0.589 (0.028)
		*N* = 1000	**0.679 (0.017)**	0.634 (0.026)	0.659 (0.021)	0.561 (0.016)	0.596 (0.015)
		*N* = 2000	**0.695 (0.012)**	0.691 (0.028)	0.684 (0.016)	0.639 (0.017)	0.624 (0.013)
		*N* = 5000	0.704 (0.007)	**0.769 (0.072)**	0.698 (0.009)	0.69 (0.006)	0.668 (0.007)
	*p* = 1000	*N* = 500	**0.649 (0.031)**	0.6 (0.023)	0.625 (0.033)	0.544 (0.026)	0.588 (0.017)
		*N* = 1000	**0.676 (0.021)**	0.617 (0.023)	0.655 (0.023)	0.555 (0.021)	0.591 (0.02)
		*N* = 2000	**0.694 (0.012)**	0.656 (0.023)	0.673 (0.018)	0.594 (0.026)	0.591 (0.012)
		*N* = 5000	0.704 (0.007)	**0.799 (0.051)**	0.692 (0.009)	0.671 (0.007)	0.633 (0.008)
**Σ_2_**	*p* = 50	*N* = 500	0.628 (0.027)	0.6 (0.054)	**0.63 (0.026)**	0.63 (0.034)	0.632 (0.028)
		*N* = 1000	0.652 (0.018)	0.639 (0.042)	**0.654 (0.019)**	0.647 (0.02)	0.645 (0.019)
		*N* = 2000	**0.666 (0.013)**	0.661 (0.026)	0.663 (0.012)	0.662 (0.013)	0.664 (0.008)
		*N* = 5000	0.678 (0.007)	**0.78 (0.025)**	0.675 (0.008)	0.676 (0.008)	0.675 (0.008)
	*p* = 100	*N* = 500	0.605 (0.03)	0.586 (0.033)	**0.606 (0.027)**	0.591 (0.019)	0.584 (0.032)
		*N* = 1000	**0.641 (0.022)**	0.6 (0.051)	0.634 (0.019)	0.626 (0.019)	0.62 (0.016)
		*N* = 2000	**0.663 (0.014)**	0.626 (0.052)	0.647 (0.012)	0.652 (0.018)	0.65 (0.011)
		*N* = 5000	**0.677 (0.007)**	0.671 (0.009)	0.665 (0.008)	0.672 (0.006)	0.667 (0.007)
	*p* = 200	*N* = 500	0.59 (0.028)	0.569 (0.03)	**0.592 (0.029)**	0.555 (0.028)	0.574 (0.024)
		*N* = 1000	**0.632 (0.024)**	0.604 (0.038)	0.617 (0.021)	0.591 (0.024)	0.592 (0.022)
		*N* = 2000	**0.662 (0.014)**	0.586 (0.064)	0.634 (0.015)	0.641 (0.011)	0.621 (0.012)
		*N* = 5000	**0.677 (0.008)**	0.645 (0.04)	0.654 (0.01)	0.664 (0.01)	0.654 (0.005)
	*p* = 500	*N* = 500	0.565 (0.032)	0.551 (0.027)	**0.574 (0.027)**	0.524 (0.023)	0.553 (0.027)
		*N* = 1000	**0.616 (0.025)**	0.587 (0.024)	0.6 (0.022)	0.545 (0.022)	0.56 (0.013)
		*N* = 2000	**0.653 (0.018)**	0.625 (0.031)	0.62 (0.017)	0.598 (0.023)	0.586 (0.016)
		*N* = 5000	**0.674 (0.009)**	0.583 (0.071)	0.638 (0.012)	0.651 (0.01)	0.628 (0.005)
	*p* = 1000	*N* = 500	0.552 (0.031)	0.544 (0.033)	**0.553 (0.033)**	0.521 (0.028)	0.54 (0.02)
		*N* = 1000	**0.598 (0.022)**	0.572 (0.022)	0.583 (0.024)	0.536 (0.018)	0.542 (0.016)
		*N* = 2000	**0.646 (0.014)**	0.604 (0.022)	0.608 (0.016)	0.558 (0.013)	0.564 (0.011)
		*N* = 5000	**0.675 (0.008)**	0.637 (0.058)	0.629 (0.013)	0.625 (0.006)	0.599 (0.009)

**Table A6:** Simulation 1, Scenario 4: mean (SD) of C-index.

			CNT	CNL	CT	CPH	RSF
**Σ_1_**	*p* = 50	*N* = 500	0.664 (0.028)	**0.668 (0.049)**	0.66 (0.022)	0.658 (0.025)	0.654 (0.017)
		*N* = 1000	0.677 (0.014)	**0.703 (0.064)**	0.678 (0.02)	0.679 (0.018)	0.669 (0.013)
		*N* = 2000	0.686 (0.012)	**0.741 (0.073)**	0.686 (0.011)	0.677 (0.012)	0.686 (0.011)
		*N* = 5000	0.69 (0.008)	**0.848 (0.022)**	0.692 (0.007)	0.69 (0.006)	0.69 (0.007)
	*p* = 100	*N* = 500	**0.657 (0.021)**	0.632 (0.033)	0.648 (0.028)	0.622 (0.026)	0.618 (0.025)
		*N* = 1000	0.676 (0.018)	**0.69 (0.057)**	0.669 (0.02)	0.663 (0.018)	0.655 (0.021)
		*N* = 2000	0.684 (0.013)	**0.702 (0.078)**	0.68 (0.013)	0.679 (0.012)	0.668 (0.013)
		*N* = 5000	0.691 (0.008)	**0.748 (0.082)**	0.689 (0.008)	0.692 (0.008)	0.684 (0.008)
	*p* = 200	*N* = 500	**0.647 (0.027)**	0.602 (0.031)	0.639 (0.029)	0.574 (0.023)	0.594 (0.021)
		*N* = 1000	**0.675 (0.016)**	0.651 (0.032)	0.659 (0.019)	0.621 (0.016)	0.622 (0.018)
		*N* = 2000	0.684 (0.013)	**0.719 (0.065)**	0.674 (0.014)	0.666 (0.013)	0.652 (0.016)
		*N* = 5000	0.691 (0.008)	**0.725 (0.076)**	0.684 (0.008)	0.684 (0.006)	0.679 (0.006)
	*p* = 500	*N* = 500	**0.635 (0.03)**	0.582 (0.031)	0.618 (0.032)	0.543 (0.027)	0.579 (0.024)
		*N* = 1000	**0.666 (0.021)**	0.618 (0.026)	0.646 (0.021)	0.561 (0.016)	0.576 (0.017)
		*N* = 2000	**0.681 (0.011)**	0.673 (0.024)	0.664 (0.016)	0.611 (0.022)	0.613 (0.011)
		*N* = 5000	0.69 (0.007)	**0.731 (0.077)**	0.678 (0.009)	0.674 (0.006)	0.652 (0.007)
	*p* = 1000	*N* = 500	**0.625 (0.033)**	0.584 (0.034)	0.606 (0.032)	0.533 (0.028)	0.589 (0.028)
		*N* = 1000	**0.667 (0.017)**	0.601 (0.02)	0.629 (0.022)	0.546 (0.017)	0.581 (0.02)
		*N* = 2000	**0.68 (0.011)**	0.639 (0.022)	0.655 (0.017)	0.583 (0.022)	0.59 (0.008)
		*N* = 5000	0.689 (0.008)	**0.749 (0.056)**	0.67 (0.009)	0.657 (0.009)	0.62 (0.01)
**Σ_2_**	*p* = 50	*N* = 500	0.617 (0.029)	0.593 (0.047)	0.616 (0.026)	**0.62 (0.03)**	0.613 (0.028)
		*N* = 1000	0.636 (0.018)	0.622 (0.037)	0.634 (0.017)	**0.644 (0.017)**	0.633 (0.018)
		*N* = 2000	0.651 (0.013)	0.652 (0.02)	0.65 (0.011)	**0.653 (0.013)**	0.65 (0.011)
		*N* = 5000	0.663 (0.007)	**0.774 (0.021)**	0.659 (0.009)	0.663 (0.006)	0.661 (0.009)
	*p* = 100	*N* = 500	0.592 (0.032)	0.58 (0.038)	**0.598 (0.027)**	0.587 (0.026)	0.59 (0.028)
		*N* = 1000	**0.629 (0.021)**	0.582 (0.048)	0.619 (0.022)	0.611 (0.021)	0.615 (0.019)
		*N* = 2000	**0.651 (0.012)**	0.62 (0.042)	0.636 (0.013)	0.645 (0.012)	0.636 (0.011)
		*N* = 5000	**0.662 (0.008)**	0.657 (0.009)	0.654 (0.007)	0.661 (0.008)	0.655 (0.007)
	*p* = 200	*N* = 500	0.576 (0.027)	0.562 (0.03)	**0.578 (0.029)**	0.549 (0.028)	0.558 (0.019)
		*N* = 1000	**0.614 (0.022)**	0.593 (0.04)	0.602 (0.022)	0.591 (0.014)	0.594 (0.021)
		*N* = 2000	**0.647 (0.014)**	0.561 (0.063)	0.619 (0.014)	0.624 (0.014)	0.617 (0.016)
		*N* = 5000	**0.662 (0.008)**	0.637 (0.019)	0.638 (0.009)	0.649 (0.009)	0.641 (0.008)
	*p* = 500	*N* = 500	0.556 (0.03)	0.543 (0.032)	**0.558 (0.025)**	0.529 (0.021)	0.539 (0.022)
		*N* = 1000	**0.597 (0.026)**	0.568 (0.025)	0.579 (0.026)	0.544 (0.019)	0.558 (0.019)
		*N* = 2000	**0.639 (0.014)**	0.606 (0.034)	0.606 (0.019)	0.583 (0.021)	0.575 (0.014)
		*N* = 5000	**0.66 (0.009)**	0.566 (0.061)	0.619 (0.012)	0.636 (0.007)	0.615 (0.005)
	*p* = 1000	*N* = 500	0.544 (0.026)	0.541 (0.027)	**0.547 (0.024)**	0.525 (0.022)	0.547 (0.023)
		*N* = 1000	**0.587 (0.027)**	0.557 (0.026)	0.566 (0.024)	0.534 (0.016)	0.546 (0.02)
		*N* = 2000	**0.628 (0.02)**	0.587 (0.021)	0.592 (0.018)	0.545 (0.023)	0.556 (0.011)
		*N* = 5000	**0.659 (0.008)**	0.62 (0.054)	0.608 (0.011)	0.614 (0.007)	0.585 (0.007)

**Table A7:** Simulation 1, Scenario 1: mean (SD) of variable-selection measures.

			CNT	CNL	CT	CPH	RSF
			F_1_				
**Σ_1_**	*p* = 50	*N* = 500	0.767 (0.134)	0.62 (0.065)	**0.905 (0.073)**	0.437 (0.044)	0.408 (0.029)
		*N* = 1000	0.877 (0.1)	0.748 (0.082)	**0.969 (0.044)**	0.497 (0.053)	0.443 (0.027)
		*N* = 2000	0.937 (0.078)	0.873 (0.075)	**0.993 (0.022)**	0.605 (0.087)	0.518 (0.048)
		*N* = 5000	0.983 (0.035)	0.974 (0.047)	**1.0 (0.0)**	0.786 (0.097)	0.655 (0.062)
	*p* = 100	*N* = 500	0.764 (0.182)	0.449 (0.063)	**0.891 (0.115)**	0.259 (0.014)	0.228 (0.01)
		*N* = 1000	0.848 (0.142)	0.585 (0.074)	**0.942 (0.072)**	0.315 (0.024)	0.262 (0.016)
		*N* = 2000	0.929 (0.092)	0.785 (0.08)	**0.993 (0.022)**	0.406 (0.044)	0.311 (0.023)
		*N* = 5000	0.981 (0.043)	0.971 (0.036)	**1.0 (0.005)**	0.698 (0.123)	0.453 (0.037)
	*p* = 200	*N* = 500	0.784 (0.204)	0.328 (0.038)	**0.89 (0.137)**	0.131 (0.01)	0.122 (0.005)
		*N* = 1000	0.879 (0.158)	0.448 (0.061)	**0.965 (0.066)**	0.18 (0.009)	0.139 (0.008)
		*N* = 2000	0.931 (0.104)	0.656 (0.079)	**0.987 (0.029)**	0.25 (0.013)	0.169 (0.008)
		*N* = 5000	0.989 (0.029)	0.952 (0.046)	**1.0 (0.005)**	0.532 (0.051)	0.268 (0.02)
	*p* = 500	*N* = 500	0.774 (0.222)	0.251 (0.04)	**0.94 (0.114)**	0.064 (0.006)	0.054 (0.001)
		*N* = 1000	0.91 (0.142)	0.306 (0.046)	**0.977 (0.061)**	0.072 (0.003)	0.056 (0.001)
		*N* = 2000	0.979 (0.081)	0.503 (0.081)	**0.994 (0.019)**	0.109 (0.007)	0.066 (0.003)
		*N* = 5000	0.992 (0.023)	0.914 (0.069)	**0.999 (0.007)**	0.295 (0.03)	0.113 (0.011)
	*p* = 1000	*N* = 500	0.768 (0.193)	0.289 (0.045)	**0.935 (0.118)**	0.047 (0.001)	0.032 (0.002)
		*N* = 1000	0.895 (0.16)	0.284 (0.037)	**0.995 (0.03)**	0.044 (0.001)	0.033 (0.001)
		*N* = 2000	0.976 (0.072)	0.431 (0.079)	**0.992 (0.035)**	0.056 (0.002)	0.034 (0.001)
		*N* = 5000	0.986 (0.069)	0.893 (0.086)	**1.0 (0.0)**	0.163 (0.014)	0.052 (0.003)
**Σ_2_**	*p* = 50	*N* = 500	0.669 (0.176)	0.573 (0.069)	**0.816 (0.109)**	0.427 (0.038)	0.415 (0.03)
		*N* = 1000	0.88 (0.164)	0.688 (0.085)	**0.924 (0.082)**	0.507 (0.041)	0.467 (0.042)
		*N* = 2000	0.981 (0.066)	0.838 (0.117)	**0.981 (0.036)**	0.559 (0.074)	0.536 (0.044)
		*N* = 5000	1.0 (0.0)	0.947 (0.111)	**1.0 (0.0)**	0.81 (0.086)	0.688 (0.049)
	*p* = 100	*N* = 500	0.658 (0.228)	0.397 (0.05)	**0.794 (0.167)**	0.26 (0.017)	0.225 (0.014)
		*N* = 1000	0.903 (0.149)	0.535 (0.079)	**0.936 (0.114)**	0.32 (0.022)	0.267 (0.019)
		*N* = 2000	0.988 (0.028)	0.725 (0.103)	**0.983 (0.049)**	0.411 (0.044)	0.324 (0.02)
		*N* = 5000	0.999 (0.01)	0.963 (0.086)	**0.996 (0.013)**	0.702 (0.089)	0.509 (0.041)
	*p* = 200	*N* = 500	0.689 (0.22)	0.235 (0.047)	**0.828 (0.189)**	0.12 (0.011)	0.116 (0.004)
		*N* = 1000	0.904 (0.106)	0.367 (0.045)	**0.974 (0.082)**	0.168 (0.011)	0.136 (0.005)
		*N* = 2000	0.979 (0.055)	0.583 (0.096)	**0.998 (0.015)**	0.244 (0.018)	0.176 (0.008)
		*N* = 5000	0.996 (0.019)	0.899 (0.159)	**0.999 (0.008)**	0.494 (0.064)	0.314 (0.026)
	*p* = 500	*N* = 500	0.747 (0.218)	0.168 (0.024)	**0.799 (0.172)**	0.061 (0.007)	0.049 (0.002)
		*N* = 1000	0.853 (0.158)	0.228 (0.029)	**0.983 (0.051)**	0.064 (0.002)	0.053 (0.002)
		*N* = 2000	0.974 (0.059)	0.387 (0.068)	**1.0 (0.0)**	0.099 (0.006)	0.066 (0.003)
		*N* = 5000	0.997 (0.021)	0.874 (0.108)	**1.0 (0.0)**	0.255 (0.024)	0.123 (0.008)
	*p* = 1000	*N* = 500	0.706 (0.249)	0.164 (0.031)	**0.734 (0.188)**	0.041 (0.01)	0.027 (0.001)
		*N* = 1000	0.871 (0.152)	0.2 (0.022)	**0.978 (0.038)**	0.041 (0.001)	0.028 (0.001)
		*N* = 2000	0.972 (0.05)	0.3 (0.05)	**1.0 (0.005)**	0.047 (0.002)	0.031 (0.001)
		*N* = 5000	0.994 (0.03)	0.796 (0.094)	**1.0 (0.0)**	0.134 (0.009)	0.053 (0.002)
			**AUC**				
**Σ_1_**	*p* = 50	*N* = 500	1.0 (0.0)	1.0 (0.0)	**1.0 (0.0)**	1.0 (0.0)	1.0 (0.0)
		*N* = 1000	1.0 (0.0)	1.0 (0.0)	**1.0 (0.0)**	1.0 (0.0)	1.0 (0.0)
		*N* = 2000	1.0 (0.0)	1.0 (0.0)	**1.0 (0.0)**	1.0 (0.0)	1.0 (0.0)
		*N* = 5000	1.0 (0.0)	1.0 (0.0)	**1.0 (0.0)**	1.0 (0.0)	1.0 (0.0)
	*p* = 100	*N* = 500	1.0 (0.0)	1.0 (0.0)	**1.0 (0.0)**	1.0 (0.0)	1.0 (0.0)
		*N* = 1000	1.0 (0.0)	1.0 (0.0)	**1.0 (0.0)**	1.0 (0.0)	1.0 (0.0)
		*N* = 2000	1.0 (0.0)	1.0 (0.0)	**1.0 (0.0)**	1.0 (0.0)	1.0 (0.0)
		*N* = 5000	1.0 (0.0)	1.0 (0.0)	**1.0 (0.0)**	1.0 (0.0)	1.0 (0.0)
	*p* = 200	*N* = 500	1.0 (0.004)	1.0 (0.0)	**1.0 (0.0)**	1.0 (0.001)	1.0 (0.0)
		*N* = 1000	1.0 (0.0)	1.0 (0.0)	**1.0 (0.0)**	1.0 (0.0)	1.0 (0.0)
		*N* = 2000	1.0 (0.0)	1.0 (0.0)	**1.0 (0.0)**	1.0 (0.0)	1.0 (0.0)
		*N* = 5000	1.0 (0.0)	1.0 (0.0)	**1.0 (0.0)**	1.0 (0.0)	1.0 (0.0)
	*p* = 500	*N* = 500	1.0 (0.004)	1.0 (0.0)	**1.0 (0.0)**	0.992 (0.021)	1.0 (0.0)
		*N* = 1000	1.0 (0.0)	1.0 (0.0)	**1.0 (0.0)**	1.0 (0.0)	1.0 (0.0)
		*N* = 2000	1.0 (0.0)	1.0 (0.0)	**1.0 (0.0)**	1.0 (0.0)	1.0 (0.0)
		*N* = 5000	1.0 (0.0)	1.0 (0.0)	**1.0 (0.0)**	1.0 (0.0)	1.0 (0.0)
	*p* = 1000	*N* = 500	0.999 (0.006)	1.0 (0.0)	**1.0 (0.0)**	0.999 (0.002)	1.0 (0.0)
		*N* = 1000	1.0 (0.0)	1.0 (0.0)	**1.0 (0.0)**	1.0 (0.0)	1.0 (0.0)
		*N* = 2000	1.0 (0.0)	1.0 (0.0)	**1.0 (0.0)**	1.0 (0.0)	1.0 (0.0)
		*N* = 5000	1.0 (0.0)	1.0 (0.0)	**1.0 (0.0)**	1.0 (0.0)	1.0 (0.0)
**Σ_2_**	*p* = 50	*N* = 500	1.0 (0.0)	0.989 (0.083)	**1.0 (0.0)**	1.0 (0.0)	1.0 (0.0)
		*N* = 1000	1.0 (0.0)	0.99 (0.068)	**1.0 (0.0)**	1.0 (0.0)	1.0 (0.0)
		*N* = 2000	1.0 (0.0)	0.995 (0.048)	**1.0 (0.0)**	1.0 (0.0)	1.0 (0.0)
		*N* = 5000	1.0 (0.0)	0.996 (0.037)	**1.0 (0.0)**	1.0 (0.0)	1.0 (0.0)
	*p* = 100	*N* = 500	1.0 (0.0)	1.0 (0.0)	**1.0 (0.0)**	1.0 (0.0)	1.0 (0.0)
		*N* = 1000	1.0 (0.0)	1.0 (0.0)	**1.0 (0.0)**	1.0 (0.0)	1.0 (0.0)
		*N* = 2000	1.0 (0.0)	0.994 (0.06)	**1.0 (0.0)**	1.0 (0.0)	1.0 (0.0)
		*N* = 5000	1.0 (0.0)	0.995 (0.052)	**1.0 (0.0)**	1.0 (0.0)	1.0 (0.0)
	*p* = 200	*N* = 500	0.999 (0.005)	1.0 (0.0)	**1.0 (0.0)**	0.996 (0.007)	1.0 (0.0)
		*N* = 1000	1.0 (0.0)	1.0 (0.0)	**1.0 (0.0)**	1.0 (0.0)	1.0 (0.0)
		*N* = 2000	1.0 (0.0)	0.995 (0.052)	**1.0 (0.0)**	1.0 (0.0)	1.0 (0.0)
		*N* = 5000	1.0 (0.0)	0.991 (0.065)	**1.0 (0.0)**	1.0 (0.0)	1.0 (0.0)
	*p* = 500	*N* = 500	0.997 (0.014)	1.0 (0.0)	**1.0 (0.0)**	0.98 (0.036)	1.0 (0.0)
		*N* = 1000	1.0 (0.0)	1.0 (0.0)	**1.0 (0.0)**	1.0 (0.0)	1.0 (0.0)
		*N* = 2000	1.0 (0.0)	1.0 (0.0)	**1.0 (0.0)**	1.0 (0.0)	1.0 (0.0)
		*N* = 5000	1.0 (0.0)	0.995 (0.054)	**1.0 (0.0)**	1.0 (0.0)	1.0 (0.0)
	*p* = 1000	*N* = 500	0.997 (0.013)	1.0 (0.0)	**1.0 (0.0)**	0.972 (0.051)	1.0 (0.0)
		*N* = 1000	1.0 (0.0)	1.0 (0.0)	**1.0 (0.0)**	1.0 (0.0)	1.0 (0.0)
		*N* = 2000	1.0 (0.0)	1.0 (0.0)	**1.0 (0.0)**	1.0 (0.0)	1.0 (0.0)
		*N* = 5000	1.0 (0.0)	1.0 (0.0)	**1.0 (0.0)**	1.0 (0.0)	1.0 (0.0)

**Table A8:** Simulation 1, Scenario 2: mean (SD) of variable-selection measures.

			CNT	CNL	CT	CPH	RSF
			F_1_				
**Σ_1_**	*p* = 50	*N* = 500	0.724 (0.163)	0.551 (0.066)	**0.808 (0.116)**	0.441 (0.035)	0.426 (0.02)
		*N* = 1000	0.815 (0.127)	0.61 (0.069)	**0.86 (0.095)**	0.486 (0.047)	0.443 (0.025)
		*N* = 2000	0.886 (0.042)	0.694 (0.081)	**0.902 (0.045)**	0.561 (0.056)	0.487 (0.042)
		*N* = 5000	0.902 (0.023)	0.732 (0.139)	**0.91 (0.005)**	0.608 (0.087)	0.533 (0.041)
	*p* = 100	*N* = 500	0.738 (0.145)	0.365 (0.04)	**0.806 (0.121)**	0.26 (0.02)	0.239 (0.01)
		*N* = 1000	0.788 (0.112)	0.457 (0.055)	**0.876 (0.066)**	0.292 (0.025)	0.249 (0.012)
		*N* = 2000	0.874 (0.056)	0.573 (0.088)	**0.902 (0.036)**	0.352 (0.044)	0.279 (0.017)
		*N* = 5000	0.905 (0.019)	0.766 (0.094)	**0.909 (0.0)**	0.514 (0.097)	0.34 (0.026)
	*p* = 200	*N* = 500	0.708 (0.167)	0.237 (0.034)	**0.794 (0.103)**	0.14 (0.015)	0.128 (0.007)
		*N* = 1000	0.773 (0.127)	0.301 (0.039)	**0.869 (0.064)**	0.164 (0.013)	0.135 (0.005)
		*N* = 2000	0.858 (0.082)	0.427 (0.06)	**0.904 (0.018)**	0.204 (0.015)	0.149 (0.008)
		*N* = 5000	0.896 (0.053)	0.686 (0.124)	**0.909 (0.006)**	0.349 (0.04)	0.191 (0.014)
	*p* = 500	*N* = 500	0.658 (0.199)	0.163 (0.025)	**0.731 (0.141)**	0.072 (0.005)	0.058 (0.002)
		*N* = 1000	0.761 (0.167)	0.177 (0.027)	**0.837 (0.097)**	0.072 (0.004)	0.058 (0.002)
		*N* = 2000	0.858 (0.091)	0.258 (0.041)	**0.897 (0.03)**	0.09 (0.007)	0.062 (0.003)
		*N* = 5000	0.886 (0.087)	0.593 (0.101)	**0.91 (0.005)**	0.162 (0.017)	0.077 (0.007)
	*p* = 1000	*N* = 500	0.626 (0.195)	0.15 (0.032)	**0.65 (0.16)**	0.051 (0.003)	0.033 (0.003)
		*N* = 1000	0.791 (0.146)	0.136 (0.023)	**0.827 (0.098)**	0.047 (0.003)	0.031 (0.002)
		*N* = 2000	0.854 (0.09)	0.182 (0.026)	**0.885 (0.049)**	0.049 (0.004)	0.032 (0.001)
		*N* = 5000	0.9 (0.047)	0.482 (0.099)	**0.909 (0.0)**	0.084 (0.006)	0.038 (0.002)
**Σ_2_**	*p* = 50	*N* = 500	0.621 (0.162)	0.503 (0.068)	**0.711 (0.13)**	0.446 (0.034)	0.428 (0.019)
		*N* = 1000	0.759 (0.15)	0.574 (0.081)	**0.834 (0.091)**	0.488 (0.049)	0.466 (0.029)
		*N* = 2000	0.858 (0.074)	0.673 (0.123)	**0.882 (0.052)**	0.537 (0.042)	0.482 (0.037)
		*N* = 5000	0.899 (0.028)	0.741 (0.155)	**0.909 (0.001)**	0.658 (0.075)	0.565 (0.046)
	*p* = 100	*N* = 500	0.626 (0.198)	0.331 (0.042)	**0.668 (0.129)**	0.25 (0.021)	0.242 (0.012)
		*N* = 1000	0.778 (0.119)	0.415 (0.055)	**0.805 (0.101)**	0.287 (0.027)	0.263 (0.012)
		*N* = 2000	0.826 (0.097)	0.525 (0.098)	**0.881 (0.043)**	0.342 (0.054)	0.292 (0.02)
		*N* = 5000	0.898 (0.024)	0.734 (0.178)	**0.906 (0.014)**	0.484 (0.069)	0.351 (0.029)
	*p* = 200	*N* = 500	0.569 (0.198)	0.196 (0.034)	**0.578 (0.118)**	0.134 (0.011)	0.128 (0.007)
		*N* = 1000	**0.741 (0.147)**	0.262 (0.029)	0.726 (0.112)	0.163 (0.014)	0.135 (0.008)
		*N* = 2000	0.811 (0.122)	0.369 (0.063)	**0.848 (0.06)**	0.194 (0.016)	0.157 (0.01)
		*N* = 5000	0.873 (0.086)	0.645 (0.171)	**0.904 (0.015)**	0.315 (0.025)	0.198 (0.017)
	*p* = 500	*N* = 500	**0.515 (0.22)**	0.129 (0.02)	0.465 (0.124)	0.063 (0.011)	0.055 (0.003)
		*N* = 1000	**0.7 (0.174)**	0.141 (0.018)	0.602 (0.14)	0.064 (0.006)	0.056 (0.003)
		*N* = 2000	**0.793 (0.133)**	0.204 (0.031)	0.779 (0.102)	0.086 (0.006)	0.062 (0.003)
		*N* = 5000	0.882 (0.053)	0.51 (0.089)	**0.906 (0.013)**	0.153 (0.017)	0.084 (0.006)
	*p* = 1000	*N* = 500	**0.462 (0.188)**	0.12 (0.025)	0.343 (0.126)	0.044 (0.01)	0.03 (0.002)
		*N* = 1000	**0.661 (0.178)**	0.112 (0.015)	0.521 (0.141)	0.043 (0.005)	0.029 (0.002)
		*N* = 2000	**0.812 (0.141)**	0.136 (0.02)	0.741 (0.126)	0.044 (0.003)	0.031 (0.001)
		*N* = 5000	0.889 (0.045)	0.383 (0.077)	**0.897 (0.028)**	0.078 (0.006)	0.038 (0.003)
			**AUC**				
**Σ_1_**	*p* = 50	*N* = 500	0.923 (0.032)	0.93 (0.034)	0.919 (0.021)	0.939 (0.044)	**0.947 (0.028)**
		*N* = 1000	0.92 (0.017)	0.922 (0.038)	0.921 (0.017)	**0.94 (0.033)**	0.937 (0.043)
		*N* = 2000	0.917 (0.012)	0.941 (0.042)	0.918 (0.008)	**0.952 (0.036)**	0.944 (0.033)
		*N* = 5000	0.916 (0.002)	0.951 (0.042)	0.917 (0.004)	0.942 (0.036)	**0.96 (0.034)**
	*p* = 100	*N* = 500	0.918 (0.019)	0.924 (0.034)	0.917 (0.014)	0.934 (0.036)	**0.945 (0.036)**
		*N* = 1000	0.918 (0.015)	0.921 (0.04)	0.917 (0.007)	**0.927 (0.045)**	0.923 (0.033)
		*N* = 2000	0.918 (0.013)	0.934 (0.038)	0.916 (0.001)	**0.949 (0.037)**	0.942 (0.034)
		*N* = 5000	0.916 (0.001)	0.931 (0.042)	0.917 (0.0)	0.944 (0.045)	**0.949 (0.037)**
	*p* = 200	*N* = 500	0.914 (0.02)	**0.928 (0.034)**	0.919 (0.016)	0.908 (0.033)	0.928 (0.038)
		*N* = 1000	0.919 (0.013)	0.926 (0.039)	0.919 (0.01)	0.929 (0.033)	**0.94 (0.031)**
		*N* = 2000	0.919 (0.01)	0.927 (0.034)	0.917 (0.004)	0.934 (0.033)	**0.941 (0.024)**
		*N* = 5000	0.917 (0.006)	0.93 (0.038)	0.917 (0.004)	**0.951 (0.03)**	0.949 (0.031)
	*p* = 500	*N* = 500	0.916 (0.017)	0.93 (0.038)	0.919 (0.012)	0.924 (0.042)	**0.935 (0.031)**
		*N* = 1000	0.916 (0.008)	0.926 (0.039)	0.919 (0.011)	0.935 (0.038)	**0.935 (0.026)**
		*N* = 2000	0.917 (0.006)	0.928 (0.036)	0.918 (0.007)	0.937 (0.04)	**0.95 (0.043)**
		*N* = 5000	0.917 (0.004)	**0.934 (0.036)**	0.917 (0.004)	0.934 (0.038)	0.927 (0.037)
	*p* = 1000	*N* = 500	0.913 (0.017)	0.933 (0.038)	0.917 (0.013)	0.944 (0.029)	**0.952 (0.034)**
		*N* = 1000	0.917 (0.007)	0.93 (0.036)	0.918 (0.007)	**0.948 (0.03)**	0.931 (0.048)
		*N* = 2000	0.917 (0.006)	0.928 (0.035)	0.917 (0.006)	0.939 (0.029)	**0.939 (0.031)**
		*N* = 5000	0.917 (0.006)	0.929 (0.035)	0.917 (0.0)	0.936 (0.041)	**0.941 (0.031)**
**Σ_2_**	*p* = 50	*N* = 500	0.924 (0.033)	0.924 (0.038)	0.923 (0.03)	0.92 (0.044)	**0.942 (0.04)**
		*N* = 1000	0.914 (0.022)	0.908 (0.085)	0.918 (0.016)	0.935 (0.033)	**0.95 (0.032)**
		*N* = 2000	0.918 (0.013)	0.91 (0.086)	0.918 (0.011)	**0.932 (0.038)**	0.926 (0.043)
		*N* = 5000	0.916 (0.009)	0.906 (0.096)	0.917 (0.004)	0.948 (0.035)	**0.953 (0.031)**
	*p* = 100	*N* = 500	0.917 (0.025)	0.922 (0.033)	0.916 (0.018)	0.92 (0.041)	**0.934 (0.037)**
		*N* = 1000	0.919 (0.016)	0.916 (0.066)	0.917 (0.011)	0.931 (0.047)	**0.935 (0.043)**
		*N* = 2000	0.915 (0.009)	0.918 (0.059)	0.919 (0.011)	0.934 (0.049)	**0.936 (0.031)**
		*N* = 5000	0.916 (0.004)	0.906 (0.088)	0.917 (0.0)	**0.928 (0.039)**	0.928 (0.043)
	*p* = 200	*N* = 500	0.915 (0.019)	0.925 (0.039)	0.916 (0.015)	0.907 (0.037)	**0.929 (0.039)**
		*N* = 1000	0.919 (0.015)	0.918 (0.034)	0.918 (0.014)	**0.934 (0.035)**	0.928 (0.041)
		*N* = 2000	0.915 (0.006)	0.907 (0.059)	0.917 (0.006)	0.919 (0.037)	**0.944 (0.023)**
		*N* = 5000	0.917 (0.006)	0.914 (0.072)	0.917 (0.0)	**0.94 (0.039)**	0.928 (0.037)
	*p* = 500	*N* = 500	0.916 (0.025)	0.925 (0.038)	0.919 (0.015)	0.865 (0.069)	**0.926 (0.047)**
		*N* = 1000	0.916 (0.008)	0.917 (0.041)	0.92 (0.015)	0.908 (0.033)	**0.939 (0.035)**
		*N* = 2000	0.918 (0.011)	0.923 (0.033)	0.917 (0.007)	0.935 (0.031)	**0.938 (0.032)**
		*N* = 5000	0.916 (0.0)	0.915 (0.055)	0.917 (0.0)	0.933 (0.037)	**0.937 (0.031)**
	*p* = 1000	*N* = 500	0.912 (0.02)	0.926 (0.032)	0.919 (0.018)	0.885 (0.061)	**0.949 (0.03)**
		*N* = 1000	0.916 (0.004)	0.926 (0.037)	0.916 (0.007)	**0.927 (0.03)**	0.918 (0.04)
		*N* = 2000	0.917 (0.004)	0.928 (0.039)	0.918 (0.011)	**0.93 (0.02)**	0.928 (0.033)
		*N* = 5000	0.917 (0.004)	0.92 (0.037)	0.917 (0.0)	**0.944 (0.039)**	0.934 (0.033)

**Table A9:** Simulation 1, Scenario 3: mean (SD) of variable-selection measures.

			CNT	CNL	CT	CPH	RSF
			F_1_				
**Σ_1_**	*p* = 50	*N* = 500	**0.629 (0.133)**	0.444 (0.056)	0.537 (0.089)	0.396 (0.027)	0.404 (0.018)
		*N* = 1000	**0.704 (0.13)**	0.526 (0.079)	0.582 (0.102)	0.42 (0.026)	0.412 (0.017)
		*N* = 2000	**0.789 (0.113)**	0.572 (0.121)	0.61 (0.113)	0.459 (0.048)	0.428 (0.024)
		*N* = 5000	**0.879 (0.065)**	0.536 (0.079)	0.644 (0.127)	0.53 (0.061)	0.438 (0.029)
	*p* = 100	*N* = 500	**0.529 (0.136)**	0.263 (0.041)	0.421 (0.081)	0.228 (0.017)	0.228 (0.01)
		*N* = 1000	**0.672 (0.148)**	0.339 (0.063)	0.448 (0.109)	0.263 (0.025)	0.232 (0.013)
		*N* = 2000	**0.774 (0.125)**	0.44 (0.102)	0.484 (0.115)	0.284 (0.03)	0.24 (0.015)
		*N* = 5000	**0.88 (0.057)**	0.621 (0.2)	0.546 (0.113)	0.346 (0.049)	0.26 (0.016)
	*p* = 200	*N* = 500	**0.466 (0.15)**	0.147 (0.029)	0.32 (0.093)	0.119 (0.008)	0.12 (0.006)
		*N* = 1000	**0.626 (0.159)**	0.197 (0.04)	0.337 (0.094)	0.131 (0.014)	0.125 (0.004)
		*N* = 2000	**0.74 (0.153)**	0.273 (0.075)	0.376 (0.095)	0.165 (0.016)	0.127 (0.008)
		*N* = 5000	**0.863 (0.097)**	0.576 (0.129)	0.435 (0.097)	0.215 (0.02)	0.135 (0.01)
	*p* = 500	*N* = 500	**0.351 (0.161)**	0.082 (0.016)	0.214 (0.085)	0.056 (0.009)	0.051 (0.003)
		*N* = 1000	**0.564 (0.184)**	0.086 (0.02)	0.226 (0.086)	0.056 (0.008)	0.051 (0.002)
		*N* = 2000	**0.735 (0.145)**	0.139 (0.031)	0.271 (0.063)	0.069 (0.007)	0.052 (0.003)
		*N* = 5000	**0.863 (0.085)**	0.379 (0.13)	0.322 (0.082)	0.094 (0.008)	0.056 (0.003)
	*p* = 1000	*N* = 500	**0.315 (0.172)**	0.068 (0.015)	0.165 (0.067)	0.036 (0.011)	0.028 (0.001)
		*N* = 1000	**0.474 (0.184)**	0.055 (0.011)	0.181 (0.054)	0.035 (0.007)	0.026 (0.001)
		*N* = 2000	**0.673 (0.194)**	0.064 (0.018)	0.205 (0.058)	0.037 (0.006)	0.027 (0.001)
		*N* = 5000	**0.871 (0.081)**	0.22 (0.081)	0.25 (0.061)	0.049 (0.003)	0.028 (0.001)
**Σ_2_**	*p* = 50	*N* = 500	**0.524 (0.1)**	0.408 (0.038)	0.468 (0.064)	0.406 (0.019)	0.404 (0.014)
		*N* = 1000	**0.622 (0.123)**	0.417 (0.034)	0.473 (0.063)	0.429 (0.026)	0.407 (0.017)
		*N* = 2000	**0.714 (0.126)**	0.421 (0.026)	0.47 (0.059)	0.451 (0.052)	0.421 (0.024)
		*N* = 5000	**0.84 (0.087)**	0.48 (0.05)	0.46 (0.043)	0.49 (0.047)	0.44 (0.02)
	*p* = 100	*N* = 500	**0.363 (0.108)**	0.247 (0.037)	0.314 (0.068)	0.23 (0.015)	0.232 (0.008)
		*N* = 1000	**0.49 (0.134)**	0.257 (0.053)	0.322 (0.056)	0.251 (0.024)	0.233 (0.01)
		*N* = 2000	**0.622 (0.153)**	0.241 (0.044)	0.317 (0.052)	0.258 (0.028)	0.24 (0.01)
		*N* = 5000	**0.8 (0.127)**	0.27 (0.02)	0.31 (0.063)	0.304 (0.049)	0.255 (0.016)
	*p* = 200	*N* = 500	**0.259 (0.106)**	0.133 (0.021)	0.21 (0.062)	0.121 (0.012)	0.121 (0.007)
		*N* = 1000	**0.389 (0.147)**	0.168 (0.039)	0.211 (0.05)	0.128 (0.012)	0.123 (0.005)
		*N* = 2000	**0.55 (0.178)**	0.164 (0.072)	0.223 (0.05)	0.146 (0.018)	0.129 (0.008)
		*N* = 5000	**0.752 (0.151)**	0.141 (0.013)	0.252 (0.056)	0.195 (0.016)	0.142 (0.006)
	*p* = 500	*N* = 500	**0.144 (0.068)**	0.072 (0.019)	0.124 (0.04)	0.052 (0.007)	0.05 (0.002)
		*N* = 1000	**0.272 (0.124)**	0.078 (0.019)	0.14 (0.04)	0.057 (0.008)	0.052 (0.002)
		*N* = 2000	**0.444 (0.193)**	0.115 (0.028)	0.145 (0.04)	0.064 (0.008)	0.054 (0.002)
		*N* = 5000	**0.709 (0.169)**	0.112 (0.101)	0.171 (0.041)	0.084 (0.008)	0.059 (0.004)
	*p* = 1000	*N* = 500	**0.109 (0.056)**	0.049 (0.021)	0.087 (0.036)	0.031 (0.007)	0.027 (0.001)
		*N* = 1000	**0.169 (0.083)**	0.05 (0.012)	0.093 (0.033)	0.035 (0.006)	0.027 (0.001)
		*N* = 2000	**0.351 (0.156)**	0.056 (0.018)	0.103 (0.025)	0.036 (0.004)	0.027 (0.001)
		*N* = 5000	**0.667 (0.179)**	0.134 (0.057)	0.131 (0.028)	0.045 (0.004)	0.029 (0.001)
			**AUC**				
**Σ_1_**	*p* = 50	*N* = 500	0.826 (0.06)	0.838 (0.071)	**0.851 (0.062)**	0.803 (0.073)	0.798 (0.079)
		*N* = 1000	0.887 (0.045)	0.901 (0.079)	**0.903 (0.04)**	0.894 (0.045)	0.898 (0.038)
		*N* = 2000	0.91 (0.033)	0.903 (0.081)	0.911 (0.028)	0.913 (0.037)	**0.918 (0.035)**
		*N* = 5000	0.92 (0.014)	0.917 (0.035)	0.917 (0.028)	0.909 (0.035)	**0.924 (0.039)**
	*p* = 100	*N* = 500	0.81 (0.068)	0.812 (0.07)	**0.856 (0.054)**	0.785 (0.06)	0.769 (0.059)
		*N* = 1000	0.868 (0.047)	0.895 (0.076)	**0.902 (0.034)**	0.891 (0.053)	0.887 (0.044)
		*N* = 2000	0.901 (0.032)	0.898 (0.086)	0.914 (0.027)	**0.92 (0.036)**	0.902 (0.036)
		*N* = 5000	0.917 (0.008)	0.914 (0.066)	0.92 (0.029)	**0.923 (0.04)**	0.922 (0.026)
	*p* = 200	*N* = 500	0.805 (0.052)	0.793 (0.078)	**0.855 (0.059)**	0.728 (0.093)	0.76 (0.058)
		*N* = 1000	0.874 (0.048)	0.889 (0.05)	**0.897 (0.042)**	0.864 (0.054)	0.893 (0.048)
		*N* = 2000	0.896 (0.033)	0.907 (0.066)	**0.917 (0.026)**	0.917 (0.041)	0.907 (0.039)
		*N* = 5000	0.917 (0.014)	0.924 (0.04)	0.916 (0.02)	**0.93 (0.03)**	0.905 (0.032)
	*p* = 500	*N* = 500	0.804 (0.055)	0.816 (0.055)	**0.843 (0.059)**	0.743 (0.082)	0.865 (0.056)
		*N* = 1000	0.856 (0.048)	0.877 (0.056)	**0.889 (0.042)**	0.799 (0.062)	0.864 (0.049)
		*N* = 2000	0.887 (0.036)	**0.916 (0.04)**	0.914 (0.022)	0.897 (0.041)	0.913 (0.043)
		*N* = 5000	0.913 (0.019)	0.921 (0.055)	0.916 (0.016)	**0.923 (0.036)**	0.922 (0.031)
	*p* = 1000	*N* = 500	0.792 (0.046)	0.836 (0.064)	0.828 (0.053)	0.8 (0.087)	**0.904 (0.052)**
		*N* = 1000	0.847 (0.048)	0.877 (0.046)	0.889 (0.036)	0.817 (0.083)	**0.9 (0.05)**
		*N* = 2000	0.888 (0.035)	0.903 (0.032)	**0.913 (0.019)**	0.882 (0.055)	0.888 (0.047)
		*N* = 5000	0.913 (0.015)	**0.929 (0.034)**	0.918 (0.017)	0.91 (0.033)	0.923 (0.036)
**Σ_2_**	*p* = 50	*N* = 500	0.815 (0.07)	0.759 (0.167)	0.844 (0.06)	0.841 (0.067)	**0.856 (0.073)**
		*N* = 1000	0.873 (0.052)	0.885 (0.098)	0.896 (0.043)	**0.918 (0.046)**	0.894 (0.03)
		*N* = 2000	0.906 (0.032)	0.902 (0.064)	0.914 (0.036)	0.904 (0.036)	**0.918 (0.031)**
		*N* = 5000	**0.919 (0.018)**	0.915 (0.035)	0.918 (0.034)	0.916 (0.034)	0.91 (0.032)
	*p* = 100	*N* = 500	0.803 (0.069)	0.769 (0.108)	0.828 (0.069)	0.802 (0.073)	**0.846 (0.062)**
		*N* = 1000	0.867 (0.044)	0.819 (0.149)	0.89 (0.046)	0.903 (0.032)	**0.899 (0.042)**
		*N* = 2000	0.901 (0.03)	0.855 (0.146)	0.912 (0.031)	0.915 (0.039)	**0.921 (0.028)**
		*N* = 5000	0.918 (0.013)	0.918 (0.033)	**0.921 (0.033)**	0.914 (0.029)	0.912 (0.026)
	*p* = 200	*N* = 500	0.776 (0.066)	0.784 (0.088)	**0.822 (0.075)**	0.742 (0.066)	0.798 (0.065)
		*N* = 1000	0.865 (0.052)	0.86 (0.094)	**0.895 (0.042)**	0.88 (0.03)	0.88 (0.054)
		*N* = 2000	0.903 (0.028)	0.773 (0.187)	0.912 (0.031)	**0.92 (0.046)**	0.908 (0.035)
		*N* = 5000	0.915 (0.009)	0.89 (0.104)	0.916 (0.03)	0.917 (0.034)	**0.932 (0.037)**
	*p* = 500	*N* = 500	0.759 (0.071)	0.769 (0.105)	0.805 (0.061)	0.725 (0.091)	**0.807 (0.066)**
		*N* = 1000	0.854 (0.049)	0.875 (0.055)	0.886 (0.046)	0.823 (0.07)	**0.887 (0.033)**
		*N* = 2000	0.895 (0.029)	0.894 (0.084)	0.914 (0.029)	0.903 (0.037)	**0.915 (0.034)**
		*N* = 5000	0.918 (0.012)	0.75 (0.194)	0.916 (0.026)	0.907 (0.03)	**0.922 (0.033)**
	*p* = 1000	*N* = 500	0.739 (0.066)	0.759 (0.133)	0.776 (0.07)	0.74 (0.061)	**0.902 (0.041)**
		*N* = 1000	0.844 (0.051)	0.87 (0.06)	0.882 (0.044)	0.824 (0.055)	**0.9 (0.036)**
		*N* = 2000	0.897 (0.029)	**0.908 (0.041)**	0.907 (0.022)	0.884 (0.036)	0.903 (0.036)
		*N* = 5000	0.915 (0.008)	0.852 (0.166)	0.915 (0.019)	0.904 (0.04)	**0.928 (0.033)**

**Table A10:** Simulation 1, Scenario 4: mean (SD) of variable-selection measures.

			CNT	CNL	CT	CPH	RSF
			F_1_				
**Σ_1_**	*p* = 50	*N* = 500	**0.586 (0.118)**	0.445 (0.048)	0.521 (0.094)	0.414 (0.032)	0.399 (0.019)
		*N* = 1000	**0.706 (0.108)**	0.5 (0.075)	0.545 (0.091)	0.439 (0.03)	0.414 (0.017)
		*N* = 2000	**0.797 (0.114)**	0.591 (0.13)	0.559 (0.101)	0.437 (0.036)	0.409 (0.022)
		*N* = 5000	**0.89 (0.053)**	0.509 (0.058)	0.572 (0.117)	0.537 (0.068)	0.434 (0.024)
	*p* = 100	*N* = 500	**0.52 (0.14)**	0.268 (0.045)	0.387 (0.112)	0.225 (0.016)	0.226 (0.008)
		*N* = 1000	**0.662 (0.144)**	0.317 (0.066)	0.413 (0.108)	0.252 (0.02)	0.225 (0.013)
		*N* = 2000	**0.793 (0.122)**	0.426 (0.116)	0.435 (0.093)	0.264 (0.031)	0.241 (0.012)
		*N* = 5000	**0.867 (0.072)**	0.553 (0.243)	0.465 (0.115)	0.331 (0.052)	0.254 (0.014)
	*p* = 200	*N* = 500	**0.417 (0.147)**	0.136 (0.022)	0.282 (0.085)	0.12 (0.01)	0.122 (0.007)
		*N* = 1000	**0.599 (0.178)**	0.187 (0.046)	0.294 (0.072)	0.14 (0.013)	0.121 (0.005)
		*N* = 2000	**0.744 (0.151)**	0.268 (0.069)	0.315 (0.077)	0.153 (0.013)	0.125 (0.004)
		*N* = 5000	**0.869 (0.057)**	0.559 (0.2)	0.355 (0.08)	0.215 (0.024)	0.135 (0.008)
	*p* = 500	*N* = 500	**0.305 (0.135)**	0.074 (0.018)	0.186 (0.076)	0.059 (0.011)	0.051 (0.002)
		*N* = 1000	**0.489 (0.198)**	0.079 (0.018)	0.208 (0.075)	0.056 (0.008)	0.051 (0.002)
		*N* = 2000	**0.703 (0.182)**	0.134 (0.031)	0.232 (0.072)	0.064 (0.011)	0.051 (0.003)
		*N* = 5000	**0.876 (0.057)**	0.38 (0.146)	0.251 (0.056)	0.092 (0.006)	0.056 (0.003)
	*p* = 1000	*N* = 500	**0.238 (0.146)**	0.061 (0.018)	0.132 (0.055)	0.034 (0.01)	0.028 (0.001)
		*N* = 1000	**0.402 (0.18)**	0.053 (0.011)	0.15 (0.047)	0.036 (0.008)	0.027 (0.001)
		*N* = 2000	**0.613 (0.205)**	0.061 (0.02)	0.181 (0.054)	0.036 (0.005)	0.027 (0.001)
		*N* = 5000	**0.857 (0.1)**	0.214 (0.072)	0.193 (0.039)	0.047 (0.003)	0.028 (0.001)
**Σ_2_**	*p* = 50	*N* = 500	**0.506 (0.097)**	0.41 (0.035)	0.458 (0.052)	0.409 (0.031)	0.408 (0.016)
		*N* = 1000	**0.567 (0.121)**	0.416 (0.03)	0.462 (0.052)	0.431 (0.036)	0.413 (0.023)
		*N* = 2000	**0.682 (0.148)**	0.429 (0.023)	0.454 (0.047)	0.436 (0.036)	0.425 (0.018)
		*N* = 5000	**0.823 (0.102)**	0.477 (0.048)	0.46 (0.035)	0.5 (0.053)	0.439 (0.027)
	*p* = 100	*N* = 500	**0.382 (0.134)**	0.242 (0.036)	0.292 (0.055)	0.234 (0.015)	0.225 (0.01)
		*N* = 1000	**0.503 (0.151)**	0.243 (0.046)	0.294 (0.044)	0.242 (0.021)	0.233 (0.008)
		*N* = 2000	**0.634 (0.158)**	0.24 (0.014)	0.312 (0.049)	0.271 (0.029)	0.237 (0.012)
		*N* = 5000	**0.79 (0.112)**	0.271 (0.022)	0.29 (0.044)	0.321 (0.046)	0.258 (0.015)
	*p* = 200	*N* = 500	**0.232 (0.101)**	0.133 (0.022)	0.194 (0.053)	0.123 (0.011)	0.119 (0.006)
		*N* = 1000	**0.364 (0.131)**	0.172 (0.042)	0.203 (0.047)	0.137 (0.012)	0.123 (0.007)
		*N* = 2000	**0.526 (0.162)**	0.148 (0.063)	0.204 (0.04)	0.147 (0.018)	0.13 (0.004)
		*N* = 5000	**0.755 (0.143)**	0.142 (0.011)	0.211 (0.05)	0.185 (0.026)	0.134 (0.009)
	*p* = 500	*N* = 500	**0.136 (0.065)**	0.066 (0.018)	0.115 (0.035)	0.052 (0.006)	0.049 (0.004)
		*N* = 1000	**0.222 (0.129)**	0.069 (0.018)	0.114 (0.037)	0.057 (0.008)	0.05 (0.003)
		*N* = 2000	**0.399 (0.162)**	0.108 (0.031)	0.134 (0.041)	0.062 (0.007)	0.053 (0.002)
		*N* = 5000	**0.679 (0.171)**	0.081 (0.072)	0.147 (0.028)	0.085 (0.005)	0.057 (0.003)
	*p* = 1000	*N* = 500	**0.091 (0.054)**	0.046 (0.018)	0.084 (0.026)	0.031 (0.008)	0.028 (0.001)
		*N* = 1000	**0.178 (0.114)**	0.045 (0.012)	0.08 (0.031)	0.033 (0.008)	0.026 (0.001)
		*N* = 2000	**0.281 (0.121)**	0.052 (0.016)	0.096 (0.029)	0.032 (0.007)	0.027 (0.001)
		*N* = 5000	**0.575 (0.213)**	0.118 (0.059)	0.106 (0.022)	0.044 (0.003)	0.029 (0.001)
			**AUC**				
**Σ_1_**	*p* = 50	*N* = 500	0.796 (0.068)	0.826 (0.086)	**0.829 (0.067)**	0.791 (0.072)	0.791 (0.068)
		*N* = 1000	0.87 (0.051)	**0.902 (0.089)**	0.884 (0.047)	0.888 (0.058)	0.88 (0.051)
		*N* = 2000	0.906 (0.024)	**0.93 (0.063)**	0.913 (0.033)	0.914 (0.037)	0.901 (0.047)
		*N* = 5000	0.921 (0.017)	**0.94 (0.036)**	0.914 (0.031)	0.915 (0.034)	0.931 (0.039)
	*p* = 100	*N* = 500	0.801 (0.063)	0.804 (0.086)	**0.834 (0.069)**	0.774 (0.081)	0.775 (0.071)
		*N* = 1000	0.861 (0.047)	0.893 (0.082)	**0.896 (0.049)**	0.892 (0.052)	0.878 (0.051)
		*N* = 2000	0.902 (0.027)	0.911 (0.099)	0.906 (0.024)	0.903 (0.034)	**0.919 (0.034)**
		*N* = 5000	0.916 (0.006)	**0.933 (0.085)**	0.91 (0.024)	0.911 (0.039)	0.931 (0.031)
	*p* = 200	*N* = 500	0.777 (0.065)	0.781 (0.07)	**0.827 (0.055)**	0.696 (0.093)	0.762 (0.083)
		*N* = 1000	0.861 (0.045)	0.878 (0.057)	**0.886 (0.04)**	0.877 (0.038)	0.836 (0.062)
		*N* = 2000	0.892 (0.033)	**0.923 (0.079)**	0.913 (0.028)	0.912 (0.035)	0.907 (0.027)
		*N* = 5000	0.916 (0.012)	0.927 (0.105)	0.916 (0.023)	**0.93 (0.029)**	0.929 (0.032)
	*p* = 500	*N* = 500	0.784 (0.065)	0.786 (0.07)	**0.82 (0.053)**	0.705 (0.089)	0.812 (0.065)
		*N* = 1000	0.846 (0.05)	0.871 (0.045)	**0.881 (0.046)**	0.757 (0.069)	0.827 (0.06)
		*N* = 2000	0.891 (0.035)	**0.909 (0.037)**	0.908 (0.023)	0.892 (0.048)	0.884 (0.042)
		*N* = 5000	0.914 (0.009)	**0.939 (0.097)**	0.914 (0.016)	0.915 (0.036)	0.939 (0.037)
	*p* = 1000	*N* = 500	0.77 (0.055)	0.801 (0.09)	0.805 (0.063)	0.789 (0.08)	**0.902 (0.039)**
		*N* = 1000	0.84 (0.047)	0.863 (0.054)	0.879 (0.047)	0.813 (0.068)	**0.904 (0.048)**
		*N* = 2000	0.882 (0.037)	0.906 (0.039)	**0.912 (0.027)**	0.861 (0.053)	0.896 (0.047)
		*N* = 5000	0.914 (0.011)	**0.948 (0.038)**	0.917 (0.016)	0.912 (0.03)	0.918 (0.035)
**Σ_2_**	*p* = 50	*N* = 500	0.792 (0.077)	0.771 (0.159)	0.838 (0.071)	0.84 (0.066)	**0.842 (0.055)**
		*N* = 1000	0.874 (0.046)	0.881 (0.093)	0.898 (0.047)	**0.898 (0.038)**	0.886 (0.042)
		*N* = 2000	0.906 (0.038)	**0.927 (0.049)**	0.922 (0.037)	0.913 (0.041)	0.922 (0.043)
		*N* = 5000	0.92 (0.017)	**0.936 (0.04)**	0.933 (0.038)	0.922 (0.046)	0.929 (0.038)
	*p* = 100	*N* = 500	0.778 (0.071)	0.762 (0.12)	**0.817 (0.067)**	0.803 (0.069)	0.795 (0.073)
		*N* = 1000	0.871 (0.049)	0.781 (0.181)	0.887 (0.046)	0.885 (0.054)	**0.901 (0.04)**
		*N* = 2000	0.897 (0.032)	0.89 (0.124)	**0.92 (0.032)**	0.917 (0.039)	0.913 (0.028)
		*N* = 5000	0.917 (0.016)	0.933 (0.034)	0.927 (0.037)	0.923 (0.044)	**0.938 (0.029)**
	*p* = 200	*N* = 500	0.761 (0.068)	0.755 (0.101)	**0.798 (0.072)**	0.748 (0.054)	0.781 (0.08)
		*N* = 1000	0.851 (0.054)	0.84 (0.122)	**0.885 (0.043)**	0.88 (0.051)	0.88 (0.056)
		*N* = 2000	0.903 (0.03)	0.708 (0.212)	0.917 (0.036)	**0.922 (0.036)**	0.918 (0.032)
		*N* = 5000	0.915 (0.01)	**0.931 (0.054)**	0.92 (0.029)	0.929 (0.034)	0.912 (0.036)
	*p* = 500	*N* = 500	0.746 (0.075)	0.744 (0.112)	0.79 (0.071)	0.691 (0.077)	**0.796 (0.097)**
		*N* = 1000	0.848 (0.051)	0.859 (0.073)	**0.879 (0.048)**	0.82 (0.07)	0.849 (0.08)
		*N* = 2000	0.9 (0.028)	0.88 (0.087)	0.911 (0.03)	**0.916 (0.033)**	0.915 (0.033)
		*N* = 5000	0.914 (0.01)	0.746 (0.198)	**0.915 (0.025)**	0.911 (0.021)	0.915 (0.041)
	*p* = 1000	*N* = 500	0.721 (0.067)	0.756 (0.11)	0.79 (0.066)	0.745 (0.056)	**0.893 (0.046)**
		*N* = 1000	0.834 (0.052)	0.85 (0.083)	0.862 (0.057)	0.818 (0.066)	**0.89 (0.044)**
		*N* = 2000	0.895 (0.033)	**0.91 (0.038)**	0.906 (0.031)	0.884 (0.063)	0.908 (0.048)
		*N* = 5000	0.915 (0.01)	0.852 (0.156)	0.912 (0.02)	0.909 (0.04)	**0.936 (0.029)**

**Table A11:** Simulation 2, Scenario 1: mean (SD) of C-index.

			CNT	CNL	CT	CPH	RSF
**Σ_1_**	*p* = 50	*N* = 500	0.882 (0.011)	0.884 (0.012)	**0.886 (0.009)**	0.879 (0.015)	0.879 (0.012)
		*N* = 1000	0.889 (0.008)	0.888 (0.007)	**0.89 (0.007)**	0.886 (0.008)	0.889 (0.007)
		*N* = 2000	0.891 (0.005)	0.89 (0.004)	**0.893 (0.004)**	0.89 (0.004)	0.891 (0.004)
		*N* = 5000	0.891 (0.004)	0.891 (0.003)	**0.892 (0.003)**	0.858 (0.016)	0.89 (0.003)
	*p* = 100	*N* = 500	0.881 (0.011)	0.879 (0.011)	**0.887 (0.011)**	0.858 (0.016)	0.855 (0.017)
		*N* = 1000	0.888 (0.007)	0.888 (0.007)	**0.89 (0.007)**	0.88 (0.008)	0.875 (0.007)
		*N* = 2000	0.891 (0.005)	0.89 (0.005)	**0.892 (0.004)**	0.886 (0.006)	0.885 (0.005)
		*N* = 5000	0.891 (0.003)	0.892 (0.003)	**0.892 (0.003)**	0.89 (0.004)	0.89 (0.002)
	*p* = 200	*N* = 500	0.877 (0.013)	0.868 (0.012)	**0.882 (0.011)**	0.751 (0.037)	0.831 (0.016)
		*N* = 1000	0.886 (0.008)	0.883 (0.008)	**0.889 (0.007)**	0.865 (0.01)	0.86 (0.009)
		*N* = 2000	0.891 (0.005)	0.889 (0.004)	**0.891 (0.004)**	0.883 (0.004)	0.879 (0.005)
		*N* = 5000	0.892 (0.003)	0.891 (0.003)	**0.892 (0.003)**	0.891 (0.003)	0.888 (0.003)
	*p* = 500	*N* = 500	0.874 (0.013)	0.864 (0.012)	**0.883 (0.012)**	0.637 (0.032)	0.812 (0.013)
		*N* = 1000	0.883 (0.008)	0.877 (0.007)	**0.888 (0.007)**	0.752 (0.014)	0.816 (0.013)
		*N* = 2000	0.889 (0.005)	0.886 (0.005)	**0.891 (0.004)**	0.861 (0.006)	0.85 (0.007)
		*N* = 5000	0.891 (0.003)	0.89 (0.003)	**0.891 (0.003)**	0.886 (0.003)	0.878 (0.004)
	*p* = 1000	*N* = 500	0.873 (0.016)	0.866 (0.012)	**0.88 (0.013)**	0.624 (0.02)	0.802 (0.018)
		*N* = 1000	0.881 (0.01)	0.875 (0.008)	**0.887 (0.007)**	0.664 (0.014)	0.809 (0.014)
		*N* = 2000	0.888 (0.005)	0.883 (0.005)	**0.89 (0.005)**	0.808 (0.01)	0.821 (0.009)
		*N* = 5000	0.891 (0.003)	0.89 (0.003)	**0.892 (0.003)**	0.878 (0.003)	0.859 (0.004)
**Σ_2_**	*p* = 50	*N* = 500	0.836 (0.014)	0.832 (0.045)	**0.842 (0.014)**	0.836 (0.017)	0.837 (0.015)
		*N* = 1000	**0.851 (0.01)**	0.848 (0.01)	0.85 (0.01)	0.847 (0.007)	0.846 (0.01)
		*N* = 2000	0.853 (0.006)	0.85 (0.035)	**0.853 (0.006)**	0.85 (0.006)	0.85 (0.006)
		*N* = 5000	0.854 (0.003)	0.851 (0.035)	0.854 (0.004)	**0.855 (0.003)**	0.854 (0.004)
	*p* = 100	*N* = 500	0.831 (0.017)	0.823 (0.019)	**0.838 (0.015)**	0.792 (0.025)	0.805 (0.018)
		*N* = 1000	0.847 (0.011)	0.843 (0.035)	**0.849 (0.011)**	0.835 (0.009)	0.833 (0.013)
		*N* = 2000	0.852 (0.006)	0.848 (0.033)	**0.853 (0.006)**	0.847 (0.006)	0.847 (0.007)
		*N* = 5000	0.854 (0.004)	0.842 (0.067)	**0.854 (0.004)**	0.854 (0.004)	0.851 (0.003)
	*p* = 200	*N* = 500	0.828 (0.017)	0.803 (0.021)	**0.834 (0.015)**	0.672 (0.035)	0.758 (0.019)
		*N* = 1000	0.842 (0.012)	0.839 (0.01)	**0.844 (0.011)**	0.803 (0.013)	0.807 (0.014)
		*N* = 2000	0.852 (0.007)	0.849 (0.007)	**0.853 (0.006)**	0.841 (0.006)	0.836 (0.008)
		*N* = 5000	**0.855 (0.004)**	0.846 (0.052)	0.854 (0.005)	0.851 (0.003)	0.848 (0.004)
	*p* = 500	*N* = 500	0.825 (0.016)	0.796 (0.018)	**0.832 (0.015)**	0.568 (0.034)	0.701 (0.013)
		*N* = 1000	0.838 (0.012)	0.826 (0.011)	**0.841 (0.011)**	0.654 (0.029)	0.744 (0.014)
		*N* = 2000	0.848 (0.009)	0.844 (0.007)	**0.85 (0.006)**	0.806 (0.011)	0.789 (0.005)
		*N* = 5000	**0.855 (0.004)**	0.848 (0.035)	0.853 (0.004)	0.845 (0.004)	0.835 (0.004)
	*p* = 1000	*N* = 500	0.82 (0.022)	0.796 (0.02)	**0.829 (0.014)**	0.568 (0.032)	0.687 (0.026)
		*N* = 1000	**0.838 (0.011)**	0.819 (0.011)	0.837 (0.011)	0.597 (0.021)	0.706 (0.013)
		*N* = 2000	0.844 (0.009)	0.84 (0.007)	**0.848 (0.007)**	0.722 (0.012)	0.742 (0.011)
		*N* = 5000	**0.854 (0.005)**	0.852 (0.004)	0.853 (0.005)	0.832 (0.005)	0.806 (0.004)

**Table A12:** Simulation 2, Scenario 2: mean (SD) of C-index.

			CNT	CNL	CT	CPH	RSF
**Σ_1_**	*p* = 50	*N* = 500	0.801 (0.018)	0.799 (0.019)	**0.803 (0.017)**	0.796 (0.019)	0.785 (0.017)
		*N* = 1000	0.805 (0.013)	0.809 (0.012)	**0.809 (0.011)**	0.808 (0.013)	0.8 (0.008)
		*N* = 2000	0.811 (0.009)	**0.814 (0.008)**	0.811 (0.008)	0.81 (0.005)	0.808 (0.009)
		*N* = 5000	0.812 (0.005)	**0.855 (0.016)**	0.813 (0.005)	0.814 (0.005)	0.813 (0.004)
	*p* = 100	*N* = 500	0.798 (0.018)	0.787 (0.018)	**0.803 (0.015)**	0.75 (0.019)	0.763 (0.018)
		*N* = 1000	0.804 (0.012)	0.804 (0.011)	**0.808 (0.011)**	0.795 (0.01)	0.793 (0.011)
		*N* = 2000	0.81 (0.008)	**0.813 (0.007)**	0.811 (0.006)	0.809 (0.005)	0.805 (0.007)
		*N* = 5000	0.813 (0.005)	**0.814 (0.005)**	0.813 (0.004)	0.811 (0.005)	0.81 (0.005)
	*p* = 200	*N* = 500	0.795 (0.02)	0.766 (0.022)	**0.803 (0.017)**	0.667 (0.025)	0.73 (0.019)
		*N* = 1000	0.805 (0.011)	0.796 (0.013)	**0.807 (0.012)**	0.771 (0.011)	0.763 (0.013)
		*N* = 2000	**0.811 (0.008)**	0.809 (0.007)	0.81 (0.008)	0.801 (0.008)	0.791 (0.005)
		*N* = 5000	0.813 (0.005)	**0.814 (0.005)**	0.813 (0.005)	0.809 (0.003)	0.806 (0.005)
	*p* = 500	*N* = 500	0.792 (0.02)	0.756 (0.023)	**0.8 (0.017)**	0.602 (0.031)	0.706 (0.019)
		*N* = 1000	0.804 (0.012)	0.782 (0.012)	**0.808 (0.011)**	0.669 (0.011)	0.715 (0.012)
		*N* = 2000	0.81 (0.008)	0.802 (0.008)	**0.811 (0.007)**	0.773 (0.008)	0.746 (0.008)
		*N* = 5000	0.812 (0.004)	0.812 (0.004)	**0.814 (0.005)**	0.806 (0.006)	0.788 (0.006)
	*p* = 1000	*N* = 500	0.79 (0.019)	0.759 (0.026)	**0.799 (0.016)**	0.585 (0.027)	0.705 (0.02)
		*N* = 1000	0.802 (0.013)	0.774 (0.015)	**0.809 (0.011)**	0.62 (0.014)	0.706 (0.013)
		*N* = 2000	0.809 (0.009)	0.796 (0.009)	**0.811 (0.007)**	0.712 (0.009)	0.714 (0.009)
		*N* = 5000	0.812 (0.005)	0.81 (0.005)	**0.813 (0.004)**	0.794 (0.003)	0.762 (0.005)
**Σ_2_**	*p* = 50	*N* = 500	0.743 (0.022)	0.731 (0.043)	**0.754 (0.02)**	0.734 (0.02)	0.729 (0.018)
		*N* = 1000	0.755 (0.013)	0.744 (0.06)	**0.759 (0.014)**	0.757 (0.013)	0.751 (0.014)
		*N* = 2000	0.76 (0.01)	0.731 (0.083)	**0.762 (0.01)**	0.762 (0.01)	0.762 (0.007)
		*N* = 5000	0.765 (0.006)	**0.809 (0.049)**	0.767 (0.006)	0.766 (0.005)	0.765 (0.006)
	*p* = 100	*N* = 500	0.741 (0.024)	0.721 (0.036)	**0.75 (0.018)**	0.682 (0.028)	0.701 (0.021)
		*N* = 1000	0.754 (0.013)	0.74 (0.039)	**0.76 (0.012)**	0.736 (0.016)	0.739 (0.018)
		*N* = 2000	0.762 (0.01)	0.745 (0.061)	**0.764 (0.008)**	0.759 (0.007)	0.755 (0.007)
		*N* = 5000	0.765 (0.006)	0.744 (0.076)	**0.767 (0.005)**	0.765 (0.004)	0.764 (0.005)
	*p* = 200	*N* = 500	0.736 (0.024)	0.688 (0.035)	**0.745 (0.02)**	0.597 (0.026)	0.654 (0.028)
		*N* = 1000	0.754 (0.014)	0.735 (0.035)	**0.759 (0.013)**	0.701 (0.027)	0.7 (0.018)
		*N* = 2000	0.76 (0.011)	0.754 (0.026)	**0.764 (0.007)**	0.748 (0.008)	0.736 (0.011)
		*N* = 5000	0.766 (0.006)	0.743 (0.072)	**0.767 (0.006)**	0.764 (0.005)	0.757 (0.005)
	*p* = 500	*N* = 500	0.731 (0.024)	0.676 (0.028)	**0.733 (0.021)**	0.557 (0.03)	0.616 (0.024)
		*N* = 1000	0.75 (0.013)	0.715 (0.02)	**0.757 (0.013)**	0.601 (0.03)	0.646 (0.02)
		*N* = 2000	0.758 (0.011)	0.75 (0.011)	**0.765 (0.008)**	0.715 (0.012)	0.682 (0.009)
		*N* = 5000	0.766 (0.006)	0.748 (0.062)	**0.767 (0.006)**	0.754 (0.004)	0.735 (0.006)
	*p* = 1000	*N* = 500	**0.721 (0.034)**	0.678 (0.024)	0.719 (0.029)	0.542 (0.028)	0.616 (0.02)
		*N* = 1000	0.751 (0.013)	0.706 (0.021)	**0.753 (0.015)**	0.564 (0.021)	0.627 (0.015)
		*N* = 2000	0.756 (0.011)	0.738 (0.011)	**0.763 (0.008)**	0.633 (0.028)	0.639 (0.011)
		*N* = 5000	0.765 (0.007)	0.762 (0.006)	**0.766 (0.005)**	0.738 (0.006)	0.698 (0.005)

**Table A13:** Simulation 2, Scenario 3: mean (SD) of C-index.

			CNT	CNL	CT	CPH	RSF
**Σ_1_**	*p* = 50	*N* = 500	**0.648 (0.027)**	0.629 (0.032)	0.639 (0.024)	0.628 (0.028)	0.618 (0.024)
		*N* = 1000	0.657 (0.014)	**0.658 (0.053)**	0.65 (0.019)	0.647 (0.015)	0.644 (0.016)
		*N* = 2000	0.667 (0.013)	**0.733 (0.092)**	0.659 (0.011)	0.663 (0.011)	0.66 (0.012)
		*N* = 5000	0.668 (0.007)	**0.861 (0.038)**	0.665 (0.007)	0.667 (0.008)	0.667 (0.007)
	*p* = 100	*N* = 500	**0.648 (0.026)**	0.604 (0.037)	0.628 (0.028)	0.587 (0.021)	0.6 (0.028)
		*N* = 1000	**0.655 (0.015)**	0.639 (0.029)	0.64 (0.019)	0.625 (0.014)	0.625 (0.018)
		*N* = 2000	0.665 (0.012)	**0.669 (0.058)**	0.651 (0.012)	0.654 (0.013)	0.643 (0.008)
		*N* = 5000	0.667 (0.007)	**0.742 (0.098)**	0.661 (0.007)	0.666 (0.009)	0.66 (0.006)
	*p* = 200	*N* = 500	**0.638 (0.029)**	0.579 (0.03)	0.611 (0.028)	0.555 (0.019)	0.567 (0.021)
		*N* = 1000	**0.655 (0.018)**	0.621 (0.028)	0.631 (0.017)	0.593 (0.026)	0.59 (0.02)
		*N* = 2000	**0.665 (0.011)**	0.653 (0.039)	0.644 (0.014)	0.638 (0.014)	0.627 (0.012)
		*N* = 5000	0.667 (0.007)	**0.672 (0.067)**	0.655 (0.007)	0.658 (0.008)	0.65 (0.008)
	*p* = 500	*N* = 500	**0.627 (0.029)**	0.574 (0.03)	0.598 (0.03)	0.531 (0.023)	0.564 (0.023)
		*N* = 1000	**0.651 (0.019)**	0.589 (0.025)	0.623 (0.024)	0.55 (0.022)	0.569 (0.022)
		*N* = 2000	**0.661 (0.012)**	0.635 (0.019)	0.631 (0.014)	0.598 (0.016)	0.591 (0.014)
		*N* = 5000	0.667 (0.006)	**0.669 (0.045)**	0.644 (0.009)	0.645 (0.007)	0.624 (0.007)
	*p* = 1000	*N* = 500	**0.619 (0.032)**	0.575 (0.03)	0.592 (0.032)	0.535 (0.026)	0.568 (0.019)
		*N* = 1000	**0.649 (0.02)**	0.583 (0.019)	0.612 (0.024)	0.537 (0.014)	0.558 (0.016)
		*N* = 2000	**0.66 (0.012)**	0.612 (0.023)	0.623 (0.017)	0.569 (0.016)	0.57 (0.013)
		*N* = 5000	**0.667 (0.006)**	0.658 (0.008)	0.636 (0.011)	0.626 (0.008)	0.596 (0.008)
**Σ_2_**	*p* = 50	*N* = 500	**0.598 (0.028)**	0.565 (0.049)	0.594 (0.027)	0.593 (0.027)	0.571 (0.021)
		*N* = 1000	**0.623 (0.02)**	0.608 (0.032)	0.611 (0.018)	0.619 (0.014)	0.613 (0.021)
		*N* = 2000	0.636 (0.012)	**0.642 (0.037)**	0.625 (0.012)	0.626 (0.01)	0.626 (0.013)
		*N* = 5000	0.643 (0.008)	**0.815 (0.05)**	0.641 (0.008)	0.639 (0.006)	0.64 (0.009)
	*p* = 100	*N* = 500	**0.591 (0.032)**	0.563 (0.035)	0.579 (0.029)	0.576 (0.029)	0.578 (0.023)
		*N* = 1000	**0.621 (0.021)**	0.556 (0.047)	0.595 (0.017)	0.591 (0.018)	0.593 (0.013)
		*N* = 2000	**0.633 (0.012)**	0.601 (0.025)	0.609 (0.012)	0.618 (0.012)	0.613 (0.011)
		*N* = 5000	0.642 (0.007)	**0.649 (0.016)**	0.631 (0.008)	0.634 (0.008)	0.632 (0.008)
	*p* = 200	*N* = 500	**0.573 (0.027)**	0.544 (0.033)	0.566 (0.031)	0.54 (0.026)	0.55 (0.024)
		*N* = 1000	**0.604 (0.021)**	0.565 (0.039)	0.586 (0.02)	0.57 (0.017)	0.564 (0.018)
		*N* = 2000	**0.627 (0.015)**	0.546 (0.05)	0.596 (0.014)	0.6 (0.017)	0.592 (0.015)
		*N* = 5000	**0.64 (0.007)**	0.617 (0.008)	0.615 (0.009)	0.626 (0.009)	0.62 (0.007)
	*p* = 500	*N* = 500	**0.553 (0.031)**	0.532 (0.028)	0.548 (0.03)	0.526 (0.019)	0.538 (0.023)
		*N* = 1000	**0.589 (0.026)**	0.556 (0.023)	0.569 (0.021)	0.527 (0.017)	0.543 (0.019)
		*N* = 2000	**0.624 (0.015)**	0.596 (0.027)	0.584 (0.014)	0.567 (0.016)	0.555 (0.011)
		*N* = 5000	**0.639 (0.008)**	0.546 (0.05)	0.593 (0.01)	0.615 (0.008)	0.593 (0.008)
	*p* = 1000	*N* = 500	0.541 (0.032)	0.528 (0.031)	**0.543 (0.029)**	0.519 (0.022)	0.536 (0.031)
		*N* = 1000	**0.574 (0.027)**	0.549 (0.023)	0.559 (0.022)	0.521 (0.02)	0.541 (0.017)
		*N* = 2000	**0.615 (0.016)**	0.576 (0.019)	0.572 (0.016)	0.538 (0.02)	0.545 (0.013)
		*N* = 5000	**0.637 (0.008)**	0.599 (0.051)	0.584 (0.011)	0.592 (0.006)	0.567 (0.009)

**Table A14:** Simulation 2, Scenario 4: mean (SD) of C-index.

			CNT	CNL	CT	CPH	RSF
**Σ_1_**	*p* = 50	*N* = 500	**0.632 (0.026)**	0.616 (0.035)	0.619 (0.023)	0.609 (0.019)	0.611 (0.024)
		*N* = 1000	**0.643 (0.018)**	0.626 (0.062)	0.635 (0.016)	0.634 (0.016)	0.635 (0.021)
		*N* = 2000	0.65 (0.01)	**0.739 (0.101)**	0.641 (0.012)	0.642 (0.011)	0.645 (0.012)
		*N* = 5000	0.654 (0.007)	**0.852 (0.027)**	0.652 (0.007)	0.651 (0.008)	0.652 (0.006)
	*p* = 100	*N* = 500	**0.628 (0.026)**	0.584 (0.038)	0.611 (0.024)	0.581 (0.03)	0.573 (0.021)
		*N* = 1000	**0.644 (0.016)**	0.604 (0.054)	0.621 (0.021)	0.615 (0.016)	0.608 (0.018)
		*N* = 2000	**0.652 (0.012)**	0.628 (0.057)	0.634 (0.014)	0.636 (0.009)	0.63 (0.013)
		*N* = 5000	0.655 (0.007)	**0.731 (0.121)**	0.647 (0.006)	0.651 (0.006)	0.645 (0.007)
	*p* = 200	*N* = 500	**0.623 (0.027)**	0.569 (0.032)	0.596 (0.03)	0.544 (0.025)	0.566 (0.032)
		*N* = 1000	**0.643 (0.016)**	0.608 (0.026)	0.611 (0.019)	0.587 (0.017)	0.584 (0.02)
		*N* = 2000	**0.646 (0.012)**	0.628 (0.043)	0.622 (0.014)	0.62 (0.011)	0.608 (0.012)
		*N* = 5000	**0.654 (0.007)**	0.642 (0.062)	0.637 (0.008)	0.644 (0.006)	0.639 (0.009)
	*p* = 500	*N* = 500	**0.606 (0.033)**	0.556 (0.028)	0.584 (0.031)	0.531 (0.024)	0.546 (0.027)
		*N* = 1000	**0.635 (0.019)**	0.576 (0.026)	0.596 (0.02)	0.541 (0.025)	0.555 (0.013)
		*N* = 2000	**0.646 (0.012)**	0.618 (0.018)	0.612 (0.017)	0.59 (0.016)	0.576 (0.016)
		*N* = 5000	**0.653 (0.007)**	0.639 (0.056)	0.625 (0.009)	0.632 (0.007)	0.61 (0.006)
	*p* = 1000	*N* = 500	**0.597 (0.034)**	0.556 (0.031)	0.568 (0.033)	0.528 (0.022)	0.552 (0.024)
		*N* = 1000	**0.63 (0.021)**	0.569 (0.02)	0.589 (0.023)	0.527 (0.017)	0.557 (0.017)
		*N* = 2000	**0.645 (0.013)**	0.596 (0.02)	0.605 (0.017)	0.561 (0.011)	0.555 (0.01)
		*N* = 5000	**0.652 (0.008)**	0.642 (0.012)	0.618 (0.01)	0.611 (0.007)	0.583 (0.008)
**Σ_2_**	*p* = 50	*N* = 500	**0.587 (0.028)**	0.552 (0.04)	0.582 (0.026)	0.575 (0.023)	0.576 (0.025)
		*N* = 1000	0.609 (0.018)	0.595 (0.029)	0.598 (0.02)	**0.61 (0.021)**	0.602 (0.018)
		*N* = 2000	0.623 (0.011)	**0.629 (0.032)**	0.614 (0.011)	0.62 (0.012)	0.614 (0.011)
		*N* = 5000	0.629 (0.007)	**0.796 (0.044)**	0.627 (0.007)	0.629 (0.007)	0.626 (0.008)
	*p* = 100	*N* = 500	**0.574 (0.031)**	0.549 (0.035)	0.57 (0.028)	0.54 (0.025)	0.551 (0.02)
		*N* = 1000	**0.603 (0.019)**	0.548 (0.04)	0.582 (0.019)	0.583 (0.012)	0.579 (0.011)
		*N* = 2000	**0.621 (0.013)**	0.595 (0.017)	0.595 (0.012)	0.599 (0.011)	0.603 (0.012)
		*N* = 5000	0.628 (0.007)	**0.63 (0.015)**	0.618 (0.008)	0.624 (0.007)	0.618 (0.005)
	*p* = 200	*N* = 500	**0.556 (0.03)**	0.539 (0.029)	0.554 (0.028)	0.523 (0.022)	0.537 (0.025)
		*N* = 1000	**0.592 (0.021)**	0.556 (0.033)	0.568 (0.021)	0.558 (0.02)	0.555 (0.019)
		*N* = 2000	**0.614 (0.016)**	0.547 (0.043)	0.58 (0.012)	0.58 (0.012)	0.582 (0.012)
		*N* = 5000	**0.627 (0.008)**	0.603 (0.01)	0.602 (0.009)	0.613 (0.008)	0.606 (0.008)
	*p* = 500	*N* = 500	**0.544 (0.028)**	0.525 (0.031)	0.541 (0.025)	0.531 (0.022)	0.534 (0.023)
		*N* = 1000	**0.58 (0.027)**	0.544 (0.027)	0.554 (0.022)	0.528 (0.015)	0.535 (0.018)
		*N* = 2000	**0.602 (0.018)**	0.573 (0.029)	0.568 (0.016)	0.558 (0.01)	0.552 (0.015)
		*N* = 5000	**0.623 (0.008)**	0.547 (0.042)	0.58 (0.009)	0.6 (0.007)	0.583 (0.009)
	*p* = 1000	*N* = 500	**0.533 (0.025)**	0.522 (0.027)	0.533 (0.028)	0.514 (0.024)	0.533 (0.018)
		*N* = 1000	**0.565 (0.025)**	0.536 (0.023)	0.546 (0.018)	0.519 (0.015)	0.53 (0.013)
		*N* = 2000	**0.598 (0.018)**	0.562 (0.021)	0.555 (0.018)	0.525 (0.019)	0.541 (0.011)
		*N* = 5000	**0.62 (0.01)**	0.581 (0.051)	0.571 (0.011)	0.583 (0.008)	0.555 (0.007)

**Table A15:** Simulation 2, Scenario 1: mean (SD) of variable-selection measures.

			CNT	CNL	CT	CPH	RSF
			F_1_				
**Σ_1_**	*p* = 50	*N* = 500	0.808 (0.115)	0.573 (0.064)	**0.903 (0.069)**	0.429 (0.048)	0.4 (0.022)
		*N* = 1000	0.914 (0.104)	0.693 (0.081)	**0.967 (0.042)**	0.478 (0.053)	0.443 (0.031)
		*N* = 2000	0.969 (0.049)	0.845 (0.077)	**0.995 (0.019)**	0.56 (0.065)	0.491 (0.022)
		*N* = 5000	0.996 (0.029)	0.962 (0.058)	**1.0 (0.005)**	0.787 (0.096)	0.651 (0.053)
	*p* = 100	*N* = 500	0.774 (0.158)	0.408 (0.039)	**0.865 (0.093)**	0.252 (0.018)	0.224 (0.011)
		*N* = 1000	0.886 (0.086)	0.527 (0.057)	**0.938 (0.066)**	0.301 (0.031)	0.255 (0.013)
		*N* = 2000	0.968 (0.044)	0.735 (0.078)	**0.992 (0.022)**	0.416 (0.05)	0.307 (0.024)
		*N* = 5000	0.995 (0.032)	0.96 (0.044)	**1.0 (0.005)**	0.647 (0.07)	0.438 (0.047)
	*p* = 200	*N* = 500	0.748 (0.16)	0.273 (0.042)	**0.843 (0.112)**	0.123 (0.011)	0.12 (0.004)
		*N* = 1000	0.858 (0.111)	0.376 (0.048)	**0.918 (0.078)**	0.169 (0.011)	0.137 (0.005)
		*N* = 2000	0.939 (0.071)	0.589 (0.09)	**0.984 (0.032)**	0.246 (0.018)	0.168 (0.013)
		*N* = 5000	0.997 (0.014)	0.923 (0.058)	**1.0 (0.0)**	0.517 (0.064)	0.272 (0.023)
	*p* = 500	*N* = 500	0.77 (0.148)	0.208 (0.031)	**0.808 (0.128)**	0.061 (0.007)	0.057 (0.003)
		*N* = 1000	0.826 (0.155)	0.246 (0.029)	**0.9 (0.092)**	0.066 (0.003)	0.057 (0.002)
		*N* = 2000	0.921 (0.101)	0.402 (0.066)	**0.983 (0.037)**	0.103 (0.007)	0.067 (0.003)
		*N* = 5000	0.996 (0.013)	0.843 (0.085)	**1.0 (0.0)**	0.261 (0.029)	0.111 (0.01)
	*p* = 1000	*N* = 500	0.756 (0.147)	0.219 (0.032)	**0.845 (0.112)**	0.044 (0.003)	0.035 (0.002)
		*N* = 1000	0.871 (0.142)	0.221 (0.026)	**0.919 (0.085)**	0.041 (0.001)	0.035 (0.002)
		*N* = 2000	0.921 (0.12)	0.32 (0.058)	**0.983 (0.033)**	0.049 (0.002)	0.035 (0.002)
		*N* = 5000	0.993 (0.021)	0.776 (0.089)	**1.0 (0.005)**	0.135 (0.012)	0.051 (0.002)
**Σ_2_**	*p* = 50	*N* = 500	0.736 (0.125)	0.518 (0.07)	**0.786 (0.094)**	0.426 (0.043)	0.407 (0.024)
		*N* = 1000	0.858 (0.116)	0.643 (0.093)	**0.882 (0.066)**	0.475 (0.046)	0.461 (0.032)
		*N* = 2000	0.946 (0.091)	0.764 (0.128)	**0.968 (0.041)**	0.581 (0.09)	0.534 (0.031)
		*N* = 5000	0.997 (0.021)	0.937 (0.101)	**0.997 (0.012)**	0.802 (0.099)	0.73 (0.078)
	*p* = 100	*N* = 500	0.695 (0.122)	0.345 (0.048)	**0.728 (0.108)**	0.236 (0.019)	0.229 (0.01)
		*N* = 1000	0.815 (0.108)	0.463 (0.066)	**0.83 (0.099)**	0.301 (0.032)	0.263 (0.013)
		*N* = 2000	**0.931 (0.058)**	0.649 (0.083)	0.931 (0.065)	0.386 (0.043)	0.334 (0.03)
		*N* = 5000	**0.999 (0.007)**	0.893 (0.145)	0.997 (0.012)	0.643 (0.089)	0.504 (0.051)
	*p* = 200	*N* = 500	0.692 (0.116)	0.2 (0.04)	**0.746 (0.116)**	0.115 (0.01)	0.117 (0.006)
		*N* = 1000	0.776 (0.122)	0.311 (0.032)	**0.804 (0.116)**	0.158 (0.008)	0.134 (0.005)
		*N* = 2000	0.884 (0.086)	0.487 (0.054)	**0.917 (0.064)**	0.231 (0.016)	0.18 (0.015)
		*N* = 5000	**0.996 (0.013)**	0.85 (0.13)	0.995 (0.017)	0.473 (0.045)	0.317 (0.032)
	*p* = 500	*N* = 500	0.639 (0.129)	0.149 (0.021)	**0.728 (0.085)**	0.048 (0.009)	0.05 (0.002)
		*N* = 1000	0.79 (0.119)	0.184 (0.024)	**0.837 (0.12)**	0.057 (0.007)	0.054 (0.002)
		*N* = 2000	0.856 (0.125)	0.31 (0.034)	**0.889 (0.087)**	0.091 (0.005)	0.065 (0.002)
		*N* = 5000	0.989 (0.03)	0.739 (0.11)	**0.994 (0.018)**	0.234 (0.026)	0.125 (0.01)
	*p* = 1000	*N* = 500	0.575 (0.151)	0.148 (0.025)	**0.673 (0.103)**	0.035 (0.01)	0.029 (0.002)
		*N* = 1000	0.755 (0.122)	0.153 (0.021)	**0.834 (0.104)**	0.037 (0.006)	0.028 (0.001)
		*N* = 2000	0.871 (0.149)	0.227 (0.029)	**0.877 (0.107)**	0.042 (0.002)	0.032 (0.001)
		*N* = 5000	0.967 (0.084)	0.678 (0.099)	**0.993 (0.023)**	0.119 (0.01)	0.054 (0.003)
			**AUC**				
**Σ_1_**	*p* = 50	*N* = 500	0.972 (0.041)	0.984 (0.025)	**0.992 (0.019)**	0.98 (0.028)	0.975 (0.028)
		*N* = 1000	0.997 (0.011)	0.999 (0.004)	0.999 (0.007)	0.999 (0.002)	**1.0 (0.0)**
		*N* = 2000	1.0 (0.0)	1.0 (0.0)	**1.0 (0.0)**	1.0 (0.0)	1.0 (0.0)
		*N* = 5000	1.0 (0.0)	1.0 (0.0)	**1.0 (0.0)**	1.0 (0.0)	1.0 (0.0)
	*p* = 100	*N* = 500	0.971 (0.039)	0.986 (0.019)	**0.988 (0.026)**	0.976 (0.018)	0.971 (0.037)
		*N* = 1000	0.997 (0.01)	**0.999 (0.003)**	0.998 (0.012)	0.999 (0.003)	0.999 (0.003)
		*N* = 2000	1.0 (0.0)	1.0 (0.001)	**1.0 (0.0)**	1.0 (0.0)	1.0 (0.0)
		*N* = 5000	1.0 (0.0)	1.0 (0.0)	**1.0 (0.0)**	1.0 (0.0)	1.0 (0.0)
	*p* = 200	*N* = 500	0.946 (0.062)	**0.982 (0.02)**	0.97 (0.039)	0.923 (0.042)	0.964 (0.037)
		*N* = 1000	0.987 (0.028)	**0.999 (0.004)**	0.997 (0.012)	0.999 (0.003)	0.995 (0.009)
		*N* = 2000	1.0 (0.0)	**1.0 (0.0)**	1.0 (0.0)	1.0 (0.0)	1.0 (0.0)
		*N* = 5000	1.0 (0.0)	**1.0 (0.0)**	1.0 (0.0)	1.0 (0.0)	1.0 (0.0)
	*p* = 500	*N* = 500	0.936 (0.054)	0.99 (0.013)	0.954 (0.052)	0.924 (0.044)	**0.991 (0.012)**
		*N* = 1000	0.976 (0.039)	**0.999 (0.002)**	0.992 (0.018)	0.981 (0.02)	0.999 (0.001)
		*N* = 2000	0.999 (0.007)	**1.0 (0.0)**	1.0 (0.0)	1.0 (0.0)	1.0 (0.0)
		*N* = 5000	1.0 (0.0)	**1.0 (0.0)**	1.0 (0.0)	1.0 (0.0)	1.0 (0.0)
	*p* = 1000	*N* = 500	0.913 (0.069)	0.988 (0.018)	0.93 (0.054)	0.948 (0.046)	**0.999 (0.002)**
		*N* = 1000	0.969 (0.046)	**1.0 (0.001)**	0.985 (0.029)	0.983 (0.015)	0.999 (0.001)
		*N* = 2000	0.993 (0.017)	**1.0 (0.0)**	1.0 (0.0)	0.999 (0.001)	1.0 (0.0)
		*N* = 5000	1.0 (0.0)	**1.0 (0.0)**	1.0 (0.0)	1.0 (0.0)	1.0 (0.0)
**Σ_2_**	*p* = 50	*N* = 500	0.955 (0.054)	0.977 (0.042)	0.967 (0.047)	**0.993 (0.013)**	0.987 (0.013)
		*N* = 1000	0.998 (0.011)	**1.0 (0.002)**	0.997 (0.012)	1.0 (0.001)	1.0 (0.001)
		*N* = 2000	1.0 (0.0)	0.996 (0.035)	**1.0 (0.0)**	1.0 (0.0)	1.0 (0.0)
		*N* = 5000	1.0 (0.0)	0.995 (0.054)	**1.0 (0.0)**	1.0 (0.0)	1.0 (0.0)
	*p* = 100	*N* = 500	0.915 (0.076)	**0.978 (0.027)**	0.929 (0.069)	0.976 (0.023)	0.963 (0.041)
		*N* = 1000	0.977 (0.045)	0.993 (0.06)	0.995 (0.024)	0.999 (0.002)	**1.0 (0.001)**
		*N* = 2000	1.0 (0.0)	0.996 (0.042)	**1.0 (0.0)**	1.0 (0.0)	1.0 (0.0)
		*N* = 5000	1.0 (0.0)	0.986 (0.082)	**1.0 (0.0)**	1.0 (0.0)	1.0 (0.0)
	*p* = 200	*N* = 500	0.877 (0.079)	**0.968 (0.036)**	0.887 (0.078)	0.882 (0.061)	0.941 (0.046)
		*N* = 1000	0.944 (0.073)	**0.999 (0.003)**	0.97 (0.058)	0.996 (0.009)	0.999 (0.001)
		*N* = 2000	1.0 (0.0)	1.0 (0.0)	**1.0 (0.0)**	1.0 (0.0)	1.0 (0.0)
		*N* = 5000	1.0 (0.0)	0.99 (0.07)	**1.0 (0.0)**	1.0 (0.0)	1.0 (0.0)
	*p* = 500	*N* = 500	0.868 (0.069)	**0.96 (0.035)**	0.851 (0.06)	0.821 (0.072)	0.949 (0.043)
		*N* = 1000	0.919 (0.075)	**0.998 (0.007)**	0.925 (0.072)	0.931 (0.041)	0.993 (0.009)
		*N* = 2000	0.985 (0.034)	**1.0 (0.0)**	0.998 (0.01)	1.0 (0.0)	1.0 (0.0)
		*N* = 5000	1.0 (0.0)	0.996 (0.038)	**1.0 (0.0)**	1.0 (0.0)	1.0 (0.0)
	*p* = 1000	*N* = 500	0.858 (0.074)	0.963 (0.034)	0.841 (0.055)	0.862 (0.075)	**0.976 (0.021)**
		*N* = 1000	0.908 (0.086)	**0.997 (0.008)**	0.897 (0.073)	0.937 (0.047)	0.988 (0.019)
		*N* = 2000	0.968 (0.056)	**1.0 (0.0)**	0.982 (0.039)	0.998 (0.004)	0.999 (0.001)
		*N* = 5000	1.0 (0.0)	**1.0 (0.0)**	1.0 (0.0)	1.0 (0.0)	1.0 (0.0)

**Table A16:** Simulation 2, Scenario 2: mean (SD) of variable-selection measures.

			CNT	CNL	CT	CPH	RSF
			F_1_				
**Σ_1_**	*p* = 50	*N* = 500	0.682 (0.127)	0.504 (0.057)	**0.737 (0.106)**	0.437 (0.027)	0.424 (0.024)
		*N* = 1000	0.753 (0.117)	0.593 (0.075)	**0.801 (0.103)**	0.469 (0.035)	0.442 (0.028)
		*N* = 2000	**0.83 (0.114)**	0.683 (0.089)	0.818 (0.111)	0.53 (0.04)	0.463 (0.035)
		*N* = 5000	**0.842 (0.127)**	0.598 (0.096)	0.84 (0.092)	0.646 (0.086)	0.544 (0.043)
	*p* = 100	*N* = 500	0.674 (0.108)	0.329 (0.042)	**0.719 (0.11)**	0.256 (0.017)	0.235 (0.014)
		*N* = 1000	0.765 (0.091)	0.43 (0.05)	**0.807 (0.119)**	0.284 (0.032)	0.252 (0.012)
		*N* = 2000	0.847 (0.054)	0.544 (0.089)	**0.882 (0.05)**	0.344 (0.045)	0.275 (0.017)
		*N* = 5000	**0.899 (0.022)**	0.775 (0.093)	0.89 (0.058)	0.463 (0.077)	0.334 (0.029)
	*p* = 200	*N* = 500	0.645 (0.128)	0.197 (0.032)	**0.705 (0.097)**	0.131 (0.011)	0.128 (0.007)
		*N* = 1000	0.758 (0.097)	0.27 (0.037)	**0.821 (0.079)**	0.16 (0.012)	0.132 (0.007)
		*N* = 2000	0.844 (0.06)	0.396 (0.052)	**0.881 (0.047)**	0.199 (0.016)	0.151 (0.009)
		*N* = 5000	0.898 (0.033)	0.677 (0.103)	**0.905 (0.027)**	0.316 (0.047)	0.181 (0.013)
	*p* = 500	*N* = 500	0.576 (0.132)	0.13 (0.024)	**0.642 (0.116)**	0.062 (0.008)	0.056 (0.003)
		*N* = 1000	0.713 (0.107)	0.149 (0.02)	**0.788 (0.082)**	0.068 (0.004)	0.057 (0.002)
		*N* = 2000	0.814 (0.072)	0.227 (0.034)	**0.878 (0.037)**	0.086 (0.005)	0.061 (0.003)
		*N* = 5000	0.877 (0.076)	0.529 (0.072)	**0.906 (0.012)**	0.153 (0.014)	0.073 (0.007)
	*p* = 1000	*N* = 500	0.566 (0.14)	0.119 (0.026)	**0.595 (0.108)**	0.043 (0.01)	0.033 (0.002)
		*N* = 1000	0.693 (0.124)	0.115 (0.017)	**0.773 (0.09)**	0.043 (0.003)	0.031 (0.002)
		*N* = 2000	0.814 (0.091)	0.153 (0.022)	**0.87 (0.042)**	0.044 (0.003)	0.032 (0.001)
		*N* = 5000	0.879 (0.081)	0.395 (0.064)	**0.906 (0.011)**	0.078 (0.006)	0.037 (0.002)
**Σ_2_**	*p* = 50	*N* = 500	0.613 (0.11)	0.475 (0.059)	**0.67 (0.101)**	0.443 (0.03)	0.419 (0.023)
		*N* = 1000	0.708 (0.101)	0.537 (0.084)	**0.752 (0.118)**	0.466 (0.035)	0.456 (0.038)
		*N* = 2000	0.791 (0.112)	0.583 (0.134)	**0.858 (0.069)**	0.518 (0.051)	0.489 (0.035)
		*N* = 5000	0.856 (0.129)	0.629 (0.11)	**0.896 (0.057)**	0.636 (0.072)	0.567 (0.043)
	*p* = 100	*N* = 500	0.579 (0.11)	0.306 (0.043)	**0.593 (0.113)**	0.239 (0.021)	0.235 (0.016)
		*N* = 1000	0.688 (0.086)	0.373 (0.058)	**0.735 (0.086)**	0.285 (0.028)	0.257 (0.015)
		*N* = 2000	0.784 (0.097)	0.467 (0.104)	**0.855 (0.047)**	0.332 (0.031)	0.284 (0.018)
		*N* = 5000	0.89 (0.062)	0.624 (0.205)	**0.907 (0.008)**	0.449 (0.092)	0.355 (0.03)
	*p* = 200	*N* = 500	**0.521 (0.129)**	0.165 (0.028)	0.489 (0.105)	0.124 (0.013)	0.126 (0.008)
		*N* = 1000	0.627 (0.097)	0.24 (0.031)	**0.681 (0.098)**	0.154 (0.02)	0.135 (0.008)
		*N* = 2000	0.763 (0.103)	0.333 (0.05)	**0.825 (0.06)**	0.196 (0.018)	0.151 (0.007)
		*N* = 5000	0.874 (0.077)	0.525 (0.2)	**0.908 (0.008)**	0.306 (0.041)	0.197 (0.016)
	*p* = 500	*N* = 500	**0.442 (0.147)**	0.109 (0.021)	0.368 (0.106)	0.056 (0.009)	0.054 (0.002)
		*N* = 1000	**0.567 (0.121)**	0.123 (0.018)	0.549 (0.111)	0.059 (0.008)	0.055 (0.004)
		*N* = 2000	0.722 (0.095)	0.182 (0.025)	**0.797 (0.078)**	0.082 (0.005)	0.061 (0.004)
		*N* = 5000	0.858 (0.122)	0.393 (0.111)	**0.902 (0.019)**	0.149 (0.012)	0.08 (0.005)
	*p* = 1000	*N* = 500	**0.342 (0.14)**	0.091 (0.019)	0.279 (0.107)	0.038 (0.009)	0.03 (0.002)
		*N* = 1000	**0.559 (0.115)**	0.092 (0.015)	0.501 (0.124)	0.037 (0.006)	0.029 (0.002)
		*N* = 2000	0.693 (0.095)	0.116 (0.015)	**0.728 (0.106)**	0.04 (0.005)	0.031 (0.002)
		*N* = 5000	0.816 (0.18)	0.308 (0.054)	**0.903 (0.015)**	0.071 (0.004)	0.037 (0.003)
			**AUC**				
**Σ_1_**	*p* = 50	*N* = 500	0.831 (0.049)	0.85 (0.056)	0.861 (0.045)	**0.87 (0.053)**	0.842 (0.05)
		*N* = 1000	0.87 (0.046)	0.908 (0.048)	0.89 (0.04)	**0.919 (0.041)**	0.917 (0.056)
		*N* = 2000	0.897 (0.03)	0.926 (0.044)	0.911 (0.025)	**0.931 (0.04)**	0.923 (0.045)
		*N* = 5000	0.913 (0.019)	**0.963 (0.037)**	0.918 (0.02)	0.937 (0.041)	0.941 (0.034)
	*p* = 100	*N* = 500	0.827 (0.054)	**0.854 (0.058)**	0.842 (0.048)	0.834 (0.068)	0.816 (0.059)
		*N* = 1000	0.859 (0.043)	0.906 (0.044)	0.89 (0.035)	**0.92 (0.035)**	0.894 (0.048)
		*N* = 2000	0.889 (0.033)	0.926 (0.042)	0.905 (0.021)	0.936 (0.032)	**0.937 (0.035)**
		*N* = 5000	0.911 (0.015)	**0.936 (0.042)**	0.917 (0.009)	0.93 (0.038)	0.933 (0.039)
	*p* = 200	*N* = 500	0.812 (0.051)	**0.843 (0.05)**	0.834 (0.048)	0.8 (0.047)	0.837 (0.055)
		*N* = 1000	0.863 (0.045)	**0.899 (0.04)**	0.883 (0.036)	0.898 (0.046)	0.897 (0.054)
		*N* = 2000	0.89 (0.026)	0.917 (0.037)	0.903 (0.022)	0.927 (0.037)	**0.942 (0.029)**
		*N* = 5000	0.912 (0.013)	0.927 (0.039)	0.916 (0.008)	**0.94 (0.038)**	0.92 (0.039)
	*p* = 500	*N* = 500	0.805 (0.055)	0.858 (0.052)	0.822 (0.049)	0.784 (0.072)	**0.874 (0.041)**
		*N* = 1000	0.836 (0.05)	0.893 (0.049)	0.869 (0.045)	0.854 (0.038)	**0.903 (0.034)**
		*N* = 2000	0.883 (0.034)	0.921 (0.036)	0.901 (0.027)	**0.931 (0.033)**	0.923 (0.04)
		*N* = 5000	0.911 (0.016)	0.927 (0.04)	0.915 (0.008)	**0.928 (0.038)**	0.924 (0.039)
	*p* = 1000	*N* = 500	0.799 (0.053)	0.877 (0.051)	0.823 (0.051)	0.864 (0.062)	**0.921 (0.038)**
		*N* = 1000	0.832 (0.053)	0.907 (0.048)	0.865 (0.04)	0.888 (0.041)	**0.912 (0.051)**
		*N* = 2000	0.884 (0.033)	0.93 (0.034)	0.9 (0.027)	0.898 (0.042)	**0.935 (0.042)**
		*N* = 5000	0.911 (0.017)	0.919 (0.034)	0.915 (0.008)	0.922 (0.034)	**0.934 (0.028)**
**Σ_2_**	*p* = 50	*N* = 500	0.801 (0.06)	0.845 (0.083)	0.824 (0.057)	**0.887 (0.052)**	0.864 (0.047)
		*N* = 1000	0.822 (0.057)	0.886 (0.098)	0.87 (0.049)	0.912 (0.045)	**0.927 (0.047)**
		*N* = 2000	0.879 (0.049)	0.865 (0.17)	0.899 (0.028)	0.938 (0.044)	**0.949 (0.041)**
		*N* = 5000	0.915 (0.021)	0.95 (0.085)	0.916 (0.012)	0.943 (0.033)	**0.954 (0.034)**
	*p* = 100	*N* = 500	0.788 (0.056)	**0.846 (0.069)**	0.818 (0.053)	0.805 (0.055)	0.828 (0.07)
		*N* = 1000	0.817 (0.06)	0.89 (0.065)	0.856 (0.05)	**0.913 (0.026)**	0.904 (0.044)
		*N* = 2000	0.862 (0.06)	0.895 (0.088)	0.895 (0.026)	**0.932 (0.035)**	0.929 (0.038)
		*N* = 5000	0.911 (0.013)	0.896 (0.112)	0.916 (0.004)	0.933 (0.045)	**0.944 (0.032)**
	*p* = 200	*N* = 500	0.777 (0.063)	**0.826 (0.06)**	0.813 (0.053)	0.765 (0.066)	0.792 (0.069)
		*N* = 1000	0.803 (0.056)	0.888 (0.065)	0.855 (0.046)	**0.893 (0.054)**	0.877 (0.04)
		*N* = 2000	0.864 (0.061)	0.912 (0.047)	0.895 (0.033)	0.927 (0.045)	**0.936 (0.032)**
		*N* = 5000	0.913 (0.016)	0.886 (0.109)	0.917 (0.0)	0.921 (0.034)	**0.939 (0.034)**
	*p* = 500	*N* = 500	0.765 (0.051)	**0.828 (0.065)**	0.795 (0.051)	0.74 (0.087)	0.826 (0.059)
		*N* = 1000	0.786 (0.061)	**0.891 (0.048)**	0.854 (0.045)	0.791 (0.066)	0.856 (0.042)
		*N* = 2000	0.839 (0.064)	0.917 (0.037)	0.895 (0.033)	0.924 (0.035)	**0.927 (0.038)**
		*N* = 5000	0.914 (0.013)	0.89 (0.104)	0.915 (0.007)	**0.943 (0.033)**	0.932 (0.038)
	*p* = 1000	*N* = 500	0.758 (0.044)	0.822 (0.061)	0.78 (0.047)	0.765 (0.084)	**0.904 (0.051)**
		*N* = 1000	0.779 (0.05)	**0.893 (0.046)**	0.844 (0.054)	0.802 (0.062)	0.89 (0.042)
		*N* = 2000	0.819 (0.062)	**0.918 (0.038)**	0.893 (0.03)	0.89 (0.053)	0.916 (0.049)
		*N* = 5000	0.909 (0.028)	0.917 (0.035)	0.917 (0.0)	0.921 (0.033)	**0.923 (0.041)**

**Table A17:** Simulation 2, Scenario 3: mean (SD) of variable-selection measures.

			CNT	CNL	CT	CPH	RSF
			F_1_				
**Σ_1_**	*p* = 50	*N* = 500	**0.536 (0.098)**	0.417 (0.045)	0.455 (0.078)	0.399 (0.032)	0.393 (0.019)
		*N* = 1000	**0.607 (0.104)**	0.439 (0.063)	0.475 (0.074)	0.408 (0.038)	0.4 (0.024)
		*N* = 2000	**0.649 (0.082)**	0.471 (0.083)	0.483 (0.069)	0.439 (0.051)	0.415 (0.026)
		*N* = 5000	**0.712 (0.083)**	0.467 (0.044)	0.472 (0.072)	0.49 (0.065)	0.434 (0.028)
	*p* = 100	*N* = 500	**0.435 (0.108)**	0.242 (0.037)	0.324 (0.079)	0.219 (0.014)	0.217 (0.016)
		*N* = 1000	**0.494 (0.104)**	0.288 (0.057)	0.339 (0.074)	0.231 (0.023)	0.22 (0.013)
		*N* = 2000	**0.593 (0.102)**	0.333 (0.089)	0.334 (0.057)	0.259 (0.03)	0.236 (0.014)
		*N* = 5000	**0.681 (0.097)**	0.379 (0.132)	0.366 (0.082)	0.331 (0.046)	0.247 (0.016)
	*p* = 200	*N* = 500	**0.362 (0.121)**	0.13 (0.022)	0.223 (0.063)	0.116 (0.005)	0.116 (0.008)
		*N* = 1000	**0.429 (0.126)**	0.157 (0.033)	0.225 (0.062)	0.125 (0.011)	0.119 (0.007)
		*N* = 2000	**0.543 (0.125)**	0.227 (0.05)	0.236 (0.064)	0.149 (0.02)	0.124 (0.008)
		*N* = 5000	**0.698 (0.076)**	0.309 (0.138)	0.284 (0.05)	0.199 (0.028)	0.131 (0.009)
	*p* = 500	*N* = 500	**0.281 (0.129)**	0.068 (0.015)	0.141 (0.049)	0.054 (0.009)	0.05 (0.002)
		*N* = 1000	**0.399 (0.14)**	0.068 (0.016)	0.153 (0.048)	0.053 (0.007)	0.05 (0.004)
		*N* = 2000	**0.491 (0.137)**	0.103 (0.024)	0.157 (0.043)	0.061 (0.007)	0.051 (0.003)
		*N* = 5000	**0.637 (0.116)**	0.218 (0.082)	0.18 (0.036)	0.086 (0.007)	0.056 (0.003)
	*p* = 1000	*N* = 500	**0.211 (0.121)**	0.05 (0.015)	0.109 (0.04)	0.032 (0.009)	0.026 (0.002)
		*N* = 1000	**0.325 (0.116)**	0.044 (0.01)	0.113 (0.044)	0.032 (0.007)	0.026 (0.002)
		*N* = 2000	**0.449 (0.146)**	0.052 (0.015)	0.117 (0.035)	0.032 (0.004)	0.025 (0.003)
		*N* = 5000	**0.623 (0.115)**	0.146 (0.054)	0.136 (0.032)	0.047 (0.006)	0.028 (0.002)
**Σ_2_**	*p* = 50	*N* = 500	**0.46 (0.086)**	0.4 (0.038)	0.415 (0.05)	0.416 (0.029)	0.384 (0.021)
		*N* = 1000	**0.518 (0.087)**	0.406 (0.029)	0.424 (0.048)	0.415 (0.029)	0.399 (0.026)
		*N* = 2000	**0.588 (0.095)**	0.416 (0.024)	0.425 (0.042)	0.428 (0.041)	0.418 (0.027)
		*N* = 5000	**0.633 (0.113)**	0.498 (0.053)	0.461 (0.042)	0.494 (0.061)	0.438 (0.029)
	*p* = 100	*N* = 500	**0.328 (0.1)**	0.233 (0.031)	0.271 (0.05)	0.23 (0.014)	0.223 (0.009)
		*N* = 1000	**0.413 (0.119)**	0.232 (0.033)	0.271 (0.041)	0.24 (0.018)	0.228 (0.013)
		*N* = 2000	**0.469 (0.098)**	0.233 (0.014)	0.271 (0.034)	0.26 (0.028)	0.229 (0.015)
		*N* = 5000	**0.586 (0.092)**	0.255 (0.017)	0.265 (0.026)	0.312 (0.051)	0.256 (0.017)
	*p* = 200	*N* = 500	**0.219 (0.083)**	0.126 (0.018)	0.17 (0.047)	0.121 (0.008)	0.114 (0.007)
		*N* = 1000	**0.285 (0.111)**	0.145 (0.033)	0.182 (0.04)	0.12 (0.01)	0.12 (0.009)
		*N* = 2000	**0.348 (0.102)**	0.131 (0.038)	0.173 (0.031)	0.139 (0.012)	0.125 (0.011)
		*N* = 5000	**0.51 (0.118)**	0.137 (0.008)	0.186 (0.039)	0.174 (0.028)	0.138 (0.012)
	*p* = 500	*N* = 500	**0.132 (0.074)**	0.06 (0.014)	0.102 (0.029)	0.05 (0.006)	0.049 (0.003)
		*N* = 1000	**0.189 (0.099)**	0.064 (0.015)	0.096 (0.03)	0.051 (0.005)	0.05 (0.003)
		*N* = 2000	**0.284 (0.104)**	0.093 (0.024)	0.107 (0.023)	0.06 (0.008)	0.051 (0.002)
		*N* = 5000	**0.408 (0.13)**	0.069 (0.053)	0.116 (0.028)	0.08 (0.009)	0.057 (0.004)
	*p* = 1000	*N* = 500	**0.086 (0.049)**	0.039 (0.016)	0.07 (0.024)	0.028 (0.005)	0.026 (0.002)
		*N* = 1000	**0.12 (0.07)**	0.042 (0.012)	0.061 (0.019)	0.028 (0.005)	0.026 (0.002)
		*N* = 2000	**0.206 (0.09)**	0.046 (0.013)	0.066 (0.019)	0.03 (0.006)	0.026 (0.002)
		*N* = 5000	**0.328 (0.116)**	0.099 (0.048)	0.086 (0.022)	0.044 (0.003)	0.028 (0.002)
			**AUC**				
**Σ_1_**	*p* = 50	*N* = 500	0.726 (0.062)	0.721 (0.087)	**0.727 (0.078)**	0.721 (0.097)	0.713 (0.088)
		*N* = 1000	0.767 (0.058)	**0.787 (0.098)**	0.786 (0.072)	0.755 (0.083)	0.739 (0.053)
		*N* = 2000	0.784 (0.052)	**0.837 (0.079)**	0.813 (0.066)	0.818 (0.086)	0.821 (0.082)
		*N* = 5000	0.812 (0.05)	**0.915 (0.046)**	0.859 (0.059)	0.866 (0.054)	0.883 (0.054)
	*p* = 100	*N* = 500	0.718 (0.059)	0.699 (0.092)	**0.731 (0.065)**	0.667 (0.068)	0.66 (0.089)
		*N* = 1000	0.748 (0.049)	0.772 (0.07)	**0.783 (0.07)**	0.748 (0.047)	0.749 (0.072)
		*N* = 2000	0.782 (0.054)	0.819 (0.104)	**0.823 (0.061)**	0.815 (0.066)	0.804 (0.046)
		*N* = 5000	0.815 (0.05)	**0.879 (0.106)**	0.852 (0.068)	0.862 (0.075)	0.877 (0.066)
	*p* = 200	*N* = 500	0.712 (0.052)	0.707 (0.074)	**0.732 (0.062)**	0.614 (0.066)	0.655 (0.07)
		*N* = 1000	0.755 (0.044)	0.761 (0.069)	**0.779 (0.063)**	0.718 (0.075)	0.743 (0.057)
		*N* = 2000	0.772 (0.049)	**0.804 (0.082)**	0.802 (0.063)	0.804 (0.066)	0.768 (0.063)
		*N* = 5000	0.804 (0.044)	0.859 (0.106)	0.863 (0.055)	**0.877 (0.049)**	0.857 (0.054)
	*p* = 500	*N* = 500	0.723 (0.059)	0.714 (0.078)	**0.735 (0.057)**	0.649 (0.086)	0.735 (0.063)
		*N* = 1000	0.742 (0.042)	0.764 (0.067)	**0.772 (0.057)**	0.705 (0.086)	0.741 (0.062)
		*N* = 2000	0.77 (0.046)	**0.803 (0.069)**	0.796 (0.058)	0.78 (0.07)	0.786 (0.066)
		*N* = 5000	0.798 (0.048)	0.855 (0.09)	0.843 (0.051)	**0.862 (0.052)**	0.852 (0.043)
	*p* = 1000	*N* = 500	0.705 (0.052)	0.734 (0.08)	0.739 (0.055)	0.708 (0.09)	**0.812 (0.076)**
		*N* = 1000	0.749 (0.042)	0.75 (0.062)	0.762 (0.056)	0.692 (0.078)	**0.782 (0.058)**
		*N* = 2000	0.762 (0.038)	**0.803 (0.061)**	0.782 (0.053)	0.737 (0.071)	0.783 (0.088)
		*N* = 5000	0.791 (0.036)	0.851 (0.063)	0.846 (0.056)	**0.863 (0.065)**	0.845 (0.069)
**Σ_2_**	*p* = 50	*N* = 500	0.708 (0.071)	0.675 (0.127)	0.709 (0.073)	**0.734 (0.081)**	0.7 (0.068)
		*N* = 1000	0.75 (0.061)	0.784 (0.088)	0.764 (0.077)	0.781 (0.072)	**0.79 (0.087)**
		*N* = 2000	0.783 (0.054)	**0.847 (0.066)**	0.807 (0.078)	0.797 (0.078)	0.836 (0.069)
		*N* = 5000	0.81 (0.059)	**0.911 (0.041)**	0.888 (0.051)	0.883 (0.052)	0.887 (0.047)
	*p* = 100	*N* = 500	0.693 (0.063)	0.669 (0.107)	0.716 (0.073)	**0.717 (0.083)**	0.698 (0.064)
		*N* = 1000	0.746 (0.067)	0.69 (0.136)	0.766 (0.073)	**0.804 (0.063)**	0.77 (0.078)
		*N* = 2000	0.768 (0.054)	**0.808 (0.099)**	0.802 (0.066)	0.797 (0.071)	0.792 (0.081)
		*N* = 5000	0.801 (0.053)	0.881 (0.048)	0.866 (0.065)	0.883 (0.059)	**0.883 (0.045)**
	*p* = 200	*N* = 500	0.685 (0.063)	0.676 (0.094)	0.705 (0.074)	**0.707 (0.063)**	0.655 (0.066)
		*N* = 1000	0.752 (0.058)	0.706 (0.119)	**0.78 (0.064)**	0.726 (0.062)	0.761 (0.067)
		*N* = 2000	0.767 (0.055)	0.653 (0.167)	0.788 (0.062)	**0.808 (0.059)**	0.807 (0.067)
		*N* = 5000	0.803 (0.051)	0.877 (0.053)	0.864 (0.05)	0.872 (0.057)	**0.893 (0.038)**
	*p* = 500	*N* = 500	0.681 (0.059)	0.661 (0.095)	0.712 (0.065)	0.642 (0.055)	**0.736 (0.091)**
		*N* = 1000	0.727 (0.05)	0.734 (0.068)	0.751 (0.067)	0.71 (0.062)	**0.763 (0.074)**
		*N* = 2000	0.761 (0.049)	0.771 (0.079)	**0.799 (0.056)**	0.771 (0.076)	0.784 (0.061)
		*N* = 5000	0.788 (0.046)	0.674 (0.172)	**0.845 (0.057)**	0.827 (0.064)	0.877 (0.077)
	*p* = 1000	*N* = 500	0.665 (0.059)	0.66 (0.114)	0.705 (0.06)	0.642 (0.053)	**0.793 (0.055)**
		*N* = 1000	0.724 (0.048)	0.752 (0.073)	0.734 (0.05)	0.684 (0.055)	**0.779 (0.068)**
		*N* = 2000	0.754 (0.04)	0.779 (0.059)	0.776 (0.062)	0.733 (0.09)	**0.785 (0.075)**
		*N* = 5000	0.794 (0.05)	0.76 (0.157)	0.841 (0.054)	**0.863 (0.046)**	0.83 (0.064)

**Table A18:** Simulation 2, Scenario 4: mean (SD) of variable selection measures.

			CNT	CNL	CT	CPH	RSF
			F_1_				
**Σ_1_**	*p* = 50	*N* = 500	**0.497 (0.098)**	0.417 (0.054)	0.427 (0.059)	0.394 (0.028)	0.385 (0.026)
		*N* = 1000	**0.588 (0.095)**	0.443 (0.076)	0.443 (0.055)	0.413 (0.031)	0.396 (0.019)
		*N* = 2000	**0.633 (0.097)**	0.436 (0.065)	0.45 (0.063)	0.451 (0.035)	0.412 (0.023)
		*N* = 5000	**0.706 (0.095)**	0.455 (0.035)	0.462 (0.057)	0.5 (0.07)	0.429 (0.02)
	*p* = 100	*N* = 500	**0.404 (0.118)**	0.236 (0.029)	0.297 (0.065)	0.223 (0.019)	0.215 (0.014)
		*N* = 1000	**0.497 (0.128)**	0.263 (0.054)	0.298 (0.062)	0.239 (0.023)	0.228 (0.01)
		*N* = 2000	**0.573 (0.119)**	0.309 (0.089)	0.313 (0.06)	0.255 (0.032)	0.232 (0.01)
		*N* = 5000	**0.677 (0.104)**	0.305 (0.106)	0.322 (0.07)	0.32 (0.032)	0.244 (0.016)
	*p* = 200	*N* = 500	**0.312 (0.113)**	0.127 (0.019)	0.195 (0.055)	0.118 (0.007)	0.118 (0.007)
		*N* = 1000	**0.432 (0.144)**	0.158 (0.033)	0.211 (0.052)	0.126 (0.009)	0.118 (0.006)
		*N* = 2000	**0.52 (0.148)**	0.215 (0.061)	0.211 (0.043)	0.141 (0.02)	0.122 (0.006)
		*N* = 5000	**0.646 (0.114)**	0.277 (0.142)	0.246 (0.053)	0.192 (0.02)	0.13 (0.011)
	*p* = 500	*N* = 500	**0.228 (0.114)**	0.061 (0.014)	0.132 (0.052)	0.055 (0.007)	0.051 (0.002)
		*N* = 1000	**0.321 (0.118)**	0.067 (0.016)	0.129 (0.033)	0.051 (0.007)	0.05 (0.003)
		*N* = 2000	**0.429 (0.148)**	0.098 (0.024)	0.138 (0.042)	0.062 (0.007)	0.051 (0.003)
		*N* = 5000	**0.617 (0.117)**	0.212 (0.091)	0.154 (0.033)	0.086 (0.008)	0.055 (0.003)
	*p* = 1000	*N* = 500	**0.167 (0.092)**	0.047 (0.016)	0.089 (0.04)	0.035 (0.008)	0.027 (0.001)
		*N* = 1000	**0.282 (0.123)**	0.041 (0.01)	0.097 (0.034)	0.028 (0.005)	0.026 (0.002)
		*N* = 2000	**0.389 (0.14)**	0.05 (0.015)	0.1 (0.028)	0.031 (0.004)	0.025 (0.001)
		*N* = 5000	**0.579 (0.143)**	0.134 (0.062)	0.114 (0.026)	0.045 (0.005)	0.027 (0.001)
**Σ_2_**	*p* = 50	*N* = 500	**0.434 (0.091)**	0.398 (0.024)	0.418 (0.044)	0.391 (0.026)	0.396 (0.018)
		*N* = 1000	**0.508 (0.086)**	0.399 (0.026)	0.419 (0.041)	0.42 (0.036)	0.404 (0.019)
		*N* = 2000	**0.54 (0.096)**	0.413 (0.028)	0.429 (0.043)	0.438 (0.041)	0.408 (0.028)
		*N* = 5000	**0.629 (0.108)**	0.479 (0.041)	0.465 (0.043)	0.502 (0.069)	0.44 (0.028)
	*p* = 100	*N* = 500	**0.287 (0.076)**	0.228 (0.028)	0.262 (0.053)	0.227 (0.015)	0.221 (0.009)
		*N* = 1000	**0.393 (0.094)**	0.224 (0.024)	0.256 (0.035)	0.231 (0.019)	0.228 (0.011)
		*N* = 2000	**0.436 (0.108)**	0.232 (0.012)	0.258 (0.037)	0.253 (0.031)	0.234 (0.013)
		*N* = 5000	**0.566 (0.114)**	0.256 (0.017)	0.271 (0.027)	0.299 (0.042)	0.252 (0.014)
	*p* = 200	*N* = 500	**0.183 (0.063)**	0.121 (0.018)	0.154 (0.033)	0.117 (0.008)	0.116 (0.008)
		*N* = 1000	**0.276 (0.101)**	0.144 (0.033)	0.162 (0.036)	0.128 (0.011)	0.118 (0.008)
		*N* = 2000	**0.337 (0.109)**	0.128 (0.036)	0.158 (0.027)	0.13 (0.014)	0.126 (0.007)
		*N* = 5000	**0.455 (0.124)**	0.134 (0.008)	0.173 (0.03)	0.166 (0.03)	0.132 (0.009)
	*p* = 500	*N* = 500	**0.11 (0.065)**	0.059 (0.014)	0.085 (0.025)	0.048 (0.004)	0.05 (0.003)
		*N* = 1000	**0.168 (0.074)**	0.059 (0.012)	0.087 (0.024)	0.052 (0.006)	0.048 (0.004)
		*N* = 2000	**0.216 (0.09)**	0.083 (0.025)	0.092 (0.025)	0.058 (0.008)	0.05 (0.003)
		*N* = 5000	**0.348 (0.132)**	0.067 (0.052)	0.106 (0.025)	0.083 (0.009)	0.057 (0.003)
	*p* = 1000	*N* = 500	**0.07 (0.046)**	0.038 (0.014)	0.057 (0.02)	0.028 (0.006)	0.027 (0.001)
		*N* = 1000	**0.113 (0.066)**	0.037 (0.01)	0.056 (0.019)	0.027 (0.005)	0.025 (0.002)
		*N* = 2000	**0.164 (0.066)**	0.042 (0.013)	0.06 (0.017)	0.029 (0.005)	0.026 (0.001)
		*N* = 5000	**0.259 (0.099)**	0.089 (0.051)	0.075 (0.018)	0.041 (0.005)	0.027 (0.001)
			**AUC**				
**Σ_1_**	*p* = 50	*N* = 500	**0.704 (0.066)**	0.703 (0.115)	0.703 (0.075)	0.682 (0.093)	0.696 (0.08)
		*N* = 1000	0.764 (0.054)	0.745 (0.128)	0.766 (0.07)	**0.777 (0.08)**	0.765 (0.084)
		*N* = 2000	0.782 (0.056)	**0.826 (0.111)**	0.798 (0.074)	0.815 (0.056)	0.807 (0.072)
		*N* = 5000	0.828 (0.059)	**0.921 (0.043)**	0.866 (0.066)	0.854 (0.051)	0.862 (0.043)
	*p* = 100	*N* = 500	0.708 (0.066)	0.687 (0.093)	**0.719 (0.068)**	0.674 (0.072)	0.66 (0.092)
		*N* = 1000	0.752 (0.056)	0.73 (0.122)	**0.763 (0.067)**	0.748 (0.048)	0.743 (0.065)
		*N* = 2000	0.782 (0.053)	0.776 (0.145)	0.794 (0.073)	0.797 (0.091)	**0.811 (0.057)**
		*N* = 5000	0.819 (0.047)	0.85 (0.14)	0.845 (0.06)	0.855 (0.054)	**0.872 (0.058)**
	*p* = 200	*N* = 500	0.696 (0.059)	0.685 (0.085)	**0.713 (0.066)**	0.63 (0.08)	0.654 (0.083)
		*N* = 1000	0.748 (0.053)	0.751 (0.083)	**0.77 (0.065)**	0.742 (0.046)	0.726 (0.07)
		*N* = 2000	0.765 (0.043)	0.79 (0.118)	0.8 (0.061)	**0.82 (0.054)**	0.788 (0.072)
		*N* = 5000	0.805 (0.043)	0.828 (0.132)	0.846 (0.058)	**0.867 (0.042)**	0.861 (0.082)
	*p* = 500	*N* = 500	0.698 (0.055)	0.683 (0.085)	0.715 (0.051)	0.643 (0.093)	**0.757 (0.072)**
		*N* = 1000	0.735 (0.048)	0.743 (0.077)	0.769 (0.057)	0.686 (0.074)	**0.774 (0.079)**
		*N* = 2000	0.767 (0.045)	**0.799 (0.066)**	0.793 (0.061)	0.79 (0.065)	0.782 (0.069)
		*N* = 5000	0.793 (0.04)	0.824 (0.133)	0.842 (0.054)	**0.874 (0.055)**	0.847 (0.048)
	*p* = 1000	*N* = 500	0.692 (0.057)	0.707 (0.082)	0.726 (0.062)	0.713 (0.098)	**0.727 (0.068)**
		*N* = 1000	0.729 (0.042)	0.754 (0.069)	**0.761 (0.054)**	0.706 (0.068)	0.734 (0.072)
		*N* = 2000	0.759 (0.046)	**0.803 (0.064)**	0.792 (0.052)	0.733 (0.045)	0.741 (0.063)
		*N* = 5000	0.792 (0.039)	**0.864 (0.06)**	0.823 (0.052)	0.861 (0.062)	0.844 (0.046)
**Σ_2_**	*p* = 50	*N* = 500	0.676 (0.086)	0.66 (0.131)	0.712 (0.086)	**0.725 (0.073)**	0.723 (0.079)
		*N* = 1000	0.745 (0.064)	0.774 (0.097)	0.768 (0.081)	**0.801 (0.075)**	0.769 (0.055)
		*N* = 2000	0.769 (0.067)	**0.844 (0.083)**	0.833 (0.076)	0.832 (0.053)	0.813 (0.061)
		*N* = 5000	0.816 (0.055)	**0.928 (0.045)**	0.889 (0.06)	0.868 (0.073)	0.899 (0.058)
	*p* = 100	*N* = 500	0.69 (0.073)	0.664 (0.096)	**0.713 (0.076)**	0.693 (0.076)	0.686 (0.075)
		*N* = 1000	0.744 (0.056)	0.689 (0.141)	0.759 (0.071)	**0.773 (0.078)**	0.771 (0.064)
		*N* = 2000	0.759 (0.06)	**0.822 (0.081)**	0.794 (0.079)	0.819 (0.06)	0.813 (0.057)
		*N* = 5000	0.809 (0.051)	**0.896 (0.053)**	0.871 (0.06)	0.857 (0.072)	0.873 (0.067)
	*p* = 200	*N* = 500	0.678 (0.069)	0.647 (0.115)	**0.688 (0.065)**	0.642 (0.057)	0.636 (0.089)
		*N* = 1000	0.736 (0.052)	0.719 (0.107)	0.748 (0.068)	**0.749 (0.07)**	0.727 (0.055)
		*N* = 2000	0.753 (0.055)	0.691 (0.148)	0.793 (0.072)	0.795 (0.068)	**0.807 (0.054)**
		*N* = 5000	0.796 (0.05)	**0.881 (0.053)**	0.864 (0.058)	0.858 (0.067)	0.865 (0.047)
	*p* = 500	*N* = 500	0.658 (0.063)	0.653 (0.111)	0.685 (0.065)	0.584 (0.077)	**0.726 (0.079)**
		*N* = 1000	0.726 (0.057)	0.715 (0.089)	0.744 (0.073)	0.708 (0.085)	**0.759 (0.072)**
		*N* = 2000	0.756 (0.053)	0.764 (0.083)	**0.792 (0.062)**	0.779 (0.079)	0.777 (0.068)
		*N* = 5000	0.802 (0.055)	0.72 (0.166)	0.838 (0.063)	**0.869 (0.062)**	0.86 (0.055)
	*p* = 1000	*N* = 500	0.648 (0.062)	0.663 (0.11)	0.67 (0.065)	0.639 (0.08)	**0.804 (0.08)**
		*N* = 1000	0.739 (0.052)	0.716 (0.089)	0.727 (0.058)	0.692 (0.066)	**0.767 (0.047)**
		*N* = 2000	0.753 (0.048)	0.775 (0.066)	0.773 (0.06)	0.745 (0.077)	**0.801 (0.056)**
		*N* = 5000	0.787 (0.045)	0.733 (0.161)	0.831 (0.057)	0.837 (0.071)	**0.843 (0.061)**

**Table A19:** Simulation 3: mean (SD) of C-index.

			CNT	CNL	CT	CPH	RSF
**Σ_1_**	*up* = 5	*N* = 500	**0.57 (0.034)**	0.529 (0.029)	0.55 (0.027)	0.522 (0.017)	0.534 (0.025)
		*N* = 1000	**0.605 (0.027)**	0.546 (0.029)	0.563 (0.024)	0.529 (0.02)	0.541 (0.023)
		*N* = 2000	**0.619 (0.015)**	0.6 (0.054)	0.579 (0.019)	0.549 (0.019)	0.549 (0.014)
		*N* = 5000	**0.628 (0.008)**	0.543 (0.053)	0.6 (0.012)	0.604 (0.012)	0.581 (0.007)
	*up* = 10	*N* = 500	**0.628 (0.034)**	0.588 (0.03)	0.616 (0.032)	0.547 (0.022)	0.577 (0.024)
		*N* = 1000	**0.665 (0.02)**	0.614 (0.025)	0.645 (0.024)	0.576 (0.023)	0.587 (0.018)
		*N* = 2000	**0.683 (0.013)**	0.669 (0.024)	0.659 (0.018)	0.618 (0.023)	0.609 (0.016)
		*N* = 5000	0.689 (0.007)	**0.735 (0.082)**	0.677 (0.009)	0.674 (0.007)	0.653 (0.009)
	*up* = 15	*N* = 500	**0.66 (0.035)**	0.614 (0.028)	0.648 (0.029)	0.571 (0.031)	0.602 (0.025)
		*N* = 1000	**0.697 (0.022)**	0.649 (0.026)	0.681 (0.019)	0.599 (0.024)	0.612 (0.02)
		*N* = 2000	**0.713 (0.012)**	0.701 (0.016)	0.699 (0.013)	0.666 (0.016)	0.649 (0.01)
		*N* = 5000	0.724 (0.007)	**0.81 (0.036)**	0.712 (0.009)	0.708 (0.007)	0.686 (0.009)
	*up* = 20	*N* = 500	**0.68 (0.029)**	0.643 (0.031)	0.678 (0.03)	0.589 (0.025)	0.624 (0.021)
		*N* = 1000	**0.715 (0.017)**	0.671 (0.028)	0.707 (0.021)	0.621 (0.026)	0.637 (0.019)
		*N* = 2000	**0.733 (0.011)**	0.724 (0.014)	0.724 (0.012)	0.693 (0.017)	0.666 (0.011)
		*N* = 5000	0.744 (0.007)	**0.82 (0.027)**	0.735 (0.007)	0.736 (0.007)	0.716 (0.007)
	*up* = 30	*N* = 500	0.692 (0.029)	0.672 (0.03)	**0.705 (0.024)**	0.614 (0.033)	0.647 (0.033)
		*N* = 1000	0.736 (0.019)	0.71 (0.023)	**0.738 (0.018)**	0.644 (0.024)	0.675 (0.017)
		*N* = 2000	**0.76 (0.011)**	0.757 (0.011)	0.755 (0.012)	0.731 (0.008)	0.703 (0.013)
		*N* = 5000	0.775 (0.006)	**0.833 (0.022)**	0.767 (0.008)	0.766 (0.006)	0.75 (0.005)
**Σ_2_**	*up* = 5	*N* = 500	**0.529 (0.027)**	0.517 (0.024)	0.529 (0.026)	0.513 (0.023)	0.521 (0.028)
		*N* = 1000	**0.551 (0.03)**	0.532 (0.025)	0.54 (0.019)	0.517 (0.017)	0.528 (0.016)
		*N* = 2000	**0.585 (0.023)**	0.547 (0.027)	0.55 (0.017)	0.542 (0.012)	0.54 (0.01)
		*N* = 5000	**0.613 (0.009)**	0.537 (0.032)	0.567 (0.01)	0.579 (0.011)	0.564 (0.008)
	*up* = 10	*N* = 500	0.558 (0.028)	0.539 (0.034)	**0.559 (0.026)**	0.525 (0.024)	0.545 (0.018)
		*N* = 1000	**0.595 (0.025)**	0.564 (0.024)	0.582 (0.024)	0.538 (0.012)	0.553 (0.014)
		*N* = 2000	**0.635 (0.017)**	0.611 (0.025)	0.602 (0.015)	0.58 (0.02)	0.581 (0.011)
		*N* = 5000	**0.659 (0.008)**	0.572 (0.063)	0.619 (0.011)	0.635 (0.008)	0.615 (0.009)
	*up* = 15	*N* = 500	0.574 (0.029)	0.559 (0.032)	**0.575 (0.03)**	0.541 (0.021)	0.56 (0.021)
		*N* = 1000	**0.621 (0.026)**	0.59 (0.027)	0.608 (0.024)	0.564 (0.017)	0.569 (0.025)
		*N* = 2000	**0.661 (0.017)**	0.644 (0.027)	0.635 (0.015)	0.611 (0.016)	0.601 (0.011)
		*N* = 5000	**0.688 (0.007)**	0.602 (0.083)	0.652 (0.009)	0.666 (0.01)	0.648 (0.007)
	*up* = 20	*N* = 500	0.579 (0.027)	0.574 (0.031)	0.585 (0.025)	0.551 (0.027)	**0.586 (0.022)**
		*N* = 1000	0.627 (0.025)	0.618 (0.026)	**0.629 (0.025)**	0.57 (0.021)	0.583 (0.015)
		*N* = 2000	**0.678 (0.016)**	0.666 (0.025)	0.655 (0.015)	0.639 (0.014)	0.623 (0.013)
		*N* = 5000	**0.707 (0.008)**	0.654 (0.085)	0.675 (0.009)	0.688 (0.008)	0.668 (0.008)
	*up* = 30	*N* = 500	0.599 (0.029)	0.59 (0.029)	**0.611 (0.031)**	0.559 (0.026)	0.583 (0.026)
		*N* = 1000	0.646 (0.025)	0.64 (0.028)	**0.656 (0.024)**	0.598 (0.02)	0.618 (0.015)
		*N* = 2000	0.7 (0.015)	**0.701 (0.015)**	0.689 (0.015)	0.669 (0.013)	0.655 (0.013)
		*N* = 5000	**0.734 (0.008)**	0.691 (0.09)	0.706 (0.009)	0.716 (0.005)	0.699 (0.009)

**Table A20:** Simulation 3: mean (SD) of variable selection measures.

			CNT	CNL	CT	CPH	RSF
			F_1_				
**Σ_1_**	*up* = 5	*N* = 500	**0.143 (0.139)**	0.037 (0.011)	0.072 (0.039)	0.031 (0.004)	0.029 (0.002)
		*N* = 1000	**0.439 (0.262)**	0.037 (0.011)	0.08 (0.033)	0.031 (0.004)	0.03 (0.002)
		*N* = 2000	**0.689 (0.178)**	0.07 (0.03)	0.081 (0.03)	0.036 (0.006)	0.03 (0.002)
		*N* = 5000	**0.817 (0.049)**	0.106 (0.134)	0.107 (0.025)	0.05 (0.008)	0.033 (0.002)
	*up* = 10	*N* = 500	**0.263 (0.151)**	0.079 (0.017)	0.185 (0.069)	0.06 (0.01)	0.052 (0.002)
		*N* = 1000	**0.491 (0.198)**	0.08 (0.02)	0.207 (0.07)	0.059 (0.008)	0.051 (0.001)
		*N* = 2000	**0.714 (0.172)**	0.126 (0.03)	0.219 (0.067)	0.064 (0.01)	0.053 (0.002)
		*N* = 5000	**0.856 (0.105)**	0.352 (0.135)	0.244 (0.046)	0.094 (0.006)	0.056 (0.002)
	*up* = 15	*N* = 500	**0.352 (0.137)**	0.11 (0.026)	0.242 (0.072)	0.086 (0.012)	0.073 (0.003)
		*N* = 1000	**0.51 (0.174)**	0.115 (0.027)	0.293 (0.074)	0.082 (0.011)	0.072 (0.003)
		*N* = 2000	**0.647 (0.172)**	0.174 (0.039)	0.307 (0.064)	0.099 (0.006)	0.074 (0.003)
		*N* = 5000	**0.825 (0.117)**	0.372 (0.092)	0.34 (0.055)	0.13 (0.008)	0.078 (0.005)
	*up* = 20	*N* = 500	**0.394 (0.122)**	0.147 (0.029)	0.324 (0.091)	0.108 (0.016)	0.09 (0.003)
		*N* = 1000	**0.528 (0.148)**	0.143 (0.034)	0.353 (0.076)	0.111 (0.013)	0.091 (0.002)
		*N* = 2000	**0.647 (0.17)**	0.214 (0.035)	0.378 (0.067)	0.124 (0.01)	0.094 (0.002)
		*N* = 5000	**0.783 (0.145)**	0.371 (0.088)	0.395 (0.056)	0.168 (0.009)	0.1 (0.004)
	*up* = 30	*N* = 500	**0.418 (0.112)**	0.199 (0.041)	0.397 (0.086)	0.153 (0.021)	0.131 (0.004)
		*N* = 1000	**0.564 (0.135)**	0.211 (0.045)	0.441 (0.085)	0.148 (0.016)	0.131 (0.003)
		*N* = 2000	**0.687 (0.154)**	0.277 (0.041)	0.466 (0.078)	0.183 (0.007)	0.135 (0.003)
		*N* = 5000	**0.766 (0.137)**	0.409 (0.073)	0.497 (0.058)	0.234 (0.011)	0.143 (0.005)
**Σ_2_**	*up* = 5	*N* = 500	**0.068 (0.04)**	0.036 (0.01)	0.057 (0.018)	0.029 (0.003)	0.029 (0.003)
		*N* = 1000	**0.125 (0.117)**	0.036 (0.009)	0.058 (0.017)	0.032 (0.005)	0.028 (0.002)
		*N* = 2000	**0.301 (0.21)**	0.048 (0.019)	0.058 (0.019)	0.035 (0.005)	0.03 (0.002)
		*N* = 5000	**0.742 (0.133)**	0.031 (0.004)	0.069 (0.016)	0.047 (0.01)	0.034 (0.003)
	*up* = 10	*N* = 500	**0.138 (0.072)**	0.067 (0.02)	0.113 (0.035)	0.054 (0.008)	0.05 (0.003)
		*N* = 1000	**0.23 (0.11)**	0.07 (0.017)	0.118 (0.035)	0.053 (0.006)	0.051 (0.003)
		*N* = 2000	**0.358 (0.146)**	0.108 (0.026)	0.128 (0.034)	0.062 (0.01)	0.053 (0.003)
		*N* = 5000	**0.685 (0.179)**	0.091 (0.082)	0.15 (0.027)	0.088 (0.006)	0.057 (0.003)
	*up* = 15	*N* = 500	**0.166 (0.073)**	0.1 (0.028)	0.153 (0.045)	0.073 (0.008)	0.071 (0.003)
		*N* = 1000	**0.302 (0.129)**	0.101 (0.026)	0.173 (0.044)	0.079 (0.01)	0.072 (0.002)
		*N* = 2000	**0.44 (0.149)**	0.159 (0.032)	0.188 (0.048)	0.092 (0.011)	0.074 (0.003)
		*N* = 5000	**0.636 (0.157)**	0.183 (0.142)	0.209 (0.029)	0.121 (0.008)	0.081 (0.003)
	*up* = 20	*N* = 500	**0.199 (0.073)**	0.129 (0.031)	0.191 (0.049)	0.101 (0.014)	0.09 (0.004)
		*N* = 1000	**0.315 (0.131)**	0.137 (0.033)	0.225 (0.062)	0.098 (0.013)	0.093 (0.003)
		*N* = 2000	**0.477 (0.144)**	0.202 (0.043)	0.239 (0.048)	0.121 (0.01)	0.094 (0.004)
		*N* = 5000	**0.629 (0.145)**	0.3 (0.171)	0.263 (0.034)	0.153 (0.007)	0.102 (0.004)
	*up* = 30	*N* = 500	**0.253 (0.074)**	0.172 (0.04)	0.259 (0.059)	0.133 (0.016)	0.128 (0.006)
		*N* = 1000	**0.35 (0.122)**	0.188 (0.041)	0.298 (0.066)	0.145 (0.016)	0.132 (0.003)
		*N* = 2000	**0.527 (0.136)**	0.282 (0.05)	0.333 (0.063)	0.173 (0.016)	0.135 (0.002)
		*N* = 5000	**0.672 (0.122)**	0.426 (0.171)	0.353 (0.043)	0.211 (0.009)	0.146 (0.003)
			**AUC**				
**Σ_1_**	*up* = 5	*N* = 500	0.71 (0.085)	0.691 (0.129)	0.741 (0.104)	0.673 (0.111)	**0.787 (0.11)**
		*N* = 1000	0.784 (0.072)	0.765 (0.117)	**0.811 (0.074)**	0.735 (0.113)	0.789 (0.104)
		*N* = 2000	0.83 (0.046)	**0.846 (0.106)**	0.842 (0.061)	0.841 (0.068)	0.838 (0.071)
		*N* = 5000	0.859 (0.012)	0.681 (0.196)	0.858 (0.038)	0.853 (0.064)	**0.89 (0.054)**
	*up* = 10	*N* = 500	0.785 (0.056)	0.794 (0.066)	0.83 (0.062)	0.742 (0.091)	**0.832 (0.042)**
		*N* = 1000	0.848 (0.049)	0.866 (0.051)	**0.889 (0.037)**	0.79 (0.073)	0.857 (0.057)
		*N* = 2000	0.894 (0.036)	0.908 (0.043)	**0.909 (0.027)**	0.904 (0.043)	0.902 (0.042)
		*N* = 5000	0.915 (0.009)	**0.935 (0.107)**	0.916 (0.018)	0.929 (0.036)	0.929 (0.033)
	*up* = 15	*N* = 500	0.799 (0.051)	0.822 (0.055)	**0.838 (0.049)**	0.744 (0.069)	0.853 (0.062)
		*N* = 1000	0.876 (0.038)	0.891 (0.037)	**0.918 (0.033)**	0.81 (0.068)	0.847 (0.052)
		*N* = 2000	0.922 (0.025)	**0.94 (0.028)**	0.934 (0.015)	0.928 (0.028)	0.917 (0.03)
		*N* = 5000	0.941 (0.008)	**0.966 (0.022)**	0.94 (0.012)	0.947 (0.021)	0.941 (0.025)
	*up* = 20	*N* = 500	0.809 (0.053)	0.829 (0.05)	**0.844 (0.048)**	0.763 (0.067)	0.83 (0.046)
		*N* = 1000	0.893 (0.038)	0.899 (0.04)	**0.927 (0.026)**	0.84 (0.049)	0.879 (0.037)
		*N* = 2000	0.942 (0.019)	**0.952 (0.019)**	0.95 (0.011)	0.94 (0.025)	0.938 (0.033)
		*N* = 5000	0.953 (0.009)	**0.972 (0.016)**	0.953 (0.01)	0.963 (0.02)	0.952 (0.017)
	*up* = 30	*N* = 500	0.798 (0.043)	0.833 (0.047)	0.847 (0.042)	0.762 (0.063)	**0.851 (0.053)**
		*N* = 1000	0.896 (0.033)	0.915 (0.031)	**0.939 (0.024)**	0.809 (0.059)	0.883 (0.031)
		*N* = 2000	0.953 (0.015)	**0.966 (0.015)**	0.965 (0.01)	0.961 (0.016)	0.939 (0.017)
		*N* = 5000	0.968 (0.004)	**0.981 (0.012)**	0.968 (0.007)	0.968 (0.014)	0.971 (0.012)
**Σ_2_**	*up* = 5	*N* = 500	0.688 (0.098)	0.668 (0.14)	0.738 (0.083)	0.692 (0.07)	**0.768 (0.118)**
		*N* = 1000	0.767 (0.081)	0.775 (0.11)	**0.822 (0.066)**	0.707 (0.084)	0.759 (0.088)
		*N* = 2000	0.835 (0.047)	0.797 (0.122)	0.849 (0.06)	0.829 (0.09)	**0.867 (0.069)**
		*N* = 5000	0.856 (0.002)	0.732 (0.186)	0.854 (0.047)	0.864 (0.067)	**0.881 (0.06)**
	*up* = 10	*N* = 500	0.74 (0.073)	0.724 (0.13)	0.784 (0.075)	0.697 (0.097)	**0.809 (0.054)**
		*N* = 1000	0.847 (0.05)	0.849 (0.094)	**0.882 (0.044)**	0.793 (0.08)	0.852 (0.07)
		*N* = 2000	0.894 (0.027)	0.898 (0.074)	0.912 (0.032)	0.915 (0.034)	**0.917 (0.036)**
		*N* = 5000	0.918 (0.016)	0.746 (0.203)	0.917 (0.029)	**0.925 (0.028)**	0.921 (0.032)
	*up* = 15	*N* = 500	0.738 (0.055)	0.78 (0.083)	0.781 (0.063)	0.698 (0.077)	**0.812 (0.068)**
		*N* = 1000	0.867 (0.043)	0.883 (0.061)	**0.895 (0.044)**	0.831 (0.07)	0.866 (0.033)
		*N* = 2000	0.932 (0.025)	0.923 (0.076)	**0.942 (0.021)**	0.933 (0.034)	0.93 (0.023)
		*N* = 5000	0.939 (0.007)	0.762 (0.211)	**0.942 (0.018)**	0.949 (0.02)	0.949 (0.025)
	*up* = 20	*N* = 500	0.743 (0.062)	0.78 (0.082)	0.784 (0.055)	0.734 (0.054)	**0.798 (0.063)**
		*N* = 1000	0.862 (0.045)	0.9 (0.043)	**0.915 (0.033)**	0.81 (0.061)	0.884 (0.038)
		*N* = 2000	0.936 (0.023)	0.944 (0.044)	**0.951 (0.016)**	0.948 (0.021)	0.945 (0.021)
		*N* = 5000	0.953 (0.008)	0.847 (0.192)	**0.955 (0.014)**	0.953 (0.014)	0.953 (0.02)
	*up* = 30	*N* = 500	0.727 (0.054)	0.763 (0.059)	0.781 (0.052)	0.683 (0.044)	**0.784 (0.056)**
		*N* = 1000	0.873 (0.04)	0.897 (0.036)	**0.912 (0.033)**	0.831 (0.053)	0.894 (0.038)
		*N* = 2000	0.953 (0.017)	0.965 (0.013)	0.966 (0.013)	**0.968 (0.01)**	0.963 (0.012)
		*N* = 5000	0.969 (0.007)	0.886 (0.172)	0.969 (0.01)	0.967 (0.015)	**0.972 (0.011)**

**Table A21:** Analysis of HGSOC data: sample characteristics.

	N	%
**Age**		
Q1 (age < 53)	894	23.7
Q2 (53 < age < 60)	838	22.2
Q3 (60 < age < 67)	961	25.5
Q4 (67 < age)	1076	28.5
**FIGO Stage**		
Stage I/II	607	16.1
Stage III/IV	3067	81.4
Stage unknown	95	2.5
**Tumor Tissue Sample**		
Ovary	3340	88.6
Omentum	349	9.3
Other	80	2.1

**Table A22:** Analysis of HGSOC data: the 99 genes identified in all 100 random splittings.

AADAC, ABCC2, ADAMDEC1, ADH1B, ADIRF, AFP, ANKRD1, APC2, ATP8B4, BAALC, BCHE, C10orf82, C1orf173, CD27, CD38, CD3D, CD3E, CEACAM5, COL11A1, COL3A1, COL4A6, CTLA4, CXCL10, CXCL11, CXCL9, CYP2C18, DKK4, DNAI1, DUSP4, ESR2, FABP4, FAP, FBN1, FMN2, FOXC2, FOXP3, GAD1, GFRA1, GJB1, GNG4, HBB, HMGA2, HNF1A, HOXA9, IGF2, IGFBP1, IGHM, IL22, IL6, KDM5D, KIT, KRT13, KRT6A, LBP, LGALS14, LGALS4, LIN28B, LOC81691, LUM, MAB21L1, MAK, MAL, MEOX2, MITF, MROH5, MS4A3, MSH4, MYCN, MYOD1, NTN3, NTRK1, OASL, OR1G1, OR51V1, OR52E4, OR52N4, PAX2, PCDH9, PDCD1, PGR, PLA2R1, PLAC4, POSTN, PTGER3, PTH2R, PTPRT, RARRES1, RNASE2, SLAMF8, SLC12A3, SLC2A3, SLC3A1, SOX10, STATH, TAP1, TFF3, TIMP3, TSHR, ZFHX4

**Table A23:** Analysis of BRCA data: sample characteristics.

BRCA (N = 1093)		
**Age**	58.5 (SD = 13.2)	
**FIGO Stage**		
Stage I	90	Level 1 = 182
Stage IA	86	
Stage IB	6	
Stage II	6	Level 2 = 618
Stage IIA	357	
Stage IIB	255	
Stage III	2	Level 3 = 249
Stage IIIA	155	
Stage IIIB	27	
Stage IIIC	65	
Stage IV	20	Level 4 = 33
Stage X	13	
Missing	11	Level 5 = 11

## References

[1] Wu T.T., Chen Y.F., Hastie T., Sobel E., Lange K. (2009). Genome-wide association analysis by lasso penalized logistic regression. Bioinformatics.

[2] Zhou H., Sehl M.E., Sinsheimer J.S., Lange K. (2010). Association screening of common and rare genetic variants by penalized regression. Bioinformatics.

[3] Ma S.G., Huang J. (2005). Regularized ROC method for disease classification and biomarker selection with microarray data. Bioinformatics.

[4] Yue M., Li J., Ma S. (2018). Sparse boosting for high-dimensional survival data with varying coefficients. Stat. Med..

[5] Tadesse M.G., Sha N., Vannucci M. (2005). Bayesian variable selection in clustering high-dimensional data. J. Am. Stat. Assoc..

[6] Hoyle D.C. (2008). Automatic PCA dimension selection for high dimensional data and small sample sizes. J. Mach. Learn. Res..

[7] Meinshausen N., Bühlmann P. (2010). Stability selection. J. R. Stat. Soc. Ser. B.

[8] Cox D.R. (1972). Regression models and life-tables. J. R. Stat. Soc. Ser. B.

[9] Hoeffding W. (1992). A class of statistics with asymptotically normal distribution. Breakthroughs in Statistics.

[10] Goodfellow I., Bengio Y., Courville A. (2016). Deep Learning.

[11] Lecun Y., Bottou L., Bengio Y., Haffner P. (1998). Gradient-based learning applied to document recognition. Proc. IEEE.

[12] Mohamed A.R., Dahl G.E., Hinton G. (2012). Acoustic Modeling Using Deep Belief Networks. IEEE Trans. Audio Speech Lang. Process..

[13] Van den Oord A., Kalchbrenner N., Kavukcuoglu K. Pixel Recurrent Neural Networks. Proceedings of the 33rd International Conference on Machine Learning.

[14] Krizhevsky A., Sutskever I., Hinton G.E. (2017). ImageNet Classification with Deep Convolutional Neural Networks. Commun. ACM.

[15] Yang Z.L., Dai Z.H., Yang Y.M., Carbonell J., Salakhutdinov R., Le Q.V. XLNet: Generalized Autoregressive Pretraining for Language Understanding. Proceedings of the 33rd Conference on Neural Information Processing Systems (NeurIPS).

[16] Srivastava N., Hinton G., Krizhevsky A., Sutskever I., Salakhutdinov R. (2014). Dropout: A simple way to prevent neural networks from overfitting. J. Mach. Learn. Res..

[17] Han S., Pool J., Tran J., Dally W. (2015). Learning both weights and connections for efficient neural network. Adv. Neural Inf. Process. Syst..

[18] Alvarez J.M., Salzmann M. (2016). Learning the number of neurons in deep networks. Adv. Neural Inf. Process. Syst..

[19] Shi G., Zhang J., Li H., Wang C. (2019). Enhance the performance of deep neural networks via L2 regularization on the input of activations. Neural Process. Lett..

[20] Ma R., Miao J., Niu L., Zhang P. (2019). Transformed ℓ1 regularization for learning sparse deep neural networks. Neural Netw..

[21] Ching T., Zhu X., Garmire L.X. (2018). Cox-nnet: An artificial neural network method for prognosis prediction of high-throughput omics data. PLoS Comput. Biol..

[22] Sun T., Wei Y., Chen W., Ding Y. (2020). Genome-wide association study-based deep learning for survival prediction. Stat. Med..

[23] Ren K., Qin J., Zheng L., Yang Z., Zhang W., Qiu L., Yu Y. Deep recurrent survival analysis. Proceedings of the AAAI Conference on Artificial Intelligence.

[24] Lee C., Zame W., Yoon J., Van Der Schaar M. Deephit: A deep learning approach to survival analysis with competing risks. Proceedings of the AAAI Conference on Artificial Intelligence.

[25] Zhu X., Yao J., Zhu F., Huang J. Wsisa: Making survival prediction from whole slide histopathological images. Proceedings of the IEEE Conference on Computer Vision and Pattern Recognition.

[26] Yang J., Lindenbaum O., Kluger Y. Locally Sparse Neural Networks for Tabular Biomedical Data. Proceedings of the International Conference on Machine Learning.

[27] Hao J., Kim Y., Mallavarapu T., Oh J.H., Kang M. Cox-PASNet: Pathway-based sparse deep neural network for survival analysis. Proceedings of the 2018 IEEE International Conference on Bioinformatics and Biomedicine (BIBM).

[28] Yin Q., Chen W., Zhang C., Wei Z. (2022). A convolutional neural network model for survival prediction based on prognosis-related cascaded Wx feature selection. Lab. Investig..

[29] Tian S., Wang C., Suarez-Farinas M. (2021). GEE-TGDR: A longitudinal feature selection algorithm and its application to lncRNA expression profiles for psoriasis patients treated with immune therapies. BioMed Res. Int..

[30] Li Y., Li R., Qin Y., Wu M., Ma S. (2019). Integrative interaction analysis using threshold gradient directed regularization. Appl. Stoch. Models Bus. Ind..

[31] Ma S., Huang J. (2009). Regularized gene selection in cancer microarray meta-analysis. BMC Bioinform..

[32] Li H., Gui J. (2006). Gradient directed regularization for sparse Gaussian concentration graphs, with applications to inference of genetic networks. Biostatistics.

[33] Liu W., Wen Y., Yu Z., Yang M. (2016). Large-margin softmax loss for convolutional neural networks. arXiv.

[34] Yao H., Zhu D.-L., Jiang B., Yu P. Negative log likelihood ratio loss for deep neural network classification. Proceedings of the Future Technologies Conference.

[35] Ma S., Huang J. (2007). Clustering threshold gradient descent regularization: With applications to microarray studies. Bioinformatics.

[36] Pepe M.S. (2003). The Statistical Evaluation of Medical Tests for Classification and Prediction.

[37] Dudoit S., Fridlyand J., Speed T.P. (2002). Comparison of discrimination methods for the classification of tumors using gene expression data. J. Am. Stat. Assoc..

[38] Millstein J., Budden T., Goode E.L., Anglesio M.S., Talhouk A., Intermaggio M.P., Leong H., Chen S., Elatre W., Gilks B. (2020). Prognostic gene expression signature for high-grade serous ovarian cancer. Ann. Oncol..

[39] Gharpure K.M., Lara O.D., Wen Y., Pradeep S., LaFargue C., Ivan C., Rupaimoole R., Hu W., Mangala L.S., Wu S.Y. (2018). ADH1B promotes mesothelial clearance and ovarian cancer infiltration. Oncotarget.

[40] Li N., Zhan X. (2019). Identification of clinical trait–related lncRNA and mRNA biomarkers with weighted gene co-expression network analysis as useful tool for personalized medicine in ovarian cancer. EPMA J..

[41] Li J., Wood W., Becker K., Weeraratna A., Morin P. (2007). Gene expression response to cisplatin treatment in drug-sensitive and drug-resistant ovarian cancer cells. Oncogene.

[42] Wu Y., Chang T., Huang Y., Huang H., Chou C. (2014). COL11A1 promotes tumor progression and predicts poor clinical outcome in ovarian cancer. Oncogene.

[43] Wu Y.-H., Chang T.-H., Huang Y.-F., Chen C.-C., Chou C.-Y. (2015). COL11A1 confers chemoresistance on ovarian cancer cells through the activation of Akt/c/EBPβ pathway and PDK1 stabilization. Oncotarget.

[44] Bronger H., Singer J., Windmüller C., Reuning U., Zech D., Delbridge C., Dorn J., Kiechle M., Schmalfeldt B., Schmitt M. (2016). CXCL9 and CXCL10 predict survival and are regulated by cyclooxygenase inhibition in advanced serous ovarian cancer. Br. J. Cancer.

[45] Gharpure K.M., Pradeep S., Sans M., Rupaimoole R., Ivan C., Wu S.Y., Bayraktar E., Nagaraja A.S., Mangala L.S., Zhang X. (2018). FABP4 as a key determinant of metastatic potential of ovarian cancer. Nat. Commun..

[46] Heinzelmann-Schwarz V., Gardiner-Garden M., Henshall S., Scurry J., Scolyer R., Smith A., Bali A., Bergh P.V., Baron-Hay S., Scott C. (2006). A distinct molecular profile associated with mucinous epithelial ovarian cancer. Br. J. Cancer.

[47] Zhang W., Ou X., Wu X. (2019). Proteomics profiling of plasma exosomes in epithelial ovarian cancer: A potential role in the coagulation cascade, diagnosis and prognosis. Int. J. Oncol..

[48] Wright M.L., Dozmorov M.G., Wolen A.R., Jackson-Cook C., Starkweather A.R., Lyon D.E., York T.P. (2016). Establishing an analytic pipeline for genome-wide DNA methylation. Clin. Epigenetics.

[49] Carlson J.J., Roth J.A. (2013). The impact of the Oncotype Dx breast cancer assay in clinical practice: A systematic review and meta-analysis. Breast Cancer Res. Treat..

[50] Slodkowska E.A., Ross J.S. (2009). MammaPrint™ 70-gene signature: Another milestone in personalized medical care for breast cancer patients. Expert Rev. Mol. Diagn..

[51] Dubsky P., Brase J., Jakesz R., Rudas M., Singer C., Greil R., Dietze O., Luisser I., Klug E., Sedivy R. (2013). The EndoPredict score provides prognostic information on late distant metastases in ER+/HER2− breast cancer patients. Br. J. Cancer.

